# Chemical Manipulation of Abscisic Acid Signaling: A New Approach to Abiotic and Biotic Stress Management in Agriculture

**DOI:** 10.1002/advs.202001265

**Published:** 2020-08-07

**Authors:** Kamalani Achala H. Hewage, Jing‐Fang Yang, Di Wang, Ge‐Fei Hao, Guang‐Fu Yang, Jian‐Kang Zhu

**Affiliations:** ^1^ Key Laboratory of Pesticide & Chemical Biology Ministry of Education College of Chemistry Central China Normal University Wuhan 430079 P. R. China; ^2^ International Joint Research Center for Intelligent Biosensor Technology and Health Central China Normal University Wuhan 430079 P. R. China; ^3^ Collaborative Innovation Center of Chemical Science and Engineering Tianjin 300072 P. R. China; ^4^ Shanghai Center for Plant Stress Biology and CAS Center of Excellence in Molecular Plant Sciences Chinese Academy of Sciences Shanghai 20032 P. R. China; ^5^ Department of Horticulture and Landscape Architecture Purdue University West Lafayette IN 47907 USA

**Keywords:** ABA signaling, agonists, antagonists, chemical manipulation, plant stress

## Abstract

The phytohormone abscisic acid (ABA) is the best‐known stress signaling molecule in plants. ABA protects sessile land plants from biotic and abiotic stresses. The conserved pyrabactin resistance/pyrabactin resistance‐like/regulatory component of ABA receptors (PYR/PYL/RCAR) perceives ABA and triggers a cascade of signaling events. A thorough knowledge of the sequential steps of ABA signaling will be necessary for the development of chemicals that control plant stress responses. The core components of the ABA signaling pathway have been identified with adequate characterization. The information available concerning ABA biosynthesis, transport, perception, and metabolism has enabled detailed functional studies on how the protective ability of ABA in plants might be modified to increase plant resistance to stress. Some of the significant contributions to chemical manipulation include ABA biosynthesis inhibitors, and ABA receptor agonists and antagonists. Chemical manipulation of key control points in ABA signaling is important for abiotic and biotic stress management in agriculture. However, a comprehensive review of the current knowledge of chemical manipulation of ABA signaling is lacking. Here, a thorough analysis of recent reports on small‐molecule modulation of ABA signaling is provided. The challenges and prospects in the chemical manipulation of ABA signaling for the development of ABA‐based agrochemicals are also discussed.

## Graphical abstract

Small molecules that can manipulate abscisic acid (ABA) signaling events are designed, developed, and evaluated. The ABA‐mediated stress adaptive responses of plants are mimicked by small molecules, and the ABA biosynthesis and catabolism events are altered by inhibitors. This approach is a novel way to gain chemical control over plant stress by using safer ABA‐based agrochemicals.

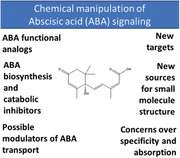

## 1 Introduction

Plant hormone structural analog may serve as a valuable starting point in developing agrochemicals.^[^ ^1^, ^2^ ^]^ Because their natural counterparts have been proven to be capable of regulating plant metabolism structural analogs of plant hormones may have superior performance to that of the natural compounds. The first step in the development of plant hormone‐based bioactive agents is the identification of the hormone signaling mechanism. The study of mechanisms underlying plant hormone signaling has greatly advanced over the past 10 years due to new developments in analytical chemistry, chemical biology, and biotechnology. Five classical phytohormones,^[^ ^3^ ^]^ including auxin (AUX), ethylene (ET), gibberellin (GA), cytokinin (CK), and abscisic acid (ABA), have longbeen recognized as regulators of many aspects of plant growth, plant development, and plant responses to stress. Although basic scientific research on the identification of plant hormone receptors has been conducted for many years, researchers are now finally discovering the mechanisms of hormone perception. With the elucidation of mechanisms underlying plant hormone perception, the development and use of plant hormone‐based agrochemicals are becoming increasingly possible. Plant hormone‐based agrochemicals have a high probability of being perceived by plants because the complex metabolic network that perceives natural hormones in plants is evolutionarily conserved.

Agrochemical discovery guided by plant hormones is becoming increasingly important. Classical plant hormones^[^ ^3^, ^4^ ^]^ were discovered over 40 years ago, but a mechanistic understanding of how plant cells perceive and translate hormone signals has remained a central question in plant biology. In the past decade, however, major classes of the plant hormone receptors have been identified, and chemical signal transduction pathways from hormone perception to response are currently being elucidated. This trend has provided a much clearer view of metabolic processes governed by plant hormones. As a result, the role of the plant hormones in agrochemical discovery is increasing. The reason for this increase may be the need for a nongeneric active ingredient that may be accepted by the general public as safe. The need for new agrochemicals that may be effective over an extended period is also a priority for the agrochemical industry, and an agrochemical guided by natural phytohormones may satisfy the concern for safety and long‐term efficacy.

Management of environmental stresses is essential for improved plant productivity, because biotic and abiotic stresses significantly reduce crop growth and development. Agrochemicals developed for crops have mainly functioned in the management of biotic stressors, but both biotic and abiotic stress factors trigger many responses in plants, which contribute to the reduced yields. In response to a variety of biotic and abiotic stresses, plants synthesize and release ABA. ABA, for example, helps protect plants against extreme temperatures, reduced or excessive water availability, high levels of salinity, cold, high concentrations of heavy metals, and radiation. ABA also helps regulate many fundamental plant developmental processes such as seed development, root growth, senescence, and vegetative‐reproductive phase transitions.^[^ ^5^, ^6^, ^7^, ^8^, ^9^ ^]^ In plants, naturally occurring ABA may simultaneously coordinate growth and development and also adaptive stress responses.^[^ ^10^ ^]^ Therefore, the use of ABA‐based agrochemicals could help crop plants manage environmental stress while maintaining acceptable productivity.

In recent years, ABA signaling in higher plants has been well‐studied for the possibility of improving crop responses to abiotic and biotic stresses via chemical manipulation.^[^ ^11^, ^12^ ^]^ ABA's involvement in multiple and complex events in the plant life cycle has attracted the attention of a diverse group of researchers, including plant physiologists, molecular biologists, and agricultural chemists. The decoding of the ABA signaling pathway was accelerated by the discovery of ABA receptors in 2009.^[^ ^13^, ^14^ ^]^ Researchers have now identified the core ABA signaling components, including ABA receptor proteins (pyrabactin resistance/pyrabactin resistance‐like/regulatory component of ABA receptors (PYR/PYL/RCAR) (hereafter referred to as PYLs), type 2C protein phosphatases (PP2Cs), and sucrose nonfermentation‐1 related subfamily of kinases (SnRK2s), as well as the downstream interacting proteins. Furthermore, the sequential events of ABA perception and phenotypic responses have been confirmed by in vitro reconstitution of the core ABA signaling pathway.^[^ ^15^, ^16^, ^17^ ^]^

Researchers have also developed some important chemical modulators that target critical parts of the ABA signaling pathway. The sulfonamide compound quinabactin^[^ ^5^ ^]^ acts as an effective ABA mimic for the ABA receptor and causes ABA‐like effects in vegetative tissues.^[^ ^5^ ^]^ The broad‐spectrum antagonist AA1^[^ ^18^, ^19^ ^]^ blocks ABA‐bound receptor–coreceptor interactions and antagonizes ABA function. In another example, ABA biosynthesis inhibitors, which target the key enzyme 9‐*cis‐*epoxycarotenoid dioxygenase (NCED) in ABA biosynthesis,^[^ ^20^ ^]^ have been able to reduce ABA accumulation in plant tissues. Several short reviews recently focused on the chemical control of ABA biosynthesis and perception.^[^ ^12^, ^21^ ^]^ Compared to the modulation of ABA biosynthesis and perception via a genetic approach (i.e., via genetic modification), the modulation of ABA signaling via a chemical approach, i.e., via the use of novel agrochemicals, is more straightforward for discovering novel agrochemicals. A comprehensive review of the chemical modulation of ABA signaling, however, is lacking.

Therefore, in our present review, we describe the concept of chemical manipulation, including natural and unnatural chemicals, at signaling cascades of ABA biosynthesis, transport, signaling, and metabolism, which may provide a broader understanding in developing ABA‐based agrochemicals. The complex network of ABA signaling events will be an essential concern in agrochemical development focused on the efficient improvement of plant survival in adverse environmental conditions. The decoding of chemical modulation of the ABA signaling pathway may contribute significantly to designing new ABA analogs. Besides, the model of using small molecules to interact with a broad category of components in the entire ABA signaling in plants may be useful as a reference for other plant hormone signaling control.

## 2 Discovery of ABA: Historical Background

**Figure 1:**
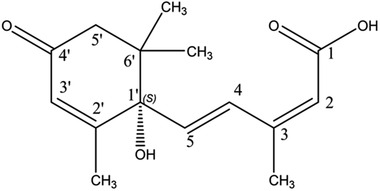
The structure of (+) *S*‐abscisic acid.

The plant growth regulator ABA was discovered by researchers studying fruit abscission. In 1963, Frederick Addicott and co‐workers reported the characteristics of a compound that promoted abscission in cotton fruits, and they called the compound “abscisin ΙΙ.” At the same time, another research group identified a compound that promoted bud dormancy in sycamore trees, and they called the compound “dormin.”^[^ ^22^ ^]^ Later, the two groups collaboratively named the compound abscisic acid. Following this discovery, inhibitory materials in plants were found to increase in wilting plants. In 1969, ABA's ability to close stomata was recognized.^[^ ^23^, ^24^ ^]^ Researchers subsequently discovered many physiological effects of ABA, including responses of plants to environmental stresses, such as drought, cold, salinity, ultraviolet (UV) radiation, and pathogen attack. Although its name implies an “abscission,” ABA does not directly control the formation of the abscission layer in plant tissues undergoing senescence. Instead, it regulates the processes that precede abscission. Other studies proved that plant senescence is mainly regulated by the growth regulator ethylene.^[^ ^25^ ^]^ The investigation of growth regulatory effects and the stress signaling functions of ABA led to additional discoveries about its role in plants. Investigation of the structure of ABA (**Figure** **1**) revealed its reactive‐subgroups, which play an important role in ABA signaling and bioactivity.^[^ ^4^, ^26^ ^]^

The study of ABA perception sites is useful for understanding ABA signaling and physiological functions. Since the identification of plant ABA mutants as early as the 1980s has been extensively studied, and several intracellular ABA receptors have been described. Plant ABA mutants which were identified as early as the 1980s also facilitated the search of ABA receptors.^[^ ^11^ ^]^ The ideal candidate for the ABA receptor required both a stringent structure–activity relationship with ABA and protein active site to initiate and transduce ABA signaling cascade. ABA perception has been extensively studied, and several intracellular ABA receptors have been described,^[^ ^26^ ^]^ however, most of them did not fulfill the requirement for the ideal ABA receptors. Even, some binding assays of putative ABA receptors were questioned, or mutant phenotypes that could not be reproduced in other groups (i.e., the putative ABA receptors were disqualified due to nonsuitable experimental designs, nonreproducibility, and controversial results as well as contrasting response with the extreme ABA insensitive phenotype of *pyl 112458* sextuple mutant).^[^ ^27^ ^]^ These putative ABA receptors cannot act on the ABA signal transfer pathway. Biochemical and genetic approaches were used to identify the ABA‐binding proteins. Initially, researchers used affinity purification to detect proteins (using guard cell membrane extracts) that bound to ABA. They also used a biotinylated ABA derivative as a probe and polyclonal antibodies that recognized ABA receptors.^[^ ^28^ ^]^ These early approaches, however, failed to provide information on ABA signaling. Early genetic approaches focused on loss‐of‐function mutants related to ABA signaling and perception Rather than identifying ABA receptors, however, this approach identified components including transcription factors and protein phosphatases that act coreceptors for ABA perception. Subsequent biochemical and pharmacological studies identified G‐proteins and G‐protein coupled receptor‐like molecules as possible components of ABA signaling, but these components lacked ABA‐binding activity. A protein identified in Arabidopsis and designated as flowering time control protein A (FCA) was described as an ABA receptor.^[^ ^29^ ^]^ None of these efforts, however, described the actual ABA receptor.

The actual ABA receptors were finally identified and described through coordinated efforts of chemical biology and proteomics approaches.^[^ ^13^, ^14^, ^15^, ^30^ ^]^ Addressing the then‐unexplained link between ABA perception and the Abscisic acid insensitive 1 (ABI1) and ABI2 (two of PP2C proteins in ABA signaling pathway) helped reveal plant ABA receptors. A yeast two‐hybrid screen identified At1g01360.1 which was named as RCAR1. Point mutations in PP2Cs could successfully abolish the interactions between RCAR1 and PP2Cs. Studies proved that RCAR1 mediated ABA‐dependent inactivation of ABI1 and ABI2 antagonized PP2C action in plants, indicating the presence of productive ligand–receptor complexes.^[^ ^13^ ^]^ Similarly, the yeast‐two hybrid (Y2H) screen was performed on the PP2C HAB1 to identify interacting proteins. The proteomics approach supported by LC–MS/MS revealed in vivo interacting proteins of ABI1, a key PP2C protein in ABA signaling. Nine of PYLs were identified that strongly interacted with ABI1.^[^ ^31^ ^]^ The breakthrough discovery of the ABA receptor PYR/RCAR/PYL resulted from research on the ABA‐selective agonist pyrabactin. In a chemical genetic screen, PYRABACTIN RESISTANCE 1 (PYR1) mutants were found to be insensitive to pyrabactin.^[^ ^15^ ^]^ A yeast two‐hybrid assay, which used HYPERSENSITIVE TO ABA 1 (HAB1) or ABI 1/2, confirmed that the PYLs are the specific ABA receptors.^[^ ^30^ ^]^ In addition, research on the mechanisms of ABA biosynthesis, transport, perception, and degradation has generated substantial information about PYLs. The large and diverse collection of ABA receptors and their interacting proteins have made the understanding of ABA signaling challenging. We discuss the pathways of ABA signaling in detail in the following chapters.

## 3 The Regulatory Effects of ABA on Plant Physiology

**Figure 2:**
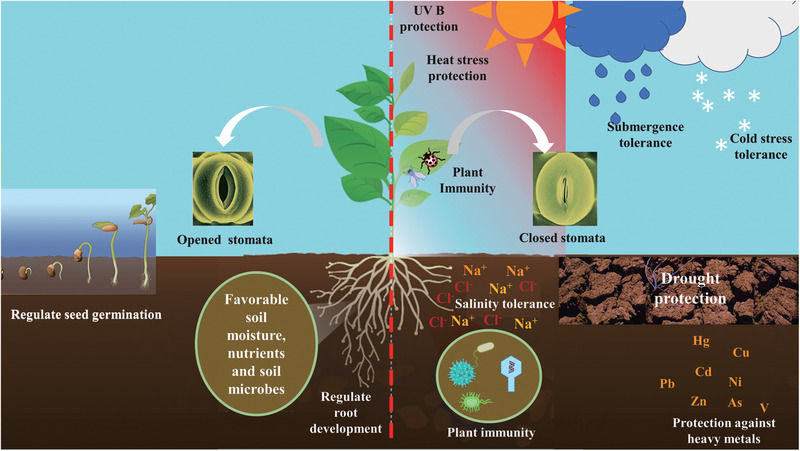
A summary of ABA involvement in plant development and stress tolerance.

ABA has significant roles throughout a plant's life cycle. From the single‐celled zygotic stage to the mature multicellular plant, plant developmental stages involve ABA. ABA allows germination only under optimum conditions and inhibits growth under stress conditions. The adult plant as well as the seedling experience biotic and abiotic stressors that vary in severity and persistence. ABA allows that plant to survive by inducing both short‐term and long‐term stress responses, including rapid and reversible stomatal closure, long‐term growth inhibition, dormancy, senescence, and abscission. ABA is therefore both a developmental and a stress‐signaling molecule with diverse roles (**Figure** **2**), and the effects of ABA are often integrated with those of other plant hormones. We discuss the key biological activities of ABA in the following sections.

### 3.1 ABA as a Developmental Signal

ABA regulates diverse processes of plant development and growth under no‐stress conditions. These effects range from seed germination to plant senescence.

#### 3.1.1 ABA Involvement in Seed Development

**Figure 3:**
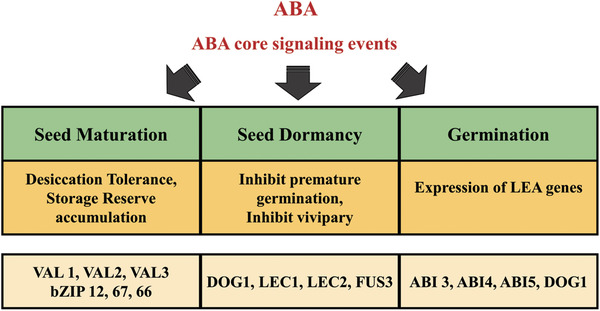
ABA is involved in seed maturation, dormancy, and germination. During seed development, ABA contributes to the accumulation of stored reserves and desiccation tolerance, maintains seed dormancy, and regulates the expression of late embryogenesis abundance (LEA) genes.

As the units of plant survival and dispersal, seeds are important. The single‐celled fertilized zygote resulting from plant reproduction develops into an embryo enclosed in a seed. A seed undergoes three essential stages: development (zygotic embryogenesis), dormancy, and germination (seedling emergence).^[^ ^32^ ^]^ **Figure** **3** illustrates the involvement of ABA and its signaling partners at each stage of seed development.

During seed maturation, ABA prevents premature germination and enhances the accumulation of reserves (**Table** **1**). Two peaks in the ABA content enable optimum seed development.^[^ ^33^ ^]^ The first ABA peak occurs at the onset of seed maturation. At this point, developing embryos cease to exhibit cell division and begin to increase the sizes of their cells by accumulating reserves. The cell cycle of dividing cells is arrested at the G1/S transition by a cyclin‐dependent kinase inhibitor gene, whose expression is induced by maternal ABA.^[^ ^6^ ^]^ This first ABA peak was experimentally detected in ABA‐deficient mutants of tomato and maize. It is therefore evident that an early ABA peak that immediately precedes maturation is essential for preventing early germination. The second embryonic ABA peak occurs in the late maturation phase and is endogenous.

**Table 1:** Key ABA‐related genes involved in seed dormancy and germination

Num.	Gene name	Dormancy level of the respective mutant	Description of the gene
1	*ABI3*	Decreased	Positively regulates ABA signaling; represses germination
2	*ABI5*	Not changed	Positively regulates ABA signaling; represses germination
3	*ABI4*	Decreased	Positively regulates ABA signaling; represses germination
4	*NCED5*	Decreased	ABA biosynthesis gene
5	*CYP707A1/2*	Enhanced	Involves in ABA metabolism
6	*MYB96*	Decreased	Decreases ABI4 and some ABA biosynthesis gene transcription
7	*SUVH4/SUVH5*	Enhanced	Represses ABI3 transcription
8	*WRKY41*	Decreased	Directly promotes ABI3 transcription
9	*DEP*	Decreased	Promotes ABI3 transcription
10	*ARF10/ARF16*	Decreased	Promotes ABI3 transcription
11	*BIN2*	Not mentioned	Phosphorylates and stabilizes ABI5 to enhance ABA signaling
12	*PKS5*	Not mentioned	Phosphorylates ABI5 (Ser42), controls transcription of ABA responsive genes

This peak is essential for both the induction of dormancy and the transition from the embryonic state to germination. The ABA receptor PYL5, which is prominently expressed in seeds, may contribute to ABA perception at this developmental stage.^[^ ^4^ ^]^ ABA directs accumulation of storage reserves (e.g., proteins and lipids) and increases desiccation tolerance in a maturating seed. During seed maturation, ABA induces the expression of transcription factors LEAFY COTYLEDON 2(LEC2), FUS3, and ABSCISIC ACID INSENSITIVE 3 (ABI3) (all of which contain a B3 domain) in Arabidopsis, an ABI3‐like factor in *Phaseolus vulgaris*, and VPI in *Zea mays*.^[^ ^10^ ^]^ These transcription factors then activate genes whose expression increases seed storage reserves, such as late‐embryogenesis‐abundance (LEA) genes.^[^ ^6^ ^]^ Consistent with these findings, genetic analyses revealed the following six classes of transcription factors essential for ABA‐ or seed‐specific expression (ABSISIC ACID INSENSITIVE 3/ABI3/VP1, ABSISIC ACID INSENSITIVE 4/ABI4, ABSISIC ACID INSENSITIVE 5/ABI5, LEC1, LEAFY COTYLEDON 2/LEC2, and FUS3).^[^ ^6^ ^]^ ABA involvement in seed maturation, therefore, facilitates optimum development of subsequent plant stages.

ABA maintains the mature seeds in a dormant state. Dormancy prevents seeds from early germination, which ensures the survival of the plant by coordinating dormancy and germination with seasonal fluctuations between favorable and unfavorable environments. From an agricultural viewpoint, nondormant seeds may germinate prematurely, causing yield decreases and reduced grain quality. Seed dormancy is desirable if seeds are stored before planting later in the growing season. On the other hand, a high level of dormancy causes uneven germination and variable yields. Therefore, a spatially and temporally regulated intermediate dormancy is essential for high yields. The metabolism and signaling events of ABA in seeds are thought to mediate ABA‐related seed dormancy. Endogenous ABA‐mediated repression of germination stabilizes the dormant state of the seed.^[^ ^32^ ^]^ Studies of seeds treated with ABA biosynthesis inhibitors revealed that de novo ABA biosynthesis is involved in maintaining seed dormancy.

ABA‐mediated seed dormancy may be explained by the expression patterns of ABA biosynthesis and signaling components.^[^ ^34^ ^]^ In Arabidopsis, the ABA biosynthesis genes *NCED6* and *NCED9* are responsible for ABA accumulation during seed development and dormancy.^[^ ^35^ ^]^ During the release of dormancy, researchers have documented the catabolism of ABA by CYP702A2 in the embryo and endosperm.^[^ ^36^ ^]^ The genes that function in germination are regulated by key transcription factors. The expression of these transcription factors is induced by ABA and maintains dormancy. The quiescence of the embryo results from the action of transcription factor ABI4, which has embryo‐specific expression.^[^ ^36^ ^]^ ABI5, which is activated by ABA, is expressed in the micropylar region of the endosperm. By inhibiting the expression of enzymes that weaken cell layers and that thereby enable radicle emergence, ABI5 prevents germination. ABI5 accumulation maintains the desiccation tolerance of the seed. Expression of ABI5 is post‐translationally regulated by E3 ligases (KEEP ON GOING (KEG), DROUGHT‐INDUCED WAX ACCUMULATION 1 (DWA1), DROUGHT‐INDUCED WAX ACCUMULATION (DWA2), and SIZ1) via ABSCISIC ACID INSENSITIVE 5 (ABI5) ubiquitination or sumoylation.^[^ ^37^ ^]^ Transition out of seed dormancy is regulated by the antagonistic interactions between ABA and GA.^[^ ^34^ ^]^

As indicated in the previous paragraph, changes in ABA levels in seeds allow them to germinate. Seed germination is controlled by a complex regulatory network. The events of germination are both antagonistically and synergistically controlled by plant growth regulators such as ABA and GAs. Seed dormancy is controlled by the action of ABA, whereas germination is initiated and completed by GA antagonism of ABA.^[^ ^6^ ^]^ A higher quantity of ABA is released from the seed coats of dormant seeds than from nondormant seeds. De novo synthesis of ABA in the endosperm is correlated with dormancy maintenance.^[^ ^38^ ^]^ During germination, ABA levels that maintained seed dormancy are reduced by ABA hydrolases,^[^ ^39^ ^]^ mainly CYP707A2,^[^ ^40^ ^]^ in the embryo and endosperm.^[^ ^40^ ^]^ A collection of transcription factors contribute to ABA‐regulated seed germination. Seed germination and postgermination developmental arrest are mediated by the WRKY2 transcription factor, whose accumulation is induced by ABA. WRKY2 expression requires ABI5, ABI3, ABA2, and ABA3. During germination, a catabolic degradation of ABA results in a decrease in transcription factors responsible for germination arrest. For example, ABI5, which is a basic leucine zipper (bZIP) transcription factor, inhibits seed germination, and early seedling establishment. During postgermination, ABI5 is proteolytically degraded, thereby allowing further plant development. If an increase in ABA level occurs at this point, it can result in ABI5 accumulation and the reactivation of embryonic genes and thereby cause developmental arrest.^[^ ^41^ ^]^ Genes that are essential in seed dormancy and germination have been previously reviewed and listed.^[^ ^42^ ^]^ In summary, proportional changes in ABA levels regulate germination and post‐germination growth.

#### 3.1.2 ABA in Root Growth and Development

While interacting with other plant hormones, ABA inhibits the elongation and proliferation of root cells and maintains the root quiescent center. ABA modulates root cell length, the transition from cell proliferation to differentiation, and lateral root development.^[^ ^43^ ^]^ During root development, ABA effects result from ABA's interaction with AUX, ET, Ca^2+^, reactive oxygen species (ROS), and with cell cycle processes in general. Developing roots contain different zones that are regulated by different phytohormones. A root's stem cell niche, proximal meristem, transition zone, and elongation/differentiation zone are controlled by growth regulators. ABA acts on the stem cell niche zone, where the quiescent center is surrounded by stem cells.^[^ ^44^ ^]^ The quiescent center consists of about 1000 cells in the root apical meristem. These cells divide very slowly or not at all, hence the name quiescent or “quiet, resting.” Following root cell damage, however, the cells regain meristematic activity. ABA action is thought to maintain the quiescence of the quiescent center of the stem cell niche. ABA also acts on the proximal meristem and inhibits cell division by regulating the expression of cyclins and of cell cycle‐dependent protein kinase (CDK) inhibitor proteins. Thus, the action of ABA on the elongation/differentiation zone is inhibitory.^[^ ^44^ ^]^

The ABA signaling components (discussed in the following chapter) are involved in the inhibition of root cell growth, where the ABA receptor protein PYL8 plays a distinct role^[^ ^45^ ^]^ In Arabidopsis, the ABA receptor genes *AtPYR1*, *AtPYL1*, *AtPYL2*, *AtPYL4*, *AtPYL5*, and *AtPYL8* facilitate ABA‐mediated inhibition of root growth. PYL8 directly interacts with the transcription factors MYB77, MYB 44, and MYB73. The renewed growth of lateral roots after ABA inhibition is promoted by PYL8 independent of ABA–SnRK2 signaling. Therefore, the ABA signaling component PYL8 protein is recognized as using a noncell autonomous mechanism in root ABA perception.^[^ ^45^ ^]^ The CDK inhibitor gene *KIP‐RELATED PROTEIN 1(KRP1)/INHIBITOR OF CYCLIN DEPENDENT KINASE 1(ICK1)* is inhibited by ABA, resulting in the inhibition of the G1/S cell cycle transition. The expression level of the cell cycle B‐type cyclin gene is downregulated by ABA, and the down‐regulation affects the G2/M cell cycle checkpoint. Auxins are the main regulators of root development. A gradient of auxin concentration along the primary root facilitates root elongation. High ABA concentrations suppress auxin carrier genes, i.e., the gene *AUXIN RESISTANT 1(AUX1)* that transports auxin into cells and the genes *(PIN FORMED‐1 (PIN1), PIN FORMED‐3 (PIN3), PIN FORMED‐4 (PIN4)*, and *PIN FORMED‐7 (PIN7)* that transport auxin out of cells. During root development, ethylene is involved in regulating auxin biosynthesis, metabolism, and signaling. Under various stress conditions, elevated ROS levels in cells negatively affect auxin signaling. Primary root growth inhibition has been connected with ABA activation of ROS synthesizing enzymes (the nicotinamide adenine dinucleotide phosphate (NADPH) oxidases) in roots and with ABA interplay with auxin signaling. ROS produced by ABA action may activate Ca^2+^‐permeable ion channels and result in elevated Ca^2+^ in cells, which may inhibit root growth.^[^ ^46^ ^]^ ABA enhances the development of hydrophobic suberin barriers in water‐stressed roots, so that water and nutrient flows are controlled.^[^ ^47^ ^]^ In an adult plant root, the casparian strip (abundant with the hydrophobic compound suberin) acts as a barrier that limits the diffusion of minerals, water, and nutrients from the xylem and phloem. Exogenous ABA has been found to induce suberin biosynthesis genes. Mutants defective for ABA biosynthesis and signaling, including *aba2*, *abi3, abi4*, and *abi5*, demonstrated significantly delayed suberin deposition in the endodermis.^[^ ^48^ ^]^ Therefore, a tight interplay of cellular events and components is necessary for ABA action in root development. The prevalent stress condition at a particular root development stage determines endogenous ABA levels and thereby the response of root growth.

#### 3.1.3 ABA in Senescence

Senescence (biological aging), which is the final stage in plant growth and development, can either be genetically programmed or stress‐induced. Chlorophyll degradation and reduced rates of photosynthesis and protein synthesis in senescing tissues cause significant morphological changes in the plant. The metabolic recycling in plant parts undergoing senescence nurtures young and developing parts and storage tissues of the plant. Senescence can be advanced by drought, heat, and other stressors. Senescence is tightly regulated by plant growth regulators, i.e., it is delayed by CK and AUX but promoted by ABA, ET, and jasmonic acid (JA).

In a plant's life cycle, the abscission and senescence of leaves is both a developmental step as well as a long‐term survival response mediated by ABA. Senescing tissues undergo nutrient recycling, i.e., the nutrients in senescing tissues are transferred to storage organs and meristems to maintain plant viability. The upregulation of senescence‐associated genes (SAG) during leaf senescence has been documented.^[^ ^49^ ^]^ Plant‐specific NAC‐transcription factors impose tight control over SAG gene expression. These transcription factors (NAM, ATAF1, CUP‐SHAPED COTYLEDON 2 (CUC2), Oresara 1 (ORE1),^[^ ^50^ ^]^ Oresara 1 sister 1 (ORS1),^[^ ^51^ ^]^ Arabidopsis NAC‐LIKE protein (AtNAP),^[^ ^52^ ^]^ PIF4, and PIF5^[^ ^53^ ^]^) are thought to be regulated by environmental signals as well as by the plant growth regulators. SAGs are involved in nutrient recycling during senescence. ABA can promote leaf senescence by inducing the expression of *ORE1*, *AtNAP*, and *OsNAP* genes. ABA was found to promote leaf senescence through the receptor protein PYL9 and related PP2Cs, SnRK2 protein kinases, ABFs, and RAV1 transcription factors. Increased expression of RAVI and ABF transcription factors requires SNRK2‐mediated phosphorylation. The activated NAC transcription factors mediate downstream functions of SAGs, promoting senescence.^[^ ^49^ ^]^ Upregulated levels of receptor‐like kinase 1 *(RPK1)* were detected during leaf senescence. The *COLD REGULATED (COR)* and *RESPONSIVE TO DEHYDRATION (RD)* genes are regulated by the NAC transcription factor VND‐INTERCTAING 2 (VNI2).^[^ ^54^ ^]^ ABA could also mediate chlorophyll degradation by upregulating *NON YELLOWING 1(NYE1), (NON YELLOWING 2) NYE2*, and *PHEOPHORBIDE A OXYGENASE (PAO)* genes and thereby increasing the expression of ABF transcription factors.^[^ ^55^ ^]^ Therefore, ABA functions in senescence via core ABA signaling and via activation of transcription factors that promote SAG gene expression.

### 3.2 ABA as a Stress Signal

ABA‐mediated stress responses are vital for plant survival under stress. The earliest and most rapid ABA response is the reduction in stomatal aperture size, which helps separate the plant interior from adverse conditions outside. A reduction in stomatal aperture size leads to the activation of several plant defense systems that are often interconnected. Stomatal closure mainly controls transpiration rates, resulting in reduced transpiration and water loss. Stomatal closure is also an innate immune response that prevents pathogen entry. Long‐term or gradual stress responses lead to the induction of senescence and dormancy, prolonged shoot growth, and inhibition of root growth to reduce the effects of the adverse condition. In the following sections, we consider the adaptive stress responses by ABA in terms of the different stress‐factors: drought, cold, pathogen attack, heavy metal stress, submergence, UV stress, and desiccation.

#### 3.2.1 Functions of ABA in Drought

Insufficient levels of soil water can result in an imbalance of water between the cells and the outer environment. A resulting change in the cellular electrolyte content affects metabolism, resulting in an osmotic imbalance or stress. The osmotic stress thus leads to the accumulation of ABA in cells and triggers ABA signaling.^[^ ^56^ ^]^

The cellular pool of ABA is dramatically increased during drought. Biosynthesis, catabolism, conjugation, and transportation of ABA are coordinated to increase ABA levels. ABA biosynthesis enzymes are most abundant in parenchyma cells and vascular bundle cells.^[^ ^57^, ^58^ ^]^ Companion cells in the vascular tissues are also prominent sites of ABA biosynthesis. The autonomous ABA biosynthesis ability of guard cells, however, is regarded as essential for rapid plant responses under drought.^[^ ^59^ ^]^ The root system recognizes water stress, leading to shooting localized ABA synthesis and bioactivity, which results in the regulation of stomata via guard cell action. ABA in guard cells consists of autonomously synthesized ABA, ABA transported from roots to shoots,^[^ ^60^, ^61^ ^]^ and ABA synthesized in the previously mentioned tissues and then imported into guard cells.^[^ ^61^ ^]^

##### ABA‐Mediated Control of the Stomatal Aperture

ABA rapidly regulates plant water levels by controlling stomata. Stomata, which are microscopic pores controlled by two highly differentiated epidermal cells (guard cells), have the primary role in regulating gas exchange between the air and plant. Open stomata allow CO_2_ to diffuse into the leaf mesophyll and reach the sites of photosynthesis. They also allow water vapor to exit from the plant interior to the atmosphere. By allowing transpirational water loss, stomata allow the cooling of the plant and the managing of the interior water levels. Thus, stomata are essential regulators that connected the plant interior to the outside environment. Increases in osmotic pressure in guard cells lead to water uptake and then to cell expansion; as the cells expand, the pore opens because of differential thickenings of guard cell walls. Stomatal movements are regulated by numerous environmental signals such as light, plant growth regulators, pathogens, drought, cold, and nutrient status. Stomatal movement is the quickest response to ABA signaling.

**Figure 4:**
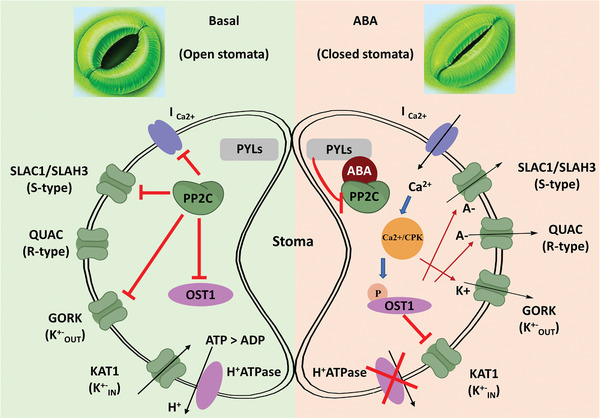
Simplified representation of ABA‐mediated core signaling events in guard cells. In the absence of ABA, (left) PYLs are in ligand free form. H^+^ATPase action pumps H^+^ ions outside of plasma membrane. The SnRK2 protein kinases and the S‐type anion channel SLAC1 are kept dephosphorylated by PP2Cs. The dephosphorylation state of SLAC1 prevents the nonspecific activation of S‐type anion channels. In the presence of ABA (right) PYLs bind to and inhibit PP2Cs.ABA inhibits H^+^ATPase activity, blocking the H^+^ pumping outside. The Ca^2+^‐independent protein kinases (SnRK2s) are released from PP2C inhibition and activated by auto‐phosphorylation. Ca^2+^‐permeable cation (I_Ca_) channels are released from PP2C‐mediated inhibition, causing increases of ABA‐responsive Ca^2+^ in cytosol leading to activate CPKs. The activated SnRK2s and CPKs phosphorylate SLAC1 The SnRK2.6/OST1 protein kinase phosphorylates and activates the R‐type anion channel ALMT12/QUAC1. The K^+^ ions are effluxed via voltage‐dependent outward K^+^ (K^+^_out_) channel GORK, causing a guard cell turgor decrease leading to stomatal closure. PYLs: ABA receptors; ABA: abscisic acid; PP2C: protein phosphatase 2C proteins;OST1: open stomata 1/SnRK 2.6 protein kinase; Ca/CPK: Ca^2+/^calcium dependent protein kinases; I_Ca2+_: plasma membrane nonselective cation channel permeable to Ca^2+^ SLAC1: slow anion channel‐associated 1 (SLAC1); QUAC: aluminum‐activated malate transporter 12/quickly activating anion channel 1 (ALMT12/QUAC1); GORK: guard cell outward rectifying K^+^ channel (GORK); KAT1: K^+^ activated 1 potassium ion channel; A−: anions; K^+^: potassium ions.

ABA signaling for stomatal regulation involves ABA core signaling components and plasma membrane‐located ion channels. Under nonstress conditions or at basal ABA levels, the SnRK2 protein kinases and plasma membrane slow anion channel 1 (SLAC1) are kept dephosphorylated by PP2Cs. The H^+^ ATPase activity results in pumping the H^+^ ions outside the guard cells and hyperpolarizes the guard cell membrane and acidifies the apoplast. The above results in the activation of inward K^+^ rectifying ion channels and thereby K^+^ uptake.^[^ ^62^ ^]^ Intermittent increases of nonspecific Ca^2+^ ions in nonstressed cytoplasm do not lead to stomatal closure. In their dephosphorylated state, the S‐type anion channels are prevented from nonspecific activation.^[^ ^63^ ^]^ In contrast, ABA inhibits the activity of H^+^ ATPase (that provides the driving force for other plasma membrane transporters such as anion channels participating in stomata function) which leads to depolarization of plasma membranes, as H^+^ ions are prevented from being pumped outside.^[^ ^64^ ^]^ ABA signaling events activate the Ca^2+^‐independent protein kinases, and the SnRK2s, including SnRk2.6/Open Stomata 1/OST1. The kinase activity of SnRK2 proteins further activates several downstream targets. They include NADPH oxidases RESPIRATORY BURST OXIDASE HOMOLOG F (RbohF) and RESPIRATORY BURST OXIDASE HOMOLOG D (RbohD), the vacuolar anion exchanger CLCa, plasma membrane anion efflux channels SLAC1 and SLAC1‐homolog proteins 1 and 3 (SLAH1 and SLAH3). Outward‐rectifying potassium (K^+^) channels such as the Gated Outwardly Rectifying K^+^ channel (GORK) are also activated.^[^ ^65^ ^]^ The Ca^2+^‐permeable cation channels are released, increasing cytoplasmic Ca^2+^, which activates Ca^2+^‐dependent protein kinases (CPKs). Activated SnRK2s and CPKs phosphorylate SLAC1. The R‐type anion channel ALMT12/QUAC1 is activated by SnRK2 protein kinase OST1‐mediated phosphorylation. The activation of anion channels leads to plasma membrane depolarization, and the K^+^ ions are effluxed via the voltage‐dependent outward K^+^ channel GORK. A reduction in guard cell turgor pressure due to K^+^ and anion efflux leads to stomatal closure.^[^ ^62^, ^66^, ^67^ ^]^ ABA's tight involvement in stomata regulation is further supported by studies of the *ost2‐1* mutant of H^+^‐ATPase. The mutant guard cells of *ost2‐1* were completely sensitive to ABA despite their responsiveness to darkness and high CO_2_. The mutant possesses an “open stomata” phenotype due to constitutive H^+^‐ATPase activity and it was revealed that the anion channels alone are insufficient to sustain plasma membrane depolarization, characteristic of closed stomata. The finding that ABA is capable of inhibiting H^+^‐ATPase, which is suggested as a primary proton transporter that drives S and R anion channel activity in the plasma membrane signifies ABA's direct involvement in stomata regulation.^[^ ^64^ ^]^ Therefore, the core ABA signaling is essential for guard cell function. The involvement of ABA signaling events in guard cell function is summarized in **Figure** **4**.^[^ ^67^ ^]^

##### Gradual ABA Responses during Drought

In addition to inducing the rapid closing of stomata in response to water stress, ABA induces gradual adaptive responses to drought. Researchers have documented two temporal responses of gene expression to drought.^[^ ^68^ ^]^ An early response (expression peak at 3 h) and late, continued response (from 10 h onward).^[^ ^68^ ^]^ During the early response, the abundant gene products include transcription factors and protein kinases. Also abundant in the early response are the products of *ERD* genes, the functions of which are mostly unknown. The late‐responsive genes include *RD, COR*, low temperature‐induced (*LTI*), and cold‐induced (*KIN*) genes. Some of these gene products are structurally similar to the LEA proteins expressed during the desiccation tolerance of seeds. Other gene products include proteases, chaperonins, enzymes for solute metabolism and ROS detoxification, as well as ion and water channel proteins. The gradual stress responses during drought may be induced by the collective effect of the above proteins and several different metabolic reactions.

At the transcriptional level, distinct signaling modes regulate rapid and gradual plant drought responses. These modes may be ABA‐dependent or independent. One ABA‐dependent mode relies on the core ABA signaling, in which a collection of transcription factors, termed ABA‐responsive element‐binding factors (ABFs), regulate gene expression. ABFs contains a bZIP domain and four conserved domains with sites for Ser/Thr kinase phosphorylation by SnRK2s. Environmental stress factors induce the expression of ABFs, and their transcriptional activity is controlled by ABA‐dependent phosphorylation. Four AREB/ABF transcription factors, AREB1/ABF2, AREB2/ABF4, ABF3, and ABF1, have been found to regulate most downstream genes of subclass III SnRK2s in ABA‐dependent gene expression. ABFs regulate the expression of ABA‐responsive genes by binding to a conserved *cis*‐element, named the ABA‐responsive element (ABRE) (PyACGTGG/TC). Promoters of ABA‐responsive genes harbor the ABREs. These promoters with ABREs act as the main *cis*‐acting elements for ABA signaling under drought and also under other osmotic stress conditions. The ABA‐dependent mode of the drought‐stress response may also be regulated by NAC, WRKY, and MYC/MYB‐transcription factor‐mediated gene expression. However, transcriptional regulation in short‐ and long‐term stress responses mediated by core ABA signaling remain vital for plant survival under drought.^[^ ^69^ ^]^

#### 3.2.2 ABA in Salinity Stress

Salinity is abiotic stress that greatly reduces global crop production.^[^ ^70^ ^]^ Poor irrigation and land‐use practices lead to increased soil salinity. Saline soils are characterized by electrical conductivity of ≥4 dS m^−1^.^[^ ^71^ ^]^ High salinity inhibits seed germination, root and shoot elongation, and fruit/seed development.^[^ ^72^ ^]^ Plants in a highly saline soil initially undergo osmotic stress. When the plant can no longer maintain ion homeostasis in response to the increased ion concentration, plants also undergo ionic stress. These two primary stresses lead to secondary stresses, among which oxidative stress is the most harmful. The combination of osmotic, ionic, and oxidative stress significantly reduces plant productivity. Researchers identified a two‐phase response in plants to salinity stress.^[^ ^70^, ^73^ ^]^ Stomata closure and shoot growth reduction due to inhibited cell expansion takes place within minutes to days. This first phase of response is an ion‐independent growth reduction. A second response spanning from days to weeks has also been observed, where ion levels rise to the cytotoxic level, ceased metabolism, and premature senescence leading to cell death.^[^ ^70^, ^73^ ^]^

Because it triggers both primary and secondary stress responses, salinity is a complex signal. Plants use several mechanisms to tolerate salinity, such as ion transport and uptake, ion homeostasis and compartmentalization, synthesis of antioxidant compounds, and activation of antioxidant enzymes.^[^ ^74^ ^]^ Plants also use phytohormonal control and the synthesis of osmoprotectants, compatible solutes, and endogenous nitric oxide to respond to salinity stress.^[^ ^74^ ^]^ In‐plant hormone control of salinity, ABA may have two functions: the balancing of water by guard cell regulation and dehydration tolerance.

ABA effects on saline‐stressed plants are directed at reducing osmotic, ionic, and oxidative imbalances. The osmotic stress imposed by high levels of salinity leads to rapid increases of endogenous ABA in roots and shoots. In *Vicia faba* exposed to high salinity, ABA levels peaked within 15 min in roots and within 30 min in leaves. As discussed earlier, ABA signaling in guard cells triggers rapid stomatal closure. The ABA content in leaves of glycophytes increased two‐ to three‐fold under salinity stress, but halophytes maintained a relatively constant leaf ABA content.^[^ ^75^ ^]^ Therefore, stomatal regulation may require lower ABA concentrations for halophytes than for glycophytes.^[^ ^75^ ^]^ ABA‐induced H_2_O_2_ production may serve as an additional signal for stomatal closure. Under salinity stress, osmoprotectant production in plants was facilitated by ABA. The osmotic balance maintained by osmoprotectant compounds prevents cell plasmolysis. Proline is vital in this regard, and also acts ROS and which also affect antioxidant activity. ABA was found to increase the expression levels of polyamine biosynthesis genes. Polyamine synthesis enzymes, when overexpressed, increased the expression of NCED and transcription factors involved in ABA signaling. Proline is vital in this regard, and it also acts as a source of energy and nitrogen under stress conditions. Proline synthesis under ABA regulation includes induced expression of Δ_1_‐pyrroline‐5‐carboxylate synthase (*P5CS*) and stabilizing the *P5CS* transcripts.^[^ ^76^ ^]^ During salinity stress, ABA‐mediated accumulation of H_2_O_2_ leads to high levels of nitric oxide (NO).^[^ ^77^ ^]^ High NO levels lead to the up‐regulation of mitogen‐activated protein kinase (MAPK) cascades. MAPK upregulates antioxidant enzymes inalso essential for the full volved in ROS scavenging.^[^ ^77^ ^]^ Therefore, ABA triggers several complex and interconnected responses to osmotic, ionic, and oxidative imbalances in salinity‐stressed plants.

#### 3.2.3 ABA in Cold Stress Response

Cold stress is yet another factor that limits plant productivity as well as plant geographical distribution. Temperatures below freezing can cause “freezing stress,” and temperatures between the freezing point and 15 °C can cause “chilling stress.” Under cold stress, enzymatic activity governing vital metabolic processes such as photosynthesis, respiration, and ROS scavenging are inhibited. As a result, ROS can accumulate and inhibit metabolic reactions. Freezing stress leads to the formation of intercellular ice crystals, which causes dehydration of the plasma membrane and therefore to osmotic stress.^[^ ^78^ ^]^ For cold‐acclimation responses, plants accumulate osmolytes, and antifreezing proteins, and also alter cell membrane composition. Plasma membrane fluidity changes due to cold stress initiated cold‐stress signaling.^[^ ^79^, ^80^, ^81^ ^]^ In other words, low temperatures induce stress signaling, which helps protect the plant from physiological damage. Cold responses of ABA may result from the expression of cold‐responsive genes. The compound “dormin,” which was discovered as ABA, was found to increase freezing tolerance in trees and to be associated with the cold acclimation of other plants.^[^ ^80^ ^]^ Because ABA biosynthesis genes were not affected by cold treatments in Arabidopsis, it has been suggested that early ABA biosynthesis may not occur in response to cold stress. ABA may be required, however, at later stages of cold stress, which are accompanied by metabolic changes that enhance the induction of cold‐responsive genes.^[^ ^80^ ^]^ The cold‐responsive genes (*COR*), which are also induced by dehydration, contain DRT/CRT motifs. The expression of COR genes (e.g., *RD29A, RD22, COR15A, COR47*, and *P5CS*) requires the *cis*‐acting element ABRE.^[^ ^82^ ^]^ A significant increase in the AREB/ABF transcription factor ABF1 (required for ABA‐mediated signaling) was observed after cold stress.^[^ ^83^ ^]^ The MYC and MYB transcription factors regulate the expression of ABA‐response genes under low‐temperature conditions.^[^ ^84^ ^]^ In rice, OsMYB3R‐2 was induced by cold, drought, and salt stresses.^[^ ^85^ ^]^ Together, these findings suggest that ABA facilitates *COR* gene expression under cold stress so as to generate adaptive responses.

Cold stress‐induced signaling molecules can also contribute to ABA signaling. Cold stress activates a transient influx of Ca^2+^ into plant cells. Early abiotic stress responses utilize Ca^2+^ as a signaling molecule. The influx of Ca^2+^ activates protein kinase cascades, which act on transcription factors governing stress‐responsive pathways. Ca^2+^ is also essential for the full expression of *COR* genes. Calmodulin or calcium sensors such as calcineurin B‐like (CBL) proteins transmit the Ca^2+^ signal to activate downstream calcium‐dependent protein kinases (CPKs). The CBL‐interacting protein kinase genes (*CIPKs*) are strongly induced by cold but not by drought. Because ABA‐responsive gene expression was significantly inhibited in the cold‐stressed *cipk3* mutant, CIPK3 may connect ABA with cold and other abiotic stresses.^[^ ^80^ ^]^ The CBL1/CIPK1 and CBL9/CIPK1 complexes have been found to mediate the ABA‐independent and ABA‐dependent responses.^[^ ^80^, ^86^ ^]^ Transient increases in Ca^2+^ during cold stress induce ABA‐dependent cold responses in plants.

Drought, cold, and high salinity often threaten the survival of terrestrial plants, and these stresses often occur together. Drought can reduce plant growth by reducing photosynthetic rates, by causing osmotic stresses that interfere with metabolic processes and proportions of available nutrients. Salinity would impair ion toxicity as well as a condition of physiological drought. Low temperatures as well as salinity cause osmotic stress. Each of these stress conditions is capable of causing osmotic imbalance and desiccation of the plant cell.^[^ ^87^ ^]^ Therefore, the stress‐responsive gene networks for each of the stress factors often overlap. The genes induced by cold, drought, salinity, and exogenous ABA share common elements or control points, suggesting the involvement of ABA in a variety of stress responses.^[^ ^83^ ^]^

#### 3.2.4 Role of ABA in Plant Immunity

Because plants coexist with pathogens (e.g., viruses, oomycetes, bacteria, fungi, insects, and nematodes), plants require pathogen defense or immune systems. Pathogens can be biotrophs, necrotrophs, or hemibiotrophs.^[^ ^88^ ^]^ Plants have multiple layers of pathogen resistance in the form of physical and chemical barriers. A pathogen that overcomes the resistance, are dealt with genetic reprogramming of plant cells.^[^ ^89^ ^]^ Plants have two main types of defenses against pathogens. In one type of defense, pattern recognition receptors (PRRs) in the plant recognize pathogen‐associated molecular patterns (PAMPs), which are evolutionary conserved, resulting in PAMP‐triggered immunity (PTI). PTI is the first defense response. In a subsequent defense response (the second type of defense), plant resistance proteins recognize pathogen effectors, leading to effector‐triggered immunity (ETI).^[^ ^90^ ^]^

The ABA involvement in plant immunity is more complicated than its involvement in abiotic stress responses. ABA functions in pathogen defense may often be interconnected with SA, JA, and ET whose signaling is more dedicated to pathogen defense than is ABA signaling.^[^ ^88^ ^]^ ABA may trigger stomatal closure to block pathogen entry. In addition to modulating other hormone systems and triggering defense responses, ABA also promotes callose accumulation in plant cells that limits pathogen spread. Arabidopsis plants, for example, showed increased callose accumulation that was mediated by an ABA‐dependent defense pathway; the latter pathway also induced expression of *β*‐aminobutyric acid (BABA), a compound that provides resistance against necrotrophic pathogens.^[^ ^74^ ^]^ In response to the pathogen *Pythium irregulare* in Arabidopsis, ABA‐induced callose deposition and inhibited the transcription of callose‐degrading enzymes (e.g., basic *β*‐1,3‐glucanase that acts on the *β*‐1,3‐glucan callose). ABA, on the other hand, can also induce susceptibility to pathogens. For example, the activation of host ABA signaling by the pathogen may suppress host defense mechanisms, leading to disease progression.^[^ ^91^ ^]^

Virulence factors of pathogens may inhibit host plant ABA signaling and thereby facilitate pathogenesis. Oxalic acid secreted by *Sclerotinia sclerotiorum* (the causal agent of foliar wilting in *Vicia faba*) counteracted stomatal closure by ABA. Virulence factors, especially oxalic acid secreted by many plant pathogenic fungi, triggered stomatal opening and increased transpiration and wilting.^[^ ^92^ ^]^ The bacterial pathogen *Pseudomonas syringae* pv. *tomato* produces the phytotoxin coronatine, which counteracts ABA‐dependent defenses by reopening closed stomata. In the case of some pathogen infections, endogenous ABA levels increase. Those pathogens capable of inducing specific signaling systems can increase endogenous ABA levels in plants, and the elevated ABA levels suppress the expression of resistance genes. *Pseudomonas syringae* pv. *tomato*, for example, secreted an effector that strongly induced *NCED* and *ABI1* genes in the host, leading to ABA accumulation; the increased ABA levels suppressed the expression of defense genes and increased the susceptibility to the bacterium.^[^ ^93^ ^]^ ABA also interacts with the other hormones directly involved in plant immunity (JA, ET, and SA). SA‐/ET‐/JA‐dependent immune responses against the necrotrophic pathogen *Plectosphaerella cucumerina* were negatively regulated by ABA.^[^ ^94^ ^]^

#### 3.2.5 ABA in Heavy Metal Stress

Heavy metals inhibit plant growth, and agricultural soils are slight to moderately contaminated with heavy metals due to the long‐term use of fertilizers, incorrect watering practices, and other human activities. Some of the heavy metals (metals and metalloids with an atomic number >20 and a density >4 g cm^−3^)^[^ ^95^ ^]^ are essential plant micronutrients. However, excessive concentrations of heavy metals generate major abiotic stress. As a primary response to heavy metal stress, plants may overproduce ROS. ROS will interact with the electron transport chain and antioxidant enzymes and affect essential metabolic processes. Lipid peroxidation caused by heavy metals, for example, leads to biomembrane destabilization.^[^ ^96^ ^]^

High concentrations of heavy metals in soil can result in increases in ABA biosynthesis and signaling. Several authors have found that the high reactivity of heavy metals causes them to adversely affect plant growth, photosynthesis, and senescence.^[^ ^97^ ^]^ In addition to their direct effects on plant metabolism, heavy metals bring about indirect effects by inducing the overproduction of ROS.^[^ ^97^, ^98^ ^]^ They also interfere with the ROS produced during healthy plant metabolism. ABA biosynthesis genes were upregulated by arsenic (As) stress, while vanadium (V) also induced genes responsible for ABA signaling and biosynthesis. Treatment with cadmium (Cd), copper (Cu), mercury (Hg), nickel (Ni), lead (Pb), or zinc (Zn) resulted in increased endogenous ABA levels. In cucumber plants, Cu and Zn treatment‐induced PYL, PP2C, and SnRK2 genes, which are crucial in ABA signal transduction.^[^ ^98^ ^]^

#### 3.2.6 Role of ABA in Submergence

The submergence of roots in waterlogged soils can severely reduce crop production. Submergence limits the exchange of gases (e.g., O_2_, CO_2_, and C_2_H_2_) between plant tissues and the atmosphere. In addition to reducing gas diffusion, submergence can increase pathogen infection, osmotic and salt stress, and the accumulation of toxic end‐products while reducing transpiration and nutrient uptake and transport.^[^ ^99^ ^]^ By reducing aerobic respiration and photosynthesis, submergence results in an energy‐starved condition. Adaptive phenotypes, such as elongated shoots, increased adventitious roots, and aerenchyma, and processes such as ethanolic fermentation in submerged tissues are mediated by ethylene accumulation. Prolonged submergence, however, results in the death of most terrestrial plants.^[^ ^100^ ^]^

ET, which is the dominant plant hormone in plant submergence responses, acts with ABA and GA during submergence. The adaptive responses in submerged rice are mostly governed by ET‐dependent regulatory processes. In the adaptation of rice plants to submergence stress, ET signaling is linked with ABA and GA responses. When subjected to submergence, the gas exchange between a rice plant and the atmosphere is reduced, which causes the plant to produce more shoots that extend toward the air‐water interface.^[^ ^101^ ^]^ Submergence of rice plants also induces the formation of adventitious roots. Because this additional growth requires energy, the carbohydrate metabolism of submerged rice plants increases; this increase in carbohydrate metabolism includes a breakdown of starch and an increase in anaerobic respiration. These processes are mediated by elevated ET signaling. ABA effects are antagonistic to ET effects. Exogenous ABA, for example, inhibited the shoot elongation promoted by ET and GA. In rice, endogenous ABA levels rapidly decreased during submergence. ABA 8‐hydroxylase was found to degrade ABA in submerged rice plants. Ethylene treatments and submergence increased the mRNA levels of ABA 8‐hydroxylase in rice.^[^ ^102^ ^]^ The accumulation of transcripts of ABA biosynthesis genes may also be reduced by submergence Levels of mRNA of the ABA biosynthesis genes. The transcripts of *OsZEP*, *OsNCED1*, *OsNCED2*, and *OsNCED3* genes, for example, are reduced during the submergence of rice plants. The acclimation of rice plants to submergence is coordinated by submergence‐inducible *Sub1A*.^[^ ^103^ ^]^ The decrease in ABA levels during submergence, however, is not dependent on *Sub1A*.^[^ ^104^ ^]^ The rapid decreases of ABA levels maintain the correct proportions of growth regulators in the response of rice plants to submergence.

#### 3.2.7 ABA in UV Stress

Although photosynthesis requires sunlight, UV light (wavelengths 100–400 nm) can harm plants and other organisms. UV light, which accounts for about 10% of the solar radiation, is assigned to three main types based on wavelength: UV‐A (315–400 nm), UV‐B (280–315 nm), and UV‐C (100–280 nm).^[^ ^105^ ^]^ Atmospheric oxygen and ozone absorb most of the UV‐A and UV‐B wavelengths, but a small fraction of UV‐B reaches the surface of the planet. Because UV‐B is strongly absorbed by genetic material, especially deoxyribonucleic acid (DNA), the genetic material is likely to be damaged by UV‐B. UV‐B‐induced DNA damage results in mutations or cell apoptosis.

In addition to damaging DNA, UV‐B radiation causes oxidative stress in plants. UV‐B radiation disrupts metabolism and photosynthetic electron transport, giving rise to ROS such as superoxide/O^2−^, peroxide/O_2_^2−^, hydrogen peroxide/H_2_O_2_, hydroxyl radical/OH, and hydroxyl ion/OH^−^. The increased levels of ROS damage proteins, lipids, and DNA, and thereby reduce cellular integrity and alter plant morphology and physiology.^[^ ^106^ ^]^ By damaging DNA and causing water radiolysis, UV radiation can also generate hydroxyl radicals (OH^•^), which are the most reactive ROS. Ready removal of redundant OH^•^ radicals is essential for maintaining cellular ROS homeostasis and for preventing cell damage. ROS is an especially complex abiotic stress, because they induce a cascade of damage in plants.

In plants, oxidative stress caused by radiation‐induced ROS is alleviated by DNA repair‐associated enzymes and UV‐absorbing molecules. In response to UV radiation, plants produce superoxide dismutase (SOD), catalase (CAT), peroxidase (POD), and “sunscreen” molecules such as cysteine, glutathione, and vitamin C, B, and E^[^ ^107^ ^]^ The UV‐absorbing secondary metabolites (e.g., flavonoids, sinapate esters, and other phenolics) are produced in vacuoles of epidermal cells and may also be induced by UV‐B.^[^ ^108^ ^]^ Anthocyanin levels are increased by UV‐A, while UV‐B increases the levels of lycopene, beta‐carotene, glycoside, and hydroxycinnamic acid derivatives. Free radicals are also scavenged by antioxidants such as vitamin C and E, flavonoids, and phenolic acids. Under oxidative stress induced by UV radiation, plants may respond by increasing the production of DNA repair enzymes and UV absorbing molecules with the aid of stress‐induced signaling molecules.

ABA contributes to UV‐B stress adaptation by facilitating the scavenging of ROS.^[^ ^109^ ^]^ The role of ABA in facilitating ROS scavenging is related to an increase in nitric oxide (NO) production. NO is considered to be a ROS scavenger as well as a signaling molecule in plants under UV stress. NO increases the activity of antioxidant enzymes and modifies cell wall properties. The flavonoid synthesis gene chalcone synthase is coordinately regulated by UV‐B and NO. Flavonoids are secondary metabolites with antioxidant activity and are also UV‐absorbents. In a model proposed to explain ABA and NO involvement in UV‐B stress, endogenous ABA levels are increased upon UV perception. ABA activates NADPH oxidase, which contributes to the production of superoxide (O_2_^−^) in the cells. The O_2_^−^ produced are acted upon by superoxide dismutase to produce H_2_O_2_. Cytoplasmic increases of H_2_O_2_ enhance NO production in a process that is facilitated by nitric oxide synthase and nitrate reductase. In defense against UV, it is therefore evident that ABA is involved in initiating a sequence of free‐radical‐inhibiting events in plant cells.^[^ ^110^, ^111^ ^]^ Other studies have shown that UV‐B radiation exposure increases the production of ET, which causes early senescence of plant tissues. The presence of ABA in the plant tissues reduces ET production and thereby reduces the detrimental effects of the radiation. ABA‐mediated activation of antioxidant enzymes and membrane sterols was observed in grape leaf tissues in response to UV‐B radiation.^[^ ^103^ ^]^ Therefore, UV‐B stress adaptation in higher land plants by ABA may occur through NO, which facilitates ROS scavenging.

In addition to reducing UV‐B stress, ABA treatment was found to induce the production of secondary metabolites, i.e., bisbibenzyls, that protect the liverwort *Marchantia polymorpha* against UV‐C. The latter study also found that exposure to UV‐C increased endogenous ABA levels in the liverwort by 20‐fold ABA levels in *M. polymorpha*, however, were not increased by UV‐B radiation. In contrast to the wild‐type *M. polymorpha*, the over‐expressor of *MpABI1*, which is a negative regulator of ABA signaling, was severely damaged when subjected to UV‐C. These results indicated that ABA is essential in UV‐C stress tolerance in the model liverwort and basal land plant *M. polymorpha*.^[^ ^112^ ^]^

#### 3.2.8 ABA in Desiccation Tolerance of Aquatic Plants

ABA also mediates stress responses in marine algae and other aquatic plants. Desiccation is considered a critical factor determining the distribution and abundance of intertidal seaweeds.^[^ ^97^ ^]^ Under hydrated conditions, a species highly tolerant of desiccation, *P. orbicularis*, was found to have an ABA concentration 4‐fold higher than that of *M. laminarioides* or *L. spicata*. During desiccation, ABA production levels were 6‐ to 7‐fold greater in *P. orbicularis* than in the other two species. ABA also regulated the activation of the antioxidant enzymes in *P. orbicularis* during desiccation.^[^ ^113^ ^]^

## 4 ABA Biosynthesis

### 4.1 The Plant ABA Biosynthesis Pathway

**Figure 5:**
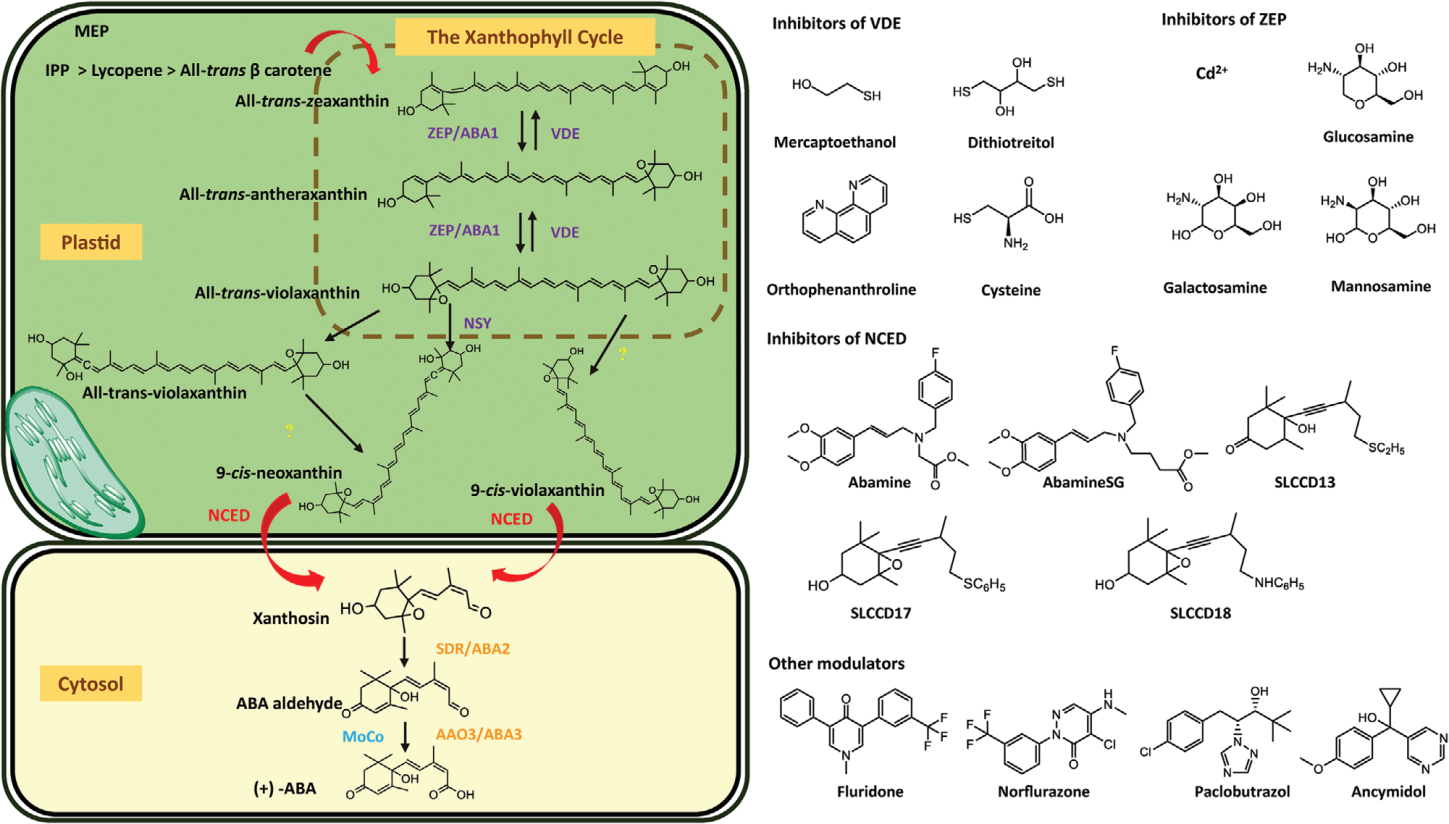
ABA biosynthetic pathways and inhibitors of NCED. 9‐*cis‐*epoxycarotenoid dioxygenases (NCEDs) are attractive targets for chemical modulation in the ABA biosynthesis pathway. The arrows in red represent the processes that can be suppressed by the indicated inhibitors or other modulators. MEP: plastidal 2‐C‐methyl‐d‐erythritol‐4‐phosphate pathway; ZEP, ABA1: zeaxanthin epoxidase; NSY, ABA4: neoxanthin synthase; VDE: violaxanthin de‐epoxidase; SDR/ABA2: short‐chain alcohol dehydrogenase/reductase; AAOs, ABA3: abscisic aldehyde oxidases; MoCo: molybdenum cofactor; IPP: isopentenyl diphosphate.

Plants maintain their endogenous ABA levels by coordinated biosynthesis and catabolism. Biosynthesis is essential for stress‐induced ABA accumulation, which is rapid and substantial, as well as for the regulation of developmental processes in nonstressed plants. At a cellular level, plastids and the cytosol are involved in the sequential steps of ABA biosynthesis. Biochemical genetics has greatly increased our understanding of the ABA biosynthesis pathway in plants. ABA‐deficient mutants of maize (*Zea mays*), tomato (*Lycopersicon esculentum*), tobacco (*Nicotiana tabacum*), potato (*Solanum tuberosum*), barley (*Hordeum vulgare*), and arabidopsis facilitated the unraveling of the ABA biosynthesis pathway. Molecular characterization of defective genes and enzymes of ABA biosynthesis mutants have revealed major intermediates and enzymes for ABA biosynthesis. To date, most of the significant steps in the ABA biosynthesis pathway have been described in detail.^[^ ^114^ ^]^ **Figure** **5** summarizes the plant ABA biosynthesis pathway and chemical modulations of possible targets.

### 4.2 The Spatial Separation of ABA Biosynthesis Reactions and Reactants

In plants, ABA is derived from the carotenoid intermediates formed via the mevalonic‐acid‐independent (MEP: plastidal 2‐C‐methyl‐derythritol‐4‐phosphate) pathway. Biosynthesis of ABA occurs in a sequence of three stages in different cellular compartments: i) the first stage involves the accumulation of small phosphorylated intermediates; ii) the second stage involves the formation of uncyclized C_40_ carotenoid phytoene and the cleavage of 9ʹ‐*cis*‐neoxanthin; and iii) the final stage involves the formation of xanthoxal, which is the C_15_ skeleton required for the formation of ABA.^[^ ^115^, ^116^ ^]^ Based on the cellular compartmentalization of the reactions, the stages of ABA biosynthesis can be discussed in greater detail. In the first stage, carotenoid precursors are synthesized in plastids. In the second stage, xanthophyll is formed and cleaved in plastids. In the final stage, xanthosin is converted into ABA in the cytosol. The occurrence of biosynthesis steps in specific compartments may allow the efficient regulation of cellular ABA levels. ABA biosynthesis is initiated in plastids via sequential steps, ends in the cytosol, and is catalyzed by an array of enzymes. In the early plastidial reactions, ABA biosynthesis overlaps with carotenoid synthesis. The conversion of isopentenyl pyrophosphate (IPP; C5)‐derived geranylgeranyl pyrophosphate (C20) to phytoene (C40 carotenoid) is the first committed and rate‐limiting step in carotenoid synthesis. The plastidial reactions specific to ABA biosynthesis involve three main steps. First, zeaxanthin is converted into all‐*trans* violaxanthin, which is catalyzed by zeaxanthin epoxidase (ZEP).^[^ ^117^ ^]^ This is followed by the formation of 9‐*cis* violaxanthin or all‐*trans* violaxanthin by two catalytic enzymes that have yet to be identified. Next, oxidative cleavage of xanthophylls produces xanthosin via NCEDs. The final products of the plastidial reactions are transported to the cytosol for subsequent steps. As indicated earlier, the last step in ABA biosynthesis occurs in the cytosol. In the cytosol, three pathways convert xanthosin into ABA via abscisic aldehyde, xanthosic acid, or abscisic alcohol. Most of the critical steps of the enzymatic catalysis in both plastidial and cytosolic reactions have been described.

### 4.3 Key Enzymes in ABA Biosynthesis

The catalytic activity of ABA biosynthesis enzymes helps regulate the ABA levels in plants. The early biosynthetic steps (in plastids) are catalyzed by zeaxanthin epoxidase (ZEP/ABA1), neoxanthin synthase (NSY/ABA4), NCED, and violaxanthin de‐epoxidase (VDE). The subsequent biosynthetic steps are catalyzed by short‐chain alcohol dehydrogenase/reductase (SDR), abscisic aldehyde oxidases (AAOs and ABA3), and the molybdenum cofactor (MoCo) in the cytosol (Figure 5).^[^ ^118^, ^119^, ^120^, ^121^ ^]^ NCED is considered the regulatory enzyme in ABA biosynthesis, because its expression is well correlated with endogenous ABA levels. Significant ABA accumulation can be achieved by overexpressing NCED.^[^ ^119^ ^]^ Therefore, NCED is considered the key enzyme in ABA biosynthesis and a critical target of the chemical manipulation of ABA biosynthesis.

#### 4.3.1 Rate‐Limiting Step in ABA Biosynthesis

The enzyme NCED is considered the rate‐limiting enzyme in plant ABA biosynthesis. During ABA biosynthesis, all‐*trans*‐zeaxanthin produced by the MEP pathway is converted to all‐*trans*‐violaxanthin by ZEP.^[^ ^117^ ^]^ all‐*trans*‐zeaxanthin can also be produced from all‐*trans*‐violaxanthin through VDE via all‐*trans*‐antheraxanthin. This reaction is termed the xanthophyll cycle, and it accounts for the nonphotochemical quenching of Chlorophyll *a* fluorescence, which is a means of overexcitation of photochemical reactions for photoprotection.^[^ ^122^, ^123^ ^]^ Part of all‐*trans*‐violaxanthin is then converted into 9ʹ‐*cis*‐neoxanthin by NSY.^[^ ^124^ ^]^ The total epoxyxanthophyll content of Arabidopsis leaves mainly consists of all‐*trans*‐violaxanthin and 9ʹ‐*cis*‐neoxanthin (40.2% and 51.8%, respectively). After that, 9‐*cis*‐violaxanthin and 9ʹ‐*cis*‐neoxanthin (isomerized from all‐*trans*‐violaxanthin and all‐*trans*‐neoxanthin, respectively) are oxidatively cleaved to form the C_15_ product, and the cleavage is catalyzed by NCED (Figure 5).^[^ ^117^ ^]^ The conversion of early intermediates to later intermediates and products in ABA biosynthesis is mainly controlled by NCED.

Several studies have focused on the involvement of NCED in ABA biosynthesis. NCED was initially cloned in maize by insertional mutagenesis.^[^ ^125^ ^]^ Among the nine identified NCED‐related sequences in Arabidopsis, five (*AtNCED2*, *3*, *5*, *6*, *and 9*) may function in ABA biosynthesis.^[^ ^119^, ^126^, ^127^ ^]^ Increased NCED gene expression was observed before drought‐induced ABA accumulation. In contrast, the expression of both endogenous ABA and NCED was rapidly decreased during rehydration after dehydration.^[^ ^128^ ^]^ Multigene families across land plants encode NCED enzymes, with distinct family members having unique roles in developmental processes and stress responses.^[^ ^21^, ^121^, ^126^, ^129^ ^]^ Therefore, NCED involvement in ABA biosynthesis appears to be crucial across the plant kingdom.

#### 4.3.2 Enzymatic Regulation of Cytosolic Steps in ABA Biosynthesis

The final cytosolic events in ABA biosynthesis mainly involve oxidation reactions. The product of plastidial reactions, xanthoxin, is transferred to the cytosol, where it is converted into ABA aldehyde by short‐chain alcohol dehydrogenase (ABA2).^[^ ^117^, ^130^, ^131^ ^]^ During the final step in ABA biosynthesis, the abscisic aldehyde is catalytically oxidized to the carboxylic acid by abscisic aldehyde oxidase (AAO). Among the four AAOs, AAO3 encodes an enzyme active on abscisic aldehyde.^[^ ^132^ ^]^ The requirement of molybdenum cofactor (MoCo) for aldehyde oxidase catalytic activity indicates that mutations in the genes for MoCo biosynthesis may lead to ABA deficiency.^[^ ^119^ ^]^ The final oxidation steps that take place in the cytosol in ABA biosynthesis may be rate‐limited by AAO.

### 4.4 Chemical Manipulation of ABA Biosynthesis

#### 4.4.1 Inhibitors of 9‐*cis‐*Epoxycarotenoid Dioxygenases (NCEDs)

NCED primarily regulates ABA biosynthesis by promoting ABA accumulation. The enzyme oxidatively cleaves 9‐*cis*‐epoxycarotenoids which may be a major regulatory step in the biosynthesis of ABA in higher plants. NCED is therefore an attractive target for chemical manipulation of ABA signaling.^[^ ^119^, ^133^ ^]^

Abamine is an ABA biosynthesis inhibitor with an amine moiety. Among ABA biosynthesis inhibitors, abamine was the first one that was found to target NCED. Abamine competitively inhibits NCED, with a *K*_i_ value of 38.8 × 10^−6^ m during the osmotic stress caused by a 0.4 m mannitol solution. Treatment of spinach (*Spinacia oleracea*) with abamine inhibited stomatal closure, and this inhibition was relieved by the coapplication of ABA. Spinach and Arabidopsis plants treated with 0.4 m mannitol had ≈16‐fold increases in ABA contents relative to controls, but a treatment of (50–100) × 10^−3^ m abamine decreased the mannitol‐induced ABA accumulation by ≈50%. Arabidopsis plants treated with abamine were more drought susceptible than the controls, and the decrease in drought tolerance was in accord with the phenotype of *AtNCED3* knockout mutants.^[^ ^126^ ^]^ Abamine‐treated cress (*Lepidium sativum*) seeds showed increased radicle elongation; because the effect of abamine was reversed by 0.1 × 10^−3^ m ABA, the effect of abamine in decreasing ABA content was evident. Abamine may useful in chemical–genetic approaches to identify mutated genes in ABA biosynthesis.^[^ ^133^, ^134^, ^135^, ^136^, ^137^, ^138^ ^]^

Abamine has served as a basis for finding new inhibitors of ABA biosynthesis. Studies of the relationship between the structure and activity of abamine led to the identification of abamineSG, which inhibits ABA accumulation. The methyl ester and the nitrogen of abamine SG are connected by a three‐carbon linker. ABA accumulation in plants under osmotic stress was reduced 77% by treatment with 100 × 10^−6^ m abamineSG. Under the same conditions, abamine inhibited ABA accumulation by 35%. AbamineSG treatment of osmotically stressed plants strongly inhibited the expression of ABA‐responsive and ABA catabolic genes. The enzyme NCED was competitively inhibited by AbamineSG, with a *K*_i_ value of 18.5 × 10^−6^ m. Although high abamine concentrations (>50 × 10^−6^ m) inhibited the growth of Arabidopsis seedlings, seedling growth was not inhibited by abamineSG concentrations as high as 100 × 10^−6^ m. Because chemicals that inhibit ABA biosynthesis allow rapid, conditional, reversible, selective, and dose‐dependent control of biological functions, they are likely to be more useful than mutant plants that are defective in a biosynthetic function. Such a chemical can be readily evaluated for its functions irrespective of plant species. AbamineSG, however, provided greater inhibition of ABA biosynthesis than abamine, but unlike abamine, abamineSG did not inhibit seedling growth. Thus, abamineSG may be a more suitable chemical modulator than abamine and may be more useful than abamine for elucidating the functions of ABA in plants. In “chemical–genetics” approaches to screening for new mutants, researchers have used an auxin mimic and a brassinosteroid biosynthesis inhibitor.^[^ ^135^, ^136^, ^138^ ^]^ The use of an ABA analog has helped identify novel mutants that affect the ABA sensitivity of Arabidopsis germination and seedling growth.^[^ ^139^, ^140^, ^141^ ^]^ AbamineSG is the first chemical modulator found to decrease ABA levels without negatively affecting normal plant growth. Abamine and related compounds have already been protected by a patent (US 7098365B2). In addition to its use as an ABA biosynthesis inhibitor, abamineSG may be useful for identifying novel ABA mutants with increased ABA sensitivity.^[^ ^142^ ^]^

Sesquiterpene‐like carotenoid cleavage dioxygenase (SLCCD) is another inhibitor of NCED. SLCCDs were obtained by modifying the sesquiterpenoid segment of the 9‐*cis*‐xanthophyll substrates and products. In in vitro assays, three SLCCDs (SLCCD13, SLCCD17, and SLCCD18) inhibited Arabidopsis NCED3 (*AtNCED3*) with *Ki* values of 93 × 10^−6^, 57 × 10^−6^, and 87 × 10^−6^ m, respectively. A model for the inhibition mechanism was obtained by the computational docking of *AtNCED3*, in which the heteroatom coordinates with the nonheme iron in the enzyme's active site.^[^ ^143^ ^]^ In the presence of SLCCD13, Arabidopsis plants osmotically stressed by mannitol accumulated lower ABA levels and catabolites than control plants. SLCCDs moderate ABA‐induced gene regulation. They were only weakly active in the inhibition of seed germination. Interestingly, all three inhibitors were found to moderate the stress‐induced transcription of *AtNCED3*, which may lead to reduced ABA levels in planta. Sesquiterpenoid‐like inhibitors may be useful for the study and control of ABA biosynthesis.^[^ ^143^ ^]^

#### 4.4.2 Inhibitors of Xanthophyll Cycle Enzymes

ABA biosynthesis shares and overlaps with specific steps of the xanthophyll cycle,^[^ ^144^ ^]^ which is controlled by two enzymes: violoxanthin de‐epoxidase and zeaxanthin epoxidase. Potential inhibitors of these enzymes are considered in the following sections.

##### Inhibitors of Zeaxanthin Epoxidase (ZEP)

Both metal ions and sugars can reduce ZEP activity. For example, ZEP activity can be inhibited by cadmium. The effect of cadmium was reversed by zinc ions, which highlighted the importance of a cysteine residue in conserved domains of ZEP.^[^ ^145^ ^]^ In Lemna trisulca (duckweed; a model aquatic plant), zeaxanthin epoxidation by the violaxanthin cycle enzyme ZEP was reduced by >50% by amino sugars (glucose and glucosamine, galactose and galactosamine, and mannose and mannosamine at 0.5% concentration) but not by other sugars after a 24 h incubation; the inhibition was increased as the light phase of the photoperiod increased.^[^ ^144^ ^]^

##### Inhibitors of Violoxanthin De‐epoxidase (VDE)

In a study with isolated spinach chloroplasts, the sulfhydryl reagent dithiothreitol inhibited the ascorbate‐dependent wavelength shift of 505 nm caused by de‐epoxidation of violaxanthinto zeaxanthin. The reversible effect of dithiothreitol was evident for both light‐induced de‐epoxidation at pH 7 and dark de‐epoxidation at pH 5.^[^ ^146^, ^147^ ^]^ However, the inhibition of VDE by iodoacetamide was irreversible.^[^ ^147^ ^]^ Ascorbate has been identified as a cofactor as well as a cosubstrate for VDE and for other xanthophyll cycle enzymes.^[^ ^147^ ^]^ Ascorbate availability was identified as a limiting factor for in vivo VDE activity.^[^ ^148^ ^]^ Another early study, which used sonicated thylakoids from spinach, found that VDE activity was increased by 3‐fold by the addition of monogalactosyldiacylglycerol (MGDG), which is the major lipid in the thylakoid membrane. VDE activity has a pH optimum of 5.2 and requires ascorbate. Dithiothreitol (DTT), mercaptoethanol, cysteine, and o‐phenanthroline (at 0.055 × 10^−3^, 0.68 × 10^−3^, 2.7 × 10^−3^, and 0.025 × 10^−3^ m, respectively) reduced VDE activity by 50%. VDE was also inhibited by zeaxanthin.^[^ ^149^ ^]^ The in vitro inhibition of VDE by DTT and low pH can be reversed by desalting and dilution.^[^ ^149^ ^]^

These findings concerning the inhibition of ZEP and VDE were obtained from studies of the photoprotection of the photosynthetic machinery by the xanthophyll cycle enzymes. However, the possibility of using ZEP and VDE to study ABA signaling would be questionable because these enzymes are effective at an early stage of ABA metabolism. In addition to overlapping with ABA biosynthesis, the xanthophyll cycle is an essential step in the plant photoprotective machinery. Therefore, the xanthophyll cycle enzymes may not be useful targets for chemical manipulation in living plants.

### 4.5 Other Inhibitors

Growth regulators, including cytokinins, and antigrowth regulator compounds such as ancymidol (antigibberellin compound), and paclobutrazol (triazole fungicide; gibberellin antagonist) were found to inhibit ABA biosynthesis in the fungus *Cerospora rosicola*.^[^ ^150^, ^151^ ^]^ However, as observed in cell‐free preparations from the liquid endosperm of wild cucumber, ancymidol and some cytokinins also inhibited gibberellin biosynthesis.^[^ ^152^ ^]^

Some pesticides inhibit ABA biosynthesis. Application of the herbicides fluridone or norflurazone (binding with phytoene desaturase) resulted in ABA deficiency in the treated plants. In response to dehydration, dark‐grown control barley plants had increased ABA levels (30–40‐fold within 4 h), while plants treated with 0.1 × 10^−3^ m fluridone accumulated very little ABA. This finding may be explained by a carotenoid deficiency in the fluridone‐treated plants. Low levels of carotenoids were detected in dark‐grown barley plants treated with 0.31 × 10^−12^ m fluridone. Dehydration of these plants resulted in a 4–8‐fold increase in ABA and a decrease in antheraxanthin, violaxanthin, and neoxanthin, but no change in *β*‐carotene or lutein and zeaxanthin levels.^[^ ^33^, ^153^, ^154^ ^]^ Destruction of chlorophyll results in bleaching of plants and a loss of ABA biosynthesis. Carotenoid deficiency led to pleiotropic phenotypes, such as ABA deficiency, and caused concurrent bleaching of plants due to the destruction of chlorophyll resulting in the loss of ABA biosynthesis.^[^ ^122^, ^153^ ^]^ Therefore, fluridone or norflurazone are not favored as ABA biosynthesis inhibitors. A very low concentration of fenarimol, however, inhibited ABA accumulation in *C*. *rosicola*. However, a 50% reduction in ABA accumulation occurred in response to treatment with 5 × 10^−6^ m fenarimol or 10^−8^ m triarimol^[^ ^33^ ^]^

In addition to growth regulators and pesticides, other chemicals have been found to inhibit ABA biosynthesis. Before NCED was identified, the carotenoid lyase that is related to ABA biosynthesis was originally expected to be a lipoxygenase‐like enzyme.^[^ ^155^ ^]^ Nordihydroguaiaretic acid (NDGA), which was known as an inhibitor for lipoxygenase, catalyzes the deoxygenation of polyunsaturated fatty acid and inhibits the accumulation of ABA in soybean suspension cells subjected to osmotic stress.^[^ ^155^ ^]^ However, NDGA also inhibits lipid synthesis and plant growth.^[^ ^156^ ^]^ Guided by the structure of NDGA, researchers developed a more specific NCED inhibitor, abamine.^[^ ^122^, ^133^ ^]^ In addition, decylimidazole and tridemorph inhibited ABA biosynthesis to varying degrees in *C. rosicola*.^[^ ^157^ ^]^

## 5 ABA Transport

### 5.1 The ABA Transporter Proteins and the Ionic Trap Model

**Figure 6:**
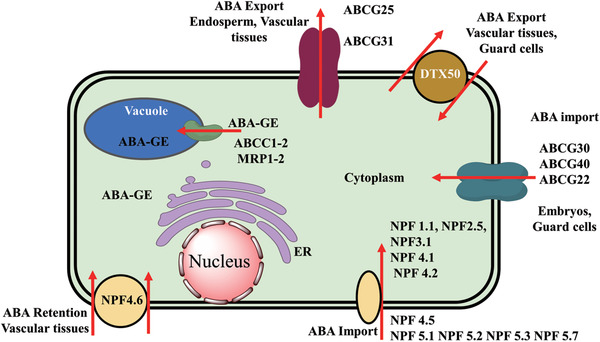
ABA transporter proteins with reference to their influx–efflux activity.

Because ABA has different sites for biosynthesis versus biological activity, the regulatory mechanisms must ensure the optimum ABA availability at the site of perception. Although ABA levels are mainly controlled by biosynthesis and catabolism, the sites of biosynthesis and catabolism are also spatially and temporally separated. The cytoplasmic environment under nonstress and stress conditions affect the content of available ABA. Given the spatial separation, transporter proteins are needed to move the required amount of ABA to their specific sites of action.^[^ ^158^ ^]^ For both ABA and its conjugates, researchers have described both diffusion and transporter protein‐mediated transport (efflux and influx). **Figure** **6** illustrates the described ABA transport events at the cellular level.

The ionic trap model describes ABA transport across cell membranes aided by transporter proteins. ABA (a weak acid, p*K*a 4.7) exists in its ionic form (ABA^−^) and in its protonated form (ABA‐H). ABA‐H freely diffuses through the phospholipid bi‐layers of cellular membranes, but ABAˉ cannot cross membranes via diffusion. ABA‐H travels via the xylem, and because xylem is surrounded by living cells, ABA‐H will continue to diffuse into cells on its way upward to leaves. Under nonstressed conditions, the apoplastic pH of 5.0–6.1 allows ABA to remain in ABA‐H form and therefore to diffuse into the cytoplasm. The cytoplasmic pH of 7.5 under nonstress conditions, however, results in ABA^−^. Under nonstressed conditions, in other words, ABA‐H can freely diffuse into the cytoplasm, where it is converted into a form (ABA^−^) that can no longer diffuse outside: ABA^−^ is, therefore “trapped” in the cytoplasm. As a result, almost no ABA from xylem will reach the guard cells in leaves. The absence of sufficient ABA in guard cells causes stomata to remain open under nonstressed conditions. Under stressed conditions, in contrast, apoplastic pH increases (up to ≈6.7), resulting in increased levels of ABA^−.^ which will reach guard cells via xylem. Although ABA‐H cannot diffuse through guard cell membranes, the transporter proteins on the guard cell membranes allow the intake of ABA‐H into the cells such that ABA levels in the cell become sufficient to cause stomatal closure. Although ABA^−^ cannot diffuse through guard cell membranes, the transporter proteins on the guard cell membranes allow the intake of ABA^−^ into the cells such that ABA levels in the cell become sufficient to cause stomatal closure. The ionic trap model considers two modes of ABA transport: diffusion and transport via transporters. As per the ionic trap model, the transporters have three functions: they allow ABA movement into cells under conditions that limit diffusion; they ensure that ABA is directed to a particular receptor for cell‐specific signaling (achieved by localized expression of transporters); and they enable ABA efflux from sites of synthesis in the xylem and apoplast to other cells and organs.^[^ ^159^ ^]^ According to the ionic trap model, ABA transporters may be especially important under stress conditions.

### 5.2 Transporter Proteins of ABA

The transport of ABA involves either import or export across cell membranes and may require ATP energy. To date, ABA transporters identified in plants include members of the ATP binding cassette (ABC) family, members of the nitrate transporter 1 family (*NRT1*);^[^ ^160^, ^161^ ^]^ and detoxification efflux carrier proteins (DTX). Recent studies and nomenclatures renamed the plant nitrate transporter 1(*NRT1*) or the peptide transporter (*PTR*) gene family as Nitrate and Peptide transporter Family (NPF).^[^ ^162^, ^163^ ^]^ The reported ABA transporter proteins are discussed in the following paragraphs.

#### 5.2.1 ABCG40/PDR12

The Arabidopsis homolog of the ABCG40 transporter protein is mainly localized in plasma membranes of guard cells and has also been found in embryo cells. It functions as an ABA‐uptake transporter. AtABCG40/AtPDR12 was identified based on the germination and stomatal responses of Arabidopsis PDR homozygous knockout mutants (*atabcg29–atabcg41* ^[^ ^164^ ^]^). AtABCG40/AtPDR12 is a full‐size transporter^[^ ^165^ ^]^ with high ABA affinity (*K*_M_ = 1 × 10^−6^ m) and stereo‐specific ABA uptake activity. The transporter has an 8‐fold higher expression in guard cells than in other leaf cells. Exogenous ABA caused the mutant *atabcg40* plants to delay the upregulation of the transcription factors *AtABR1* and *AtRD29B*, and of an ABA biosynthesis enzyme *AtNCED3*. This indicated that the AtABCG40/AtPDR12 gene product is associated with the rapid ABA response. The transporter contributes to efficient ABA intake and responses in guard cells, because *atabcg40* plants had a significantly delayed ABA response. Transcripts of AtABCG40/AtPDR12 were abundant in dissected embryos, but were not abundant in the endosperm.^[^ ^166^ ^]^ Some of the transport functions of AtABCG40 are similar to those of AtABCG30.^[^ ^166^ ^]^ AtABCG40/AtPDR12 was found to be necessary for the importing of ABA into the embryo.^[^ ^166^ ^]^ Therefore, ABCG40/PDR12 may function in the importing of ABA from the endosperm.^[^ ^166^ ^]^ Its higher level of expression in guard cells makes AtABCG40/AtPDR12 important in plant stress responses.

#### 5.2.2 NRT1.2/AIT1/NPF4.6

The ABA transporter protein NRT1.2/AIT1/NPF4.6 is confined to the plant cell plasma membrane. It was identified from a screen of the nitrate transporter family NRT1/PTR (which has 53 reported sequences to date). The cDNA‐induced interactions between the ABA receptor and PP2C in the presence of 0.1× 10^−6^ m ABA was used as a screen in identifying the transporter protein. Although initially characterized as a low‐affinity nitrate transporter, NRT1.2/AIT1/NPF4.6 showed a higher affinity for ABA than for nitrate. Furthermore, the affinity of the transporter was higher for the natural enantiomer of ABA (*K*_M_ ≈ 5× 10^−6^ m) than for the synthetic (*R*) enantiomer. NRT1.2/AIT1/NPF4.6 sustains the ABA pool size in vascular parenchyma cells, which are sites for ABA biosynthesis.^[^ ^158^ ^]^

#### 5.2.3 DTX50

The Arabidopsis homolog of the DTX50 protein is an ABA efflux transporter that is localized in plasma membranes. AtDTX50 promoter activity was found predominantly in vegetative‐vascular tissues (leaves, roots, and germinating seeds) and not in stems, flowers, or siliques. The *AtDTX50* gene was identified by a reverse genetic approach using T‐DNA insertion mutants in Arabidopsis. Treatment with ABA but no other hormones caused severe growth inhibition of the T‐DNA insertion mutant *atdtx50*. AtDTX50 specifically mediated ABA (+/−) efflux but not GA or IAA efflux in three different systems (*E. coli*, *Xenopus* oocytes, and Arabidopsis protoplasts). At the same time, leaf mesophyll cells of *atdtx50* mutant (preloaded with ABA) released less ABA than the wild type. Plasma membrane localization of AtDTX50 was detected by the transient expression of the DTX50: GFP fusion construct under the control of the AtDTX50‐promoter in Arabidopsis protoplasts. The expression of AtDTX50 was induced by ABA.^[^ ^167^ ^]^ Because AtDTX50 is most active at pH values <7.0, its role in ABA efflux is significant. The pH optimum of AtDTX50 falls in the pH range that occurs under drought conditions (in response to drought, xylem and, apoplastic pH increase from 5.2–6.5 to 7.2–7.4). The responses of AtDTX50 to stress may be facilitated by its localization in tissues that synthesize ABA, its pH optimum, and its specificity for ABA.

#### 5.2.4 ABCG25

The Arabidopsis homolog of ABCG25, also known as AtWBC26, is an ABA exporter protein located on the plasma membrane.^[^ ^168^, ^169^ ^]^ It was identified by screening ABA‐related mutants from a transposon‐tagged mutant collection (activator (Ac)/dissociation (Ds) system). An ABA‐sensitive phenotype in germination and seedling stages was characteristic of an identified single mutant. AtABCG25 gene expression was active and overlapped with ABA biosynthesis locations, as revealed by the expression of the AtABCG25 promoter‐driven GUS reporter gene. Kinetic studies revealed that AtABCG25 has stereospecific selectivity for (+) ABA (with a high affinity; *K*_M_ = 260 × 10^−9^ m). Adenosine diphosphate (ADP) inhibited ATP‐dependent ABA uptake. Effects of AtABCG25 on ABA efflux were assessed with a vesicle transport assay in insect cells.^[^ ^168^ ^]^ ABA synthesized in vascular parenchyma cells was exported by ABCG25 to ABA influx transporters, e.g., ABCG40.^[^ ^159^, ^169^ ^]^ Therefore, AtABCG25 is important in the movement of ABA to ABA importers during stress.

#### 5.2.5 Other ABA Transporters

Other proteins are involved with ABA transport. The gene *AtABCG22* encodes a half‐sized protein in the Arabidopsis ABCG subfamily of ABC transporters. ABA is a potential substrate for AtABCG22, given the close position of AtABCG22 and the AtABCG25 ABA efflux transporter in the phylogenetic tree.^[^ ^170^ ^]^ The double mutants of *abcg22/srk2e have* increased susceptibility to drought and increased the water loss of *srk2e*/*ost1* mutants. Evidence of direct ABA import by AtABCG22, however, has yet to be reported.^[^ ^170^ ^]^ Another plasma membrane‐localized transporter, AtABCG31, is highly expressed in the Arabidopsis seed endosperm, but is not expressed in dissected embryos in the presence of the GA synthesis inhibitor paclobutrazol. AtABCG31 is a full‐sized ABC protein and is located in a distant position in phylogenetic clusters, although its substrate is similar to that of AtABCG22, AtABCG25, and AtABCG40. AtABCG31 facilitated ABA efflux from the endosperm to the embryo. AtABCG31 is also expressed in guard cells^[^ ^171^ ^]^ and in the tapetum.^[^ ^172^ ^]^ In Arabidopsis, the AtABCG16 transporter is involved in plant basal resistance against the bacterial pathogen *Pst* DC3000 pathogen as well as in ABA tolerance. Transcription of *AtABCG16* is up‐regulated in the presence of ABA, bacterial infection, and coronatine, which is a bacterial virulence factor.^[^ ^173^ ^]^ An extensive study reclassified the members of the NTF/NPF transporters with respect to their ABA transport function. In the study, the following NTF/NPF transporters were assayed in the presence of 0.1 × 10^−6^ m ABA in a yeast two‐hybrid system: NPF4.6/NRT1.2/AIT1, NPF4.5/AIT2, NPF4.1/AIT3, NPF4.2/AIT4, NPF6.3/NRT1.1, NPF4.4/NRT1.13, NPF4.3/NRT1.14, NPF6.2/NRT1.4, NPF6.4/NRT1.3, NPF6.1, and NPF4.7. The results showed that some of the NPFs were capable of transporting plant hormones in addition to ABA,^[^ ^174^ ^]^ indicating a substrate nonspecificity for ABA in NTF/NPF transporters.^[^ ^162^ ^]^ Therefore, the ABA transporter activities of the ABC family and of NPF transporter proteins require additional research.

### 5.3 Chemical Modulators of ABA Transporter Proteins

Researchers have found that ABA transporter activity in the ABC transporter superfamily proteins can be modulated by typical modulators of ABC transporters. The import of ABA to guard cells via AtABCG40 was altered by chemical modulators of ABC transporters. The latter include glibenclamide/glibenclam^[^ ^175^ ^]^ (an ATP‐sensitive K^+^ channel blocker; most studied in animal cells^[^ ^176^ ^]^), verapamil (a voltage‐dependent calcium channel blocker), and vanadate (an H^+^ ATPase inhibitor).^[^ ^164^, ^177^ ^]^ Stomatal‐opening bioassays and patch‐clamp experiments revealed specific negative modulation of the outward K1 channel by the sulfonylurea compounds glibenclamide and tolbutamide. The sulfonylureas have been long known for their hypoglycemic effect and for their use as therapeutic compounds to treat noninsulin‐dependent diabetes.^[^ ^176^ ^]^ Glibenclamide was found to rapidly and negatively modulate the slow anion current of guard cells in a dose‐dependent manner; a concentration of 3× 10^−3^ m provided half of the maximum negative modulation. Glibenclamide activity was antagonized by cromakalin. Stomatal closure induced by external Ca^2+^ or ABA was reversed by glibenclamide.^[^ ^175^ ^]^

### 5.4 Transporter Proteins for ABA Conjugates and Their Chemical Modulators

ABA conjugates are formed by catabolism, and are stored and transported elsewhere. The primary storage and transport form of ABA is ABA glucose ester (ABA‐GE). ABA‐GE is synthesized in the cytosol by UDP‐glucosyltransferases (as discussed in the following chapter) but has a very low membrane permeability. Researchers have therefore determined whether transporter proteins mediate ABA‐GE transport.

The cellular intake of ABA‐GE is mediated by two methods of membrane transport, i.e., via ABC transporters and via proton gradient‐driven transport. Modulators of ABC transporters and disruptors of proton gradients may act as ABA‐GE transport modulators. The ABC transporters AtABCC1 and AtABCC2 of Arabidopsis were found to have ABA‐GE transport activity in vitro, i.e., in membrane vesicles isolated from yeast when heterologously expressed. ABA‐GE transport mediated by AtABCC1 and AtABCC2 is enhanced when ABA and ABA‐GE levels are increased by exogenous supply of ABA, ABA‐GE, or tetcyclacis (an inhibitor of P450 cytochromes), or a mixture of exogenous ABA and tetcyclacis. Proton‐gradient disruptors reduced ABA‐GE uptake. Ammonium chloride (NH_4_Cl) at a concentration of 5 × 10^−3^ m reduced ABA‐GE uptake by 28%, and 0.5 × 10^−3^ m bafilomycin A1, a vacuolar proton pump (V‐ATPase) inhibitor, reduced ABA‐GE uptake by 43%. A combination of bafilomycin A1 and NH_4_Cl reduced ABA‐GE uptake by 58%. Quercetin, a compound that inhibits ABC‐type and proton antiporters of multidrug and toxic compounds, also reduced ABA‐GE uptake; at 0.5 × 10^−3^ m, quercetin or quercetin‐3‐*O*‐glucoside reduced ABA‐GE uptake by 71% or 60%, respectively. ABA‐GE uptake was enhanced by MgATP but was reduced by 92% by 1 × 10^−3^ m orthovanadate and by 90% by 1 × 10^−3^ m probenecid in AtABCC2‐expressing yeast vesicles.^[^ ^178^ ^]^ As a whole, chemical modulators of ABA‐GE transporters have complex structures and seem to lack substrate specificity. Determining their functions will require additional research.

## 6 The ABA Signaling Core Components

**Figure 7:**
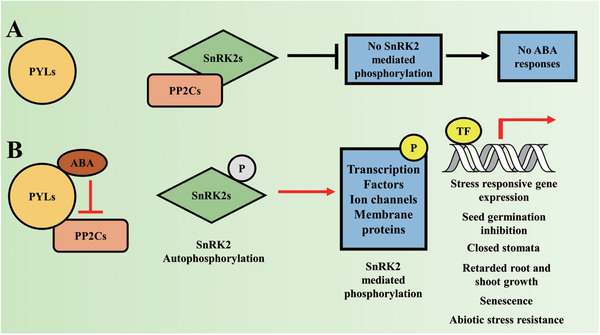
Components of the Core ABA signaling pathway. A) In the absence of ABA, SnRK2 kinases are dephosphorylated by PP2Cs. B) In the presence of ABA, PP2Cs are inhibited by the complexes PYLs‐ABA. Thus, the SnRK2 kinases are released and make a cascade of downstream transcription factors, NADPH transporters, and ion channels phosphorylate the transcription factors induce ABA‐responsive gene transcription, and ion channels act on the guard cells to bring about transpirational control.

The perception of a phytohormone by a receptor is unarguably the most significant event, i.e., the core event, among the sequential signaling events for any phytohormone. There are three main phases concerning ABA signaling. They are ABA synthesis/metabolism, long‐distance transport, and ABA perception followed by long‐distance transport; perception of ABA; and finally signal transduction and ABA signal response and modulation.^[^ ^11^ ^]^ To perceive ABA, the core components comprised of ABA receptors, coreceptors, and protein kinases (**Figure** **7**) function in concert with their downstream substrates in a tightly regulated manner. The immediate downstream targets of the ABA signaling core include several transcriptional activators/repressors and plasma membrane‐located channel proteins. Identification of ABA receptors and their structure has greatly increased our understanding of ABA perception. A high level of specificity and conservation exists among ABA signaling core components. Signal perception itself is an important and large research topic that continues to be studied. The majority of chemical manipulation events for ABA signaling core components are mostly recorded for the ABA receptors. The SnRK2 proteins are subject to chemical manipulation to a lesser level, and the reported chemical manipulation studies are lacking for the direct inhibitors of PP2C proteins. The PP2C type plant phosphatases consist of 76 members that display a high diversity, of which PP2CA members (group A PP2Cs) are directly involved in ABA signaling.^[^ ^179^ ^]^ Even though inhibitors of other phosphatases such as PP1 and PP2A^[^ ^180^ ^]^ are reported, studies on direct inhibitors of PP2Cs for ABA signaling are yet to be discovered.

### 6.1 ABA Perception by the Signaling Core

The key players of ABA perception and subsequent signaling events are, ABA receptors, coreceptors, and protein kinases.^[^ ^181^, ^182^ ^]^ In 2009, researchers using chemical and biological approaches succeeded in the decoding and molecular characterization of the long‐sought core ABA signaling pathway in plants. Several classes of ABA receptors have been identified to date^[^ ^26^, ^183^, ^184^ ^]^ but ABA signaling mediated by pyrabactin resistance protein family of ABA receptors has been accepted as the major ABA signaling pathway.^[^ ^7^, ^22^, ^181^, ^185^ ^]^ These specific ABA receptors termed as Pyrabactin Resistance/Pyrabactin Resistance ‐Like/Regulatory Component of ABA Receptors (generally referred to as PYLs), which was obtained independently by several groups based on structural, biochemical and genetic evidences.^[^ ^14^, ^30^ ^]^ As canonical ABA receptors, it has strong ability to precisely bind to ABA and interact with protein phosphatases and protein kinases involved in ABA signaling.^[^ ^27^ ^]^ These receptors bind ABA to form a ligand–receptor complex; the complex interacts with clade A type protein phosphatases 2C (PP2Cs). The increased stability of the ABA‐PYL‐PP2C complex results in conformational changes in the active sites of PP2Cs. The high stability of the ABA‐PYL‐PP2C complex (IC_50 ATPYR1, PYL1‐PYL11 (ABA)/ATHAB1_: (27–307) × 10^−9^ m; *K*_d ATPYL10 (ABA)/ATABI1_ = 20× 10^−9^ m)^[^ ^186^, ^187^ ^]^ results in competitive inhibition of PP2Cs.This releases a class of downstream protein kinases (SnRK2s), from their PP2C‐mediated inhibition^[^ ^14^, ^185^, ^188^ ^]^ Upon release from the PP2C inhibition, the SnRK2s undergo autophosphorylation to activate a series of ion channels, NADPH oxidases, and transcription factors via phosphorylation. This activates both short‐term and long‐term ABA responses such as stomatal closure and upregulation of ABA‐dependent gene expression. Despite the above information on the proposed model of SnRK2 activation, studies were conducted to assess whether the direct autophosphorylation and/or transphosphorylation events act upstream phosphorylation of SnRK2s for ABA signaling. Mitogen activated protein kinase kinase kinases (MAPK3s) were found to activate SnRK2.6 by phosphorylating a specific site during salinity stress.^[^ ^189^ ^]^ In mosses, SnRK2 activation was dependent on ABA and abiotic stress‐responsive Raf‐like kinase (ARK) acting upstream of SnRK2.^[^ ^190^ ^]^ The Arabidopsis homologs of ARK were found to phosphorylate and activate SnRK2 subfamily III during osmotic stress.^[^ ^191^ ^]^

The overall ABA perception is a double‐negative regulatory system, in which PYLs act as bona fide ABA receptors, PP2Cs as negative‐regulatory coreceptors, and SnRK2s as negative regulators.

During the perception of ABA, the “gate” for the entrance of the ligand‐binding pocket of PYL closes and locks ABA inside. This occurs as a result of conformational changes in two highly conserved loops on PYLs; the altered loops serve as gate and latch. ABA perception is therefore described as a gate‐latch‐lock mechanism.^[^ ^123^, ^192^ ^]^ Further elaborations on the features of gate and latch of ABA bound CsPYL1 and SiPYL1s have revealed a closed latch conformation while the gate remained in a nonproductive open conformation (a stable intermediate state between apo PYL and ABA‐PYL‐PP2C ternary complex), incompatible with ABA binding.^[^ ^193^ ^]^ The intermolecular swiveling in CsPYL1 and SiPYL1 did not take place for the respective dimeric PYL to undergo monomerization upon ABA binding. This finding indicates that closing the gate and latch of PYLs are independent events; instead of the already available explanation where ABA binding, the closing of gate and latch and PYL dimer dissociation are described as coupled events. The above highlights PP2C's role as indispensable co‐receptors,^[^ ^27^ ^]^ as PP2Cs choose to interact only with the gate closed‐latch closed PYL to shift the gate open‐gate closed PYL equilibrium to form the ABA‐PYL‐PP2C ternary complex.^[^ ^193^ ^]^ Other studies on crop ABA receptors (ie. FePYR1 in turfgrass) have also supported this updated model.^[^ ^194^ ^]^To date, 14 members of PYLs in Arabidopsis, PP2C‐A group proteins, and a group of SnRK2 subclass III proteins in Arabidopsis are known to function in the core ABA signaling pathway, along with a 4–9 group of A‐bZIP transcription factors.^[^ ^11^, ^195^, ^196^ ^]^ Our understanding of the core ABA signaling pathway, as illustrated in Figure 7, is based on a vast amount of chemical, genetic, and structural information and on successful reconstitution in vitro.^[^ ^15^, ^17^, ^197^, ^198^ ^]^

ABA signaling core components in plants are tightly regulated. In addition to the positive regulation by SnRK2 and the negative regulation by PP2Cs (**Table** **2**), several post‐translational modification events enable efficient regulation of ABA signaling. Phosphorylation, dephosphorylation, ubiquitination, farnesylation, and sumoylation have been found to modulate ABA signaling by targeting core components (PYLs or PP2Cs) or other interacting proteins downstream. Recent reviews have summarized and discussed the post‐translational regulation events related to the ABA core signaling network.^[^ ^11^, ^47^, ^199^ ^]^

**Table 2:** Chemical modulators of ABA transporters and ABA conjugate transporters

Transporter	Function	Chemical modulator		Chemical modulator mode of action
AtABCG40	Guard cell ABA uptake	Glibenclamide (Glibbenclam)	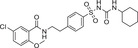	ATP‐sensitive K^+^ channel blocker; reduced ABA uptake
		Verapamil	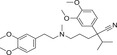	Voltage‐dependent calcium channel blocker
		Vanadate	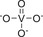	H^+^ ATP‐ase inhibitor; reduced ABA uptake
AtABCC1 AtABCC2	ABA‐GE uptake	Tetcyclacis	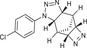	Cytochrome p53 inhibitor Reduced ABA‐GE uptake
		Ammonium Chloride	NH_4_Cl	Proton gradient disruptor, reduced ABA‐GE uptake
		Bafilomycin A1	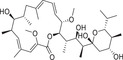	Vacuolar proton pump (V‐ATPase) inhibitor; reduced ABA‐GE intake
		Quercetin	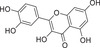	Inhibitor of ABC‐type and proton antiporters of the multidrug and toxic compounds; reduced ABA‐GE uptake
		Quercetin‐3‐*O*‐glucoside	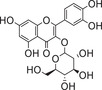	
AtABCC2	ABA‐GE uptake	Orthovanadate	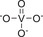	Reduced ABA‐GE uptake
		Probenecid	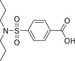	Reduced ABA‐GE uptake
		MgATP	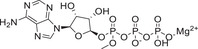	Enhanced ABA‐GE uptake

### 6.2 The ABA Receptors (PYLs)

#### 6.2.1 The Role of PYLs in ABA Signaling

PYLs have been determined to be bona fide receptors for ABA perception. They are widely distributed and highly conserved across the plant kingdom. Although different plant groups show a broad diversity of PYLs, common aspects of their structure and binding mode enable them to perceive the universal stress signal, i.e., the ABA molecule.

PYLs are a conserved family of proteins that perceive ABA. They possess the steroidogenic acute regulatory (stAR)‐related lipid‐transfer domain (START).^[^ ^196^ ^]^ PYLs are soluble proteins, and among the 14 PYLs in *Arabidopsis*, 13 functions as ABA receptors.^[^ ^200^ ^]^ In algae, ABA synthesis has been detected but algal homologs of ABA binding PYLs have not been observed. However, the PYL homolog of *Zygnema circumcarinatum* is capable of PP2C repression in a basal, ligand‐independent manner. During evolution, the constitutive ABA independent phosphatase binding activity of primitive land plants have achieved an ABA activated receptor protein.^[^ ^201^, ^202^ ^]^ The numbers of PYL family members have increased from mosses, to ferns, to angiosperms.^[^ ^202^ ^]^ This progression can be explained by the evolution of ABA signaling when the plants began to occupy terrestrial habitats. PYLs are conserved across species in the plant kingdom,^[^ ^202^ ^]^ including Arabidopsis, rice,^[^ ^203^, ^204^ ^]^ tomato,^[^ ^205^ ^]^ tobacco,^[^ ^206^ ^]^ soybean,^[^ ^207^ ^]^ and strawberry.^[^ ^208^ ^]^ Different subfamilies of this vast and unusually diverse receptor family^[^ ^202^ ^]^ have been reported based on sequence similarity, ABA sensitivity, oligomeric state, and basal activation level. However, the genetic redundancy of the ABA receptors complicates the investigation of the diverse roles of each homolog of this receptor family.^[^ ^209^ ^]^ Five research groups^[^ ^123^, ^185^, ^210^, ^211^ ^]^ have reported their work on the crystal structure of three of the 14‐member receptor family in *Arabidopsis*. The crystal structures had been solved for several PYLs as indicated in Table 3. Based on the study of over‐expressors of individual PYLs, responses of plant water‐use efficiency varies among PYLs.^[^ ^212^, ^213^ ^]^ The conserved structural features of PYLs contribute to efficient ligand–receptor interactions for ABA perception.^[^ ^202^ ^]^

**Table 3:** The crystal structures of PYLs

PYLs	Species	PP2C	Ligand	PDB ID
PYR1	Arabidopsis			3K3K^[^ ^123^ ^]^
PYR1	Arabidopsis		ABA	3K3K,^[^ ^123^ ^]^ 3K90^[^ ^310^ ^]^
PYR1	Arabidopsis		Pyrabactin	3NJO,^[^ ^311^ ^]^ 5UR4
PYR1	Arabidopsis		AS6	3WG8^[^ ^235^ ^]^
PYR1	Arabidopsis		Cyanabactin1	5UR5^[^ ^312^ ^]^
PYR1	Arabidopsis		PANMe	5YGV
PYR1	Arabidopsis		Cyanabactin	5UR6^[^ ^312^ ^]^
PYR1	*Festuca elata*		ABA	5UJV^[^ ^313^ ^]^
PYR1	Arabidopsis	HAB1	ABA	3QN1^[^ ^187^ ^]^
PYR1	Arabidopsis	HAB1	8t	5OR2^[^ ^314^ ^]^
PYR1	Arabidopsis	HAB1	10s	5OR6^[^ ^314^ ^]^
PYR1	Arabidopsis	HAB1	ABA	3ZVU^[^ ^315^ ^]^
PYR1	Arabidopsis	HAB1	Mandipropamid	4WVO^[^ ^232^ ^]^
PYL1	Arabidopsis			3KAY^[^ ^194^ ^]^
PYL1	Orange			5MMQ^[^ ^316^ ^]^
PYL1	Tomato			5MOA^[^ ^316^ ^]^
PYL1	Arabidopsis		ABA	3JRS^[^ ^317^ ^]^
PYL1	Arabidopsis		Pyrabactin	3NEF,^[^ ^217^ ^]^ 3NEG^[^ ^217^ ^]^
PYL1	Orange		ABA	5MMX^[^ ^316^ ^]^
PYL1	Tomato		ABA	5MOB^[^ ^316^ ^]^
PYL1	Arabidopsis	ABI1	ABA	3KDJ,^[^ ^211^ ^]^ 3JRQ^[^ ^317^ ^]^
PYL1	Arabidopsis	ABI1	Pyrabactin	3NMN^[^ ^318^ ^]^
PYL1	Orange	HAB1	ABA	5MN0^[^ ^316^ ^]^
PYL2	Arabidopsis			3KL1,^[^ ^214^ ^]^ 3KDH,^[^ ^211^ ^]^ 3KAZ^[^ ^194^ ^]^
PYL2	Arabidopsis		ABA	3KB0,^[^ ^319^ ^]^ 3KDI^[^ ^211^ ^]^
PYL2	Arabidopsis		Pyrabactin	3NS2,^[^ ^221^ ^]^ 3NR4,^[^ ^221^ ^]^ 3NJ0,^[^ ^311^ ^]^ 3NMH^[^ ^318^ ^]^
PYL2	Arabidopsis		Phaseic acid	5JNN^[^ ^266^ ^]^
PYL2	Arabidopsis		Pyrabactin	3NJ1^[^ ^311^ ^]^
PYL2	Arabidopsis		Pyrabactin	3NMP^[^ ^318^ ^]^
PYL2	Arabidopsis	HAB1	ABA	3KB3^[^ ^194^ ^]^
PYL2	Arabidopsis	HAB1	AM1	4LA7,^[^ ^188^ ^]^ 4LG5^[^ ^5^ ^]^
PYL2	Arabidopsis	HAB1	AM2	4LGA^[^ ^5^ ^]^
PYL2	Arabidopsis	HAB1	AM3	4LGB^[^ ^5^ ^]^
PYL2	Arabidopsis	HAB1	AMF1*α*	5VR7^[^ ^320^ ^]^
PYL2	Arabidopsis	HAB1	AMF1*β*	5VRO^[^ ^320^ ^]^
PYL2	Arabidopsis	HAB1	AMF2*α*	5VS5^[^ ^320^ ^]^
PYL2	Arabidopsis	HAB1	AMF2*β*	5VSQ^[^ ^320^ ^]^
PYL2	Arabidopsis	HAB1	AMF4	5VSR^[^ ^320^ ^]^
PYL2	Arabidopsis	HAB1	AMC1*β*	5VT7^[^ ^320^ ^]^
PYL2	Arabidopsis	ABI2	ABA	3UJL^[^ ^321^ ^]^
PYL2	Rice	PP2C06	ABA	4OIC^[^ ^322^ ^]^
PYL2	Arabidopsis	HAB1	Pyrabactin	3NMT^[^ ^318^ ^]^
PYL2	Arabidopsis	ABI2	Pyrabactin	3NMV^[^ ^318^ ^]^
PYL3	Arabidopsis			3KLX^[^ ^214^ ^]^
PYL3	Arabidopsis		ABA	4DSC,^[^ ^214^ ^]^ 4DSB^[^ ^214^ ^]^
PYL3	Arabidopsis		(−)‐ABA	4JDA
PYL3	Arabidopsis		Pyrabactin	3OJI^[^ ^214^ ^]^
PYL3	Arabidopsis	HAB1	ABA	5JO2,^[^ ^266^ ^]^ 4DS8^[^ ^214^ ^]^
PYL3	Arabidopsis	HAB1	Phaseic acid	5JO1^[^ ^266^ ^]^
PYL5	Arabidopsis			4JDL^[^ ^215^ ^]^
PYL9	Arabidopsis		ABA	3W9R,^[^ ^323^ ^]^ 3OQU^[^ ^215^ ^]^
PYL10	Arabidopsis			3RT2,^[^ ^187^ ^]^ 3UQH
PYL10	Arabidopsis		ABA	3R6P
PYL10	Arabidopsis	HAB1		3RT0^[^ ^187^ ^]^
PYL10	Rice	PP2C50	ABA	5GWP,^[^ ^324^ ^]^ 5GWO^[^ ^324^ ^]^
PYL10	Arabidopsis		Cyanabactin	6NWC^[^ ^325^ ^]^
PYL13	Arabidopsis	PP2CA		4N0G^[^ ^216^ ^]^

**Figure 8:**
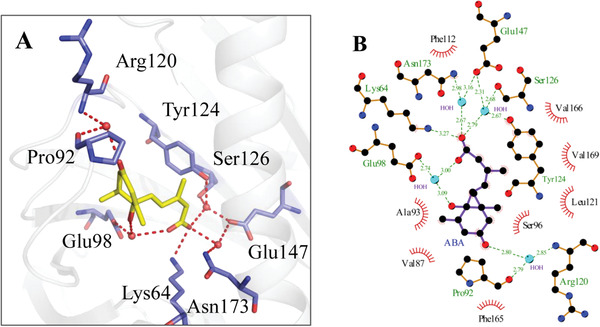
The binding mode of ABA and PYL2 (PDBID: 3KDI) in A) 3D and B) 2D (redrawn).^[^ ^211^ ^]^ In the 3D structure, the cartoon of PYL2 is colored in white (A). The important residues and ABA are shown in sticks with blue and yellow colors respectively. The H‐bonds are marked with red dotted lines.

Structural features of PYLs and ABA concerning the ligand–receptor interaction process are of vital importance in ABA signaling. A wealth of information is available on PYLs structure generated by crystallization studies of PYR1,^[^ ^123^ ^]^ PYL1, PYL2,^[^ ^211^, ^214^ ^]^ PYL3,^[^ ^214^, ^215^ ^]^ PYL5,^[^ ^215^ ^]^PYL9,^[^ ^214^ ^]^ PYL10,^[^ ^216^ ^]^ and PYL13.^[^ ^216^ ^]^ All PYLs are known to share a dominant helix‐grip structure. This characteristic motif consists of a seven‐stranded antiparallel *β*‐pleated sheet, which is flanked by two *α* helices. The *β*‐pleated sheets enfold a long carboxy‐terminal *α*‐helix of PYLs. The apo‐PYLs contain a sufficiently large hydrophobic pocket of 543˚A between the C‐terminal helix and *β* sheet. The size of this pocket is estimated to be 480 ˚A in the ABA‐bound state. The 23 pocket residues are highly conserved and are more hydrophobic than the other parts of PYLs. The L2/CL2/gate loop, which lies between *α*3 helix and *β*2 strand, and the L5/CL3/latch loop, which lies between the *β*5 and *β*6 strands, along with the C‐terminal *α* helix4, are essential in enclosing ABA in the ligand‐binding pocket. Insights into ABA structural features concerning PYL interaction (**Figure** **8**) explain the formation of the ionic bonds, hydrophobic interactions, and water‐mediated hydrogen bonds that stabilize ABA in the PYL active site. The structural features of “gate” and “loop” residues in the active sites are essential in ABA perception by PYLs.

Characterization of active site residues of PYLs provides a detailed understanding of the molecular events of the gate‐latch lock. These molecular events include the positioning of ABA inside the PYL active site and the conformational changes in the “gate” loop and “latch” loop. Bound ABA is fitted into the PYL active site with ABA's hydrocarbon chain and cyclohexenone ring interacting with the PYL pocket residues and surrounding water molecules. Three polar reactive groups of ABA (carboxyl, hydroxyl, and ketone) (Figure 8) form direct interactions or water‐mediated hydrogen bonds with the PYL. The interaction or bonds include a charged interaction/salt bridge between the K64 side chain and the ABA acid‐head; water‐mediated hydrogen bonds between the hydroxyl group of ABA and E98 and N173 residues; and the interactions of ketone group of ABA. Additional hydrogen bonds formed with other polar residues are involved in stabilizing ABA in the PYL active site.^[^ ^192^, ^217^ ^]^

A gate‐latch‐lock mechanism has been used to explain the ABA perception by PYLs and subsequent signal transduction events.^[^ ^192^ ^]^ The entrance to PYL active site is surrounded by both a “gate” like loop and a “latch” like region. The “gate” loop lies between the 3rd and 4th beta strands (in PYL2; residues 89–93: SGLPA), while the “latch is formed by a loop between the beta strands corresponding to 5th and 6th (residues 119–121: HRL in PYL2.) The position of latch remains mostly unchanged in apo and ABA‐bound forms. However, upon ABA binding, the SGLPA loop shifts 3–9 Å away from its apo‐position, which causes the ligand entrance to close and enclose the bound ABA molecule inside, preventing its contact with the outside solvent. There is another major conformational change in the latch motif, occurring at the residue E118, which immediately precedes HRL latch motif. In the ligand free form, the side chain of residue E118 points in to the active site, preventing the gate closure accompanied with SGLPA gate. In ABA bound state, the E118 side chain flips by ≈150° allowing sufficient space for the SGLPA to close on to the HRL latch. The space between the gate and latch is reduced from 11–14 to 3.5–3.7 Å. A new interface is formed with the gate and latch residues which fits into the active site of PP2C and facilitates subsequent steps in ABA signaling (**Table** **4**).^[^ ^192^ ^]^

**Table 4:** The currently known ABA receptors in Arabidopsis and corresponding interactional PP2Cs

PYLs	AGI annotation	Interacting PP2Cs
PYR1/RCAR11	AT4G17870	ABI1, ABI2, HAB1, HAB2, PP2CA
PYL1/RCAR12	AT5G46790	ABI1, ABI2, HAB1, HAB2, PP2CA
PYL2/RCAR14	AT2G26040	ABI1, HAB1, HAB2, PP2CA
PYL3/RCAR13	AT1G73000	ABI1, HAB1, HAB2, PP2CA
PYL4/RCAR10	AT2G38310	ABI1, HAB1, HAB2, PP2CA
PYL5/RCAR8	AT5G05440	ABI1, ABI2, HAB1, HAB2, PP2CA
PYL6/RCAR9	AT2G40330	ABI1, HAB1, HAB2, PP2CA
PYL7/RCAR2	AT4G01026	ABI1
PYL8/RCAR3	AT5G53160	ABI1, ABI2, HAB1, HAB2, PP2CA
PYL9/RCAR1	AT1G01360	ABI1, ABI2, HAB1, HAB2, PP2CA
PYL10/RCAR4	AT4G27920	ABI1, HAB1, HAB2, PP2CA
PYL11/RCAR5	AT5G45860	PP2CA/AHG3
PYL12/RCAR6	AT5G45870	PP2CA/AHG3
PYL13/RCAR7	AT4G18620	PP2CA, AHG3, ABI1, ABI2

#### 6.2.2 Chemical Manipulation of PYLs

Chemical manipulation of PYLs may be termed as the use of a small molecule that is functionally capable of binding and activating/inactivating (agonists/antagonists) PYLs. The small molecule is essentially a ligand, and the possible ligand–receptor interactions may be agonistic or antagonistic. Agonists of receptor proteins would be ligands that bind and activate receptors and bring about a biological response. Antagonists would be ligands that bind to but fail to activate receptors, i.e., that fail to induce a biological response upon binding. An antagonist could also block or weaken responses following its binding. The concept of developing agonists to ABA binding emerged as researchers recognized that the instability of ABA limited its use as an agrochemical. ABA antagonists could also increase our understanding of ABA signaling in vivo, especially for plants whose genomes have not been well characterized. As discussed in the next section, chemical manipulation of PYLs has great potential for directing ABA signaling according to a specific agrochemical requirement.

#### 6.2.3 Progress in PYLs Chemical Manipulation

The PYLs are the best known and most exploited protein for the chemical manipulation of ABA signaling to date. The strategies of small molecule design as ABA analogs vary froma rational design approach to screening. The early attempts in ABA analog design resulted in stereoisomers and geometrical isomers of ABA, such as the (R)–ABA enantiomer.^[^ ^218^ ^]^ Several studies reported that the (S)‐ and (R)‐stereoisomers of ABA have different binding patterns to the active sites of PYLs.^[^ ^14^, ^30^, ^215^, ^219^ ^]^ However, the stereoisomeric approach has not experienced considerable development over the recent years. More recent and more successful attempts have focused on chemical genetic screens and more‐specific structure modifications of small molecules. The sulfonamide pyrabactin, the first synthetic ABA mimic, was a selective ABA agonist to PYR1 and PYL1. Pyrabactin demonstrated seed selectivity, which limited its applications. Another sulfonamide compound quinabactin which was identified through a small molecule compound library screen remains a successful ABA mimic with biological responses superior to those of ABA. The next significant efforts on the design of ABA mimics exploited the structure of quinabactin, improving the hydrogen‐bonding network, and minimizing the ring‐structure‐occupying space inside the PYL active site. This rational design concept gave rise to the small molecule cyanabactin, which is highly selective to PYLs of subfamily I and II. Cyanabactin has superior ABA‐like activity in vegetative tissues even though it has no relation to ABA structure. The next generation of ABA mimics targeted PYL subfamilies.

The combination of a virtual screening approach and the merging of parts of different scaffolds that enhance receptor‐ligand interaction resulted in the development of opabactin. The biological activity of opabactin is superior to that of ABA and of other reported ABA agonists because of its salt bridge formation with conserved lysine on PYL and stabilized Trp lock. Another parallel approach in agonist development was to stabilize the ABA inside the PYL by structurally modifying the ABA 3ʹ carbon with alkyl chains of different lengths. Members of these alkyl‐sulfonyl ABA molecules (ASn; *n* = alkyl chain length), however, were found to be both agonists and antagonists, depending on their side‐chain length. The findings of this initial approach revealed that the PP2C/PYR1 interaction could be sterically hindered to varying degrees by varying the lengths of the 3’ alkyl chain. Guided by these findings, researchers modified the C3’ and C4’ of ABA; this resulted in +PAO4 and PANme compounds, respectively, which sterically hindered functional PP2C interactions. As antagonists, both +PAO4 and PANme stabilized a closed‐gate conformation, while tetralone ABA analogs antagonized ABA action with an open‐gate conformation of PYLs. Regarding PYL antagonists, AA1 was the first complete ABA PAN antagonist that targeted all PYLs in Arabidopsis, rice, and tomato.

In addition to the “wild‐type PYL‐designed ligand structure” strategy, a mutation approach was able to produce PYR1.^MANDI^ Alteration of the active site of PYR1 conferred nanomolar sensitivity to the agrochemical mandipropamid. The orthogonal receptor‐ligand interactions can be considered a parallel approach in PYLs chemical manipulation. More recently, the chemical manipulation of PYLs has mainly targeted on the development of molecules that are superior to ABA in terms of its stress‐protective responses.

It is apparent that the recent structural and biochemical information on crop PYLs will drive the chemical manipulation approaches in to new directions. The initially accepted sequential model describes a series of coupled events of ABA‐PYL binding and gate‐latch closure to activate the PYL, where the structure of ABA‐bound PYL is hardly modified by PP2Cs. The recently updated conformation selection model of ABA binding explains independent events of ABA‐PYL dimeric complex formation and ABA‐PYL‐PP2C ternary complex, where PP2Cs preferentially select the gate closed‐latch closed conformation of PYLs to form a high affinity complex.^[^ ^27^ ^]^ The possibility that the above complexes could be studied separately, (maybe aiming to alter the equilibrium of the gate closed‐latch open and gate closed‐latch closed states) and the evidence on PP2Cs to be potential targets, point at new directions of chemical manipulation. On the other hand, the critical gain of function mutation hab1^W385A^ gave rise to un‐impaired basal PP2C activity yet abolished ABA‐dependent PYL mediated PP2C inhibition, and to strong, dominant ABA insensitivity in vivo.^[^ ^220^ ^]^ As the Trp lock impaired PP2Cs block the progression of ABA perception, there exist a target point for the chemical modulator activity. The significant chemical modulators of PYLs are listed in Table 5 and are discussed in detail in the following section.

**Table 5:** Small molecules for chemical manipulation of ABA receptors

Chemical modulator		Activity	Design strategy	Significance	Reference
Pyrabactin	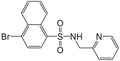	Selective agonist	Chemical genetics screen	First synthetic ABA agonist Chemical control of seed germination	^[^ ^188^ ^]^
Quinabactin	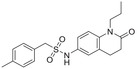	Selective agonist	Compound library screen	A highly potential ABA agonist for chemical control of plant water use	^[^ ^186^ ^]^
AMF4	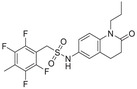	Selective agonist	Structure modification	A highly potential ABA agonist for controlling plant water use, with more persistent ABA‐like activity	^[^ ^224^ ^]^
Cyanabactin	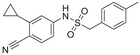	Selective agonist	Rational design	A rationally designed, highly effective ABA agonist to control plant water use	^[^ ^228^ ^]^
Mandipropamid	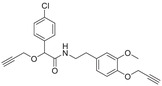	Agonist of PYR1^MANDI^	Existing Agrochemical, combined with saturation mutagenesis	An orthogonal ABA agonist utilizing the chemical genetic approach Chemical control of germination and water use	^[^ ^326^ ^]^
Opabactin	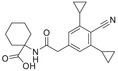	Selective agonist	Virtual screen and scaffold merging	Targets conserved lysine in subclass II/III PYLs; nonsulfonamide ABA pan agonist against Arabidopsis, tomato, and wheat	^[^ ^325^ ^]^
AS6	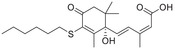	Antagonist	Structural modification	Block PYL‐PP2C interaction, stabilize closed gate conformation	^[^ ^327^ ^]^
PANme	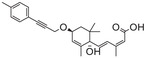	Antagonist	Structural modification	Block PYL‐PP2C interaction	^[^ ^237^ ^]^
PAO4	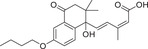	Antagonist	Structural modification	Conformationally restricted ABA analog with respect to side chain length and size	^[^ ^327^ ^]^
PBI686	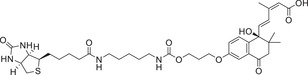	Antagonist	Structural modification	Agonist‐antagonist conversion by modifying tetralone moiety; alters PP2C interaction	^[^ ^328^ ^]^
AA1	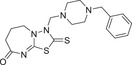	PAN‐antagonist/broad spectrum antagonist	Chemical genetics screen	The first broad spectrum ABA antagonist, chemical manipulation of plant senescence	^[^ ^18^ ^]^

##### PYLs Chemical Manipulation by Pyrabactin and Pyrabactin‐Based Structures

Pyrabactin: Pyrabactin was the first synthetic naphthalene sulfonamide ABA agonist, was identified by chemical genetic screen based on seed germination inhibitory activity. Pyrabactin is a selective ABA agonist with partial antagonist activity. It may strongly activate PYR1, which is highly expressed in seeds, as well as PYL1, which is structurally similar to PYR1. The studies on PYR1 and pyrabactin have mostly helped to elucidate the function of PYR1 as an ABA receptor in *Arabidopsis*.^[^ ^14^, ^221^ ^]^ The studies on PYR1 and pyrabactin have mostly helped to elucidate the function of PYR1 as an ABA receptor in *Arabidopsis*.^[^ ^14^, ^221^ ^]^ Studies on pyrabactin‐bound PYL1 and PYL2 revealed that pyrabactin in PYL2 is rotated by 90 degrees compared with pyrabactin in PYL1, resulting in two different orientations.^[^ ^221^, ^222^ ^]^ Therefore, pyrabactin was selective to PYL1 but not to PYL2, even in the receptor‐bound confirmation. A single amino acid alteration from valine of PYL1to isoleucine of PYL2 may explain why the orientation of the molecule differed in ligand‐binding pockets of PYL1 and PYL2. The unproductive orientation in PYL2 prevents the receptor from closing the gate. Several variants of pyrabactin have been synthesized. Researchers have replaced or changed the position of nitrogen on the pyridyl ring, replaced the naphthyl ring into a naphtholactam, and replaced the pyridyl methylamino group with a methionine derivative.^[^ ^12^ ^]^ Interestingly, pyrabactin also binds to PYL3 in a distinct, nonproductive manner, yet with gate closure. The above‐compact trans homodimer of PYL3 binds pyrabactin to decrease the PP2C binding affinity. Therefore, pyrabactin works as an antagonist for PYL3 by way of a unique mechanism. According to several studies, it remains unclear whether the closed state of the gate responding to pyrabactin is a sole determinant of whether molecules function as agonists/antagonists for ABA receptors.^[^ ^192^, ^217^, ^221^, ^223^ ^]^ The above information supports the selective nature of pyrabactin as an agonist. Because pyrabactin is a seed‐selective ABA agonist, its ABA‐like effects are less evident in vegetative parts than in seeds. Even though pyrabactin is unable to activate all of the ABA receptors, the mechanism of its ABA antagonism has provided valuable information, and pyrabactin‐based agonists have also been patented (USPTO application 20160280651^[^ ^224^ ^]^).

ABA mimic 1 (AM1)/quinabactin: AM1 remains an important ABA agonist developed to date. The sulfonamide‐based ABA analog ABA mimic 1 (AM1)/quinabactin was developed along with its structural derivatives, AM2 and AM3. A yeast two‐hybrid screen of about 57 000 compounds was used to identify AM1.^[^ ^186^ ^]^ AM1 was the second sulfonamide selective agonist, which was similar to pyrabactin. The dihydroquinolinone ring of AM1 rotates upon interaction with PYL, and this allows deeper positioning of the sulfonamide linkage and the 4‐benzylmethyl substructure in the PYL active site, which results in stronger binding.^[^ ^225^ ^]^ AM1 was many times more potent in promoting PYLs/PP2C interaction than ABA or pyrabactin. The ABA receptor PYR1 was effectively activated by quinabactin/AM1.^[^ ^5^ ^]^ The phenotypic effects of AM1 indicated that it is a potent ABA agonist. In addition to activating almost all of the Arabidopsis PYLs, AM1 activated a gene network that was similar to that activated by ABA. Six ABA receptor subtypes (PYR1, PYL1, 2, 3, 5, and 7) binding with AM1 inhibited HAB1. The induction of gene expression is almost identical for AM1 and ABA AM1 treatments inhibited seed germination (at 1 × 10^−6^ m) and reduced water loss and enhanced drought resistance (at 50 × 10^−6^ m) in wild‐type Col (0) Arabidopsis.^[^ ^5^ ^]^ Drought‐resistant responses were observed in soybean, tomato, maize, and Arabidopsis following AM1 treatment. The potential of AM1 for increasing drought tolerance was also reported in oilseed rape^[^ ^226^ ^]^ and bermuda grass.^[^ ^227^ ^]^ Although AM1 is unable to activate the full complement of ABA receptors, it can control guard cell responses and other responses in vegetative tissues. These results suggest that replacements of ABA are not limited to PAN‐agonists.^[^ ^12^ ^]^

AM1 fluorine derivatives (AMFs): AMFs are improved structural analogs of AM1 that have more desirable phenotypic effects. In these analogs, the structure of AM1 is modified to increase polarity and to thereby increase the formation of hydrogen bonds between the ligand and receptor. AMF4 had better activity than its preceding compound, AM1. In developing AMFs, the structure of AM1 was further improved by progressively substituting fluorine atoms on the benzyl ring. The increase in electro‐negativity caused by fluorine atoms led to an increase in the number of hydrogen bonds with the PYL and therefore to an increase in the stability of receptor activation. The fluorine atoms also increase the lipo‐solubility of the compounds and result in increased plasma membrane permeability. In this modified structure, the fluoro‐benzyl group, sulfonamide link, and di‐hydro quinolinone ring of AMF fit into the active site of the PYL2.

Apparently, because of the changes described in the previous paragraph, AMF4 was proved to be a better agonist than AM1 or ABA. In a transcriptomic profile of 3‐week‐old Arabidopsis seedlings, ABA‐ and stress‐responsive genes (*MYB60, RD26, HAI1, RD28, RD29b, RAB18, LEA4‐5, P5CS1, NCED3, SnRK3.10*, and *MPK3*) remained highly induced 3 days after treatment with AMF4 but not after treatment with ABA. AMF4 was more stable in plants than AM1 or ABA at 12 h post‐treatment, where AMF4 was the most effective. In the presence of ABA, a negative feedback loop of transcription down‐regulates PYL expression and up‐regulates PP2C expression. The negative feedback counteracts the increase of PYL–PP2C interactions during signaling. Interestingly, this phenomenon was also detected with AMFs, which down‐regulated PYL2 gene expression. Accordingly, AMF4 was superior to previous ABA analogs.^[^ ^224^ ^]^

Cyanabactin: Cyanabactin was designed specifically focusing on the requirements for ligand–PYL interaction. Aiming at developing a small molecule with better selectivity and a higher ability to reduce transpirational water loss, cyanabactin was reported in 2017.^[^ ^228^ ^]^ Cyanabactin was developed by further modifying the sulfonamide‐based ABA mimic quinabactin/AM1. AM1 was successful in causing ABA‐like effects in multiple vegetative tissues and activating multiple ABA receptors in subfamily ΙΙΙ. Researchers, therefore, hypothesized that simplifying the AM1 structure by reducing the quinolinone ring and by increasing polar groups would be sufficient to activate PYLs.^[^ ^228^ ^]^ Cyanabactin was the first reported ABA mimic that lacks a core ABA skeleton. Although quinabactin's sulfonamide linkage and 4‐methylbenzyl substructure are present in cyanabactin, the complexity of the bulky dihydroquinolinone ring is reduced. The arylnitrile of cyanabactin mimics ABA's cyclohexenone oxygen and facilitates water‐mediated H‐bonding in the tryptophan lock, which is critical in stable receptor activation. It was hypothesized that the relatively simple and less bulky structure of cyanabactin increases its solubility. Furthermore, ABA's carboxylate and C6 methyl groups are mimicked by sulfonamide and 4‐methylbenzyl groups, respectively, in cyanabactin.

Cyanabactin is superior to pyrabactin and quinabactin in activating subfamily III ABA receptors. It is potent and selective at the nanomolar level to activate PYR1. Cyanabactin induces many ABA effects, such as seed germination inhibition, gene expression regulation, and reduced transpiration. Cyanabactin reduced whole‐plant stomatal conductance in Arabidopsis.^[^ ^228^ ^]^ As a consequence, research on cyanabactin initiated chemical manipulation that targeted PYL subfamilies.

##### PYLs Chemical Manipulation Guided by Virtual Screening and Scaffold Grafting

Opabactin: Opabactin (OP: over powered for ABA activation)^[^ ^229^ ^]^ is the most recent and most potent ABA agonist developed. The authors hypothesized that the lack of interaction between current sulfonamide based PYL agonists and a conserved lysine in the PYLs results in incomplete activation of PYLs, leading to short‐lived bioactivity. The phenyl acetamido‐cyclohexane carboxylic acid scaffold interacting with conserved lysine of PYR1 was identified based on the ZINC database through virtual screening. The scaffold was improved by grafting the 4‐cyano‐3‐cyclopropylphenyl headgroup of cyanabactin.^[^ ^228^ ^]^ A structural derivative that formed a salt bridge to K59 and strong Trp lock interactions (essential in PYL activation) was named opabactin. Opabactin activates subfamily III receptors PYR1, PYL1, and PYL2. Opabactin protects wheat plants from drought and caused long‐lasting increases in leaf temperatures in wheat and tomato as indicated by infrared thermal imaging. Opabactin inhibition of Arabidopsis seed germination was about 10‐fold greater than that of ABA. This small molecule has an ≈7‐fold improved *K*_d_ value compared to ABA. The lysine salt bridge and facilitated Trp lock interactions are unique to opabactin, i.e., they do not occur in the other sulfonamide‐based ABA agonists quinabactin, pyrabactin, cyanabactin, and AMF4. These features make opabactin compound a useful tool for chemical manipulation of plant water use.^[^ ^229^ ^]^

##### Chemical–Genetic Manipulation of PYLs by Mandipropamid

The antifungal compound mandipropamid has been used to manipulate ABA signaling so as to increase plant survival during water deficit conditions. The introduced mutations F108A and F159L in the engineered PYR1 receptor resulted in an increased volume of the active site and facilitated the placement of the long propargyl substituents of mandipropamid in the ligand‐binding pocket. The hydrophobic interactions between mandipropamid and S109, and mandipropamid and G392 of HAB1 were favored by F108A and F159L.^[^ ^230^ ^]^ The above features in the mutant PYR1 receptor were the most distinct. Mandipropamid interacts with mutated pocket residues of the PYR1 ABA receptor and initiates ABA‐like phenotypic effects. The engineered PYR1 receptor PYR1^MANDI^ showed ABA‐like responses in transgenic PYR1^MANDI^ transgenic Arabidopsis and Tomato treated with Mandipropamid was able to show ABA like responses. Mandipropamid induced stomatal closure and inhibited seed germination in transgenic plants with modified PYR1.^[^ ^230^, ^231^ ^]^

##### Chemical Manipulation of PYL by Structural Modifications of ABA

The natural ABA structure has also been altered to obtain ABA analogs. This includes the removal of certain structural groups from 6ʹ and 7ʹ carbon atoms and modifying the groups attached to 3ʹ and 4ʹcarbon atoms of natural ABA (Figure 1). This approach generated a number of ABA antagonists with increased levels of antagonist activity. This approach also provided insight into the inhibition of PYL/PP2C interactions, an area that has potential for the development of ABA antagonists. In addition, 8‐hydroxylation‐resistant ABA derivatives^[^ ^232^, ^233^ ^]^ have been developed but have not been fully evaluated or further improved.

6‐nor ABA and 7ʹ‐nor ABA: 6‐nor ABA and 7‐nor ABA are direct structural modifications of ABA, where the C6 or C7 methyl groups are absent from the basic ABA structure.^[^ ^218^ ^]^ They were developed as PYLs‐selective agonists. Dimeric and monomeric receptor amino acid residues differ in single amino acid.^[^ ^217^, ^225^ ^]^ Only one residue valine (Val), which is present in dimeric receptors, is replaced by leucine (Leu) (PYL7‐10) or isoleucine (Ile) (PYL4‐6, PYL11, and PYL12) in monomeric receptors. This residue position was thought to form hydrophobic interactions with the C6 and/or C7 methyl group of the ABA molecule. Considering the steric bulkiness of each residue, researchers predicted that hydrophobic interactions could be induced most easily by Leu, followed by Ile and then Val. With this reasoning, the authors further predicted that, even in the absence of the C6 and/or C7 methyl groups, gate closure of monomeric receptors would occur. Thus, the methyl groups at C6 of the basic ABA structure were removed in 6‐nor ABA, and those at C7 were removed in 7‐nor ABA. Both analogs, however, had reduced agonist activities in the PP2C assay but were found to be selective agonists in inducing stomatal closure. It was noted that that 6‐nor ABA could act as an agonist of PYL5 and/or PYL6.^[^ ^234^ ^]^

AS6: The ABA antagonist AS6 blocks PYL/PP2C interactions by steric hindrance and blocks stress‐induced ABA responses in vivo. AS6 belongs to the AS*n* series (3ʹalkylsulfonyl‐ABA, where *n* denotes the alkyl chain length; *n* = 2–12). The basic ABA structure was modified at C3. The members of this series exhibited varying degrees of antagonist activity in terms of forming stable PYL/PP2C interactions.^[^ ^235^ ^]^ Details of the PYL–PP2C interaction supported the development of the ASn series. Crystal structure studies have revealed that five highly conserved hydrophobic amino acid residues in PYLs (Phe61, Leu87, Pro88, Phe159, and Val163 of PYR1) form tunnels near 3ʹC—H and 4ʹC=O of ABA. The formation of these tunnels accompanies gate closure, and the entrance to the tunnels is on the PP2C connecting interface. This position has been considered a possible site for ABA structure modifications. A computational model supported the validity of this design because the highly conserved PP2C residue (HAB1; Val393) occupies this position under natural conditions. Researchers predicted that PYL/PP2C interactions would be blocked by ABA analogs having sufficiently long 3′ alkyl chains that result in an antagonistic activity. The AS*n* series that was subsequently developed is based on 3ʹalkylsulfonyl‐ABA, where *n* denotes the alkyl chain length.

AS6 proved to be a favorable antagonist both in vitro and in vivo condition. Its S‐hexyl chain is positioned in a gate‐closed conformation, presenting a steric hindrance to PP2C binding. The PP2C inhibition of AS6 was <50%, suggesting weak, partial agonist activity. AS6 also did not enable full recovery of PP2C activity even when added at a level 20‐fold greater than ABA. In in vivo studies, AS6 antagonized ABA effects in Arabidopsis, lettuce, and radish in terms of germination, seedling growth, water loss, and temperature. AS6 repressed stress‐induced ABA‐responsive gene expression in response to mannitol treatments.^[^ ^235^ ^]^ Based on these results, the steric strategy of controlling protein–protein interactions during ABA signaling was considered useful for developing useful agonists or antagonists.

+PAO4 (propenyl‐ABA with an *O*‐butyl chain): A further fine‐tuning of the compound AS6 resulted in the compound +PAO4. +PAO4 is a propenyl‐ABA with an *O*‐butyl chain and is more potent than AS6 in terms of subclass‐specific antagonist activity. +PAO4 is conformationally restricted in its side chain. Researchers noted that the 3ʹ alkyl chains of an AS6‐derived ABA antagonist should be both sufficiently long and thin to insert into the 3ʹ tunnel. This requirement still allowed conformational changes in the gate closure of PYLs. Therefore, the +PAO4 compound is a conformationally restricted analog of AS6. +PAO4 has increased affinity toward the PYL proteins PYR1 and PYL1–PYL6 (members of subfamily I and II) and is more antagonistic in vivo than AS6. The reduced entropy penalty of +PAO4 by binding PYLs may be attributed to the increased PYL affinity. PYL8 and PYL10 (subfamily III), however, were not activated. +PAO4 binds more strongly to PYL5 than AS6. The compound +PAO4 showed antagonistic activity in inhibiting seed germination and root growth. The antagonistic activity of +PAO4 stronger than AS6 as observed in Arabidopsis and lettuce. The conformational restriction strategy, which has been applied in drug design, has been successfully used in developing +PAO4. +PAO4 also shows a plant species‐dependent variation in its effects as an ABA antagonist, which reflects the variation among plants in PYLs.^[^ ^236^ ^]^

PANMe: PANMe is a 4ʹ‐modified ABA analog, superior to AS6 in its antagonism and without intrinsic agonist activity. PANMe relieved ABA‐induced phenotypic effects in several plant systems, suggesting a possible application as an agrochemical to obtain stable agricultural production.^[^ ^237^ ^]^ As per the gate‐latch‐lock mechanism, ABA‐bound PYLs are stabilized in a gate‐closed conformation to interact with PP2Cs. This interaction forms the PYL–ABA–PP2C complex, whose formation and stabilization requires a conserved Trp residue in PP2Cs. The indole ring of Trp is inserted into a tunnel adjacent to the C4′ of ABA in the PYL‐ABA complex. To prevent the Trp insertion, researchers synthesized 4′‐*O*‐phenylpropynyl ABA analogs (PANs) as novel PYL antagonists. Of the five PANs, PANMe proved superior to AS6 in the activity.^[^ ^237^ ^]^

PANMe has several physiological effects that could be useful in agriculture. A 3‐fold higher concentration of PANMe than ABA could restore the ABA‐delayed germination time of Arabidopsis seeds. This effect, in contrast, could not be obtained even with an AS6 concentration 100‐fold greater than that of ABA. PANMe at 50 × 10^−6^ m concentration could revert ABA‐induced stomatal closure by 90%. AS6 could achieve this effect only at 100 × 10^−6^ m. The temperature‐induced germination delay of Arabidopsis seeds could be relieved by PANMe at concentrations higher than 0.3 m, and once again, the effect of PANMe was stronger than that of AS6. PANMe is an ABA antagonist of germination in lettuce, leaf celery, mitsuba, perilla, and komatsuna. However, PANMe was unable to relieve the inhibitory effects of exogenous ABA on seedling root elongation in rice, carrot, or proso millet. PANMe did not induce the expression of the ABA‐responsive genes *GBF3*, *MAPKKK18*, *RD29A*, or *RD29B*. Because PANMe effects have been observed in a broad range of plant species, it could be a useful tool for studying ABA signaling in plants.^[^ ^237^ ^]^

##### PYLs Chemical Manipulation by Tetralone Analogs

Tetralone analogs were initially developed as ABA agonists.^[^ ^238^, ^239^ ^]^ The basic tetralone structure is bicyclic, with fused aromatic and cyclohexane rings and ketone groups. The potential agonist and antagonist‐like tetralone structures were identified based on an in vitro screen of PP2C enzyme activity. At present, reported tetralone analogs such as PBI686 are ABA antagonists.^[^ ^240^ ^]^

The position of the tetralone ring positioned toward the gate opening may enable PBI686 to participate in strong Van der Waals interactions with Val and Leu in the gate and latch loops and participate in weaker interactions with Phe and Val in the terminal helix. The tether of PBI686, which protrudes from the ligand‐binding cavity, determines the degree of antagonism/selectivity of the analog.

##### Chemical Manipulation of PYLs by the Antagonist AA1

The first broad‐spectrum ABA antagonist developed was ABA antagonist 1 (AA1).^[^ ^18^, ^19^ ^]^ A high throughput chemical genetic screen was performed to reveal this small molecule's ability to relieve ABA inhibition of seed germination AA1 was screened out from about 12000 novel structures as a ligand for PYLs and was subsequently found to have no intrinsic agonist activity.

AA1 antagonism is facilitated by its structure. AA1 demonstrated ABA antagonistic activity both in vitro and in vivo. The diazepin‐8‐one group core structure of AA1, even without any side chain, can dock in the ligand‐binding pocket of ABA receptors (as observed in PYL2). Hydrogen bonds formed by N, O, and S atoms are responsible for its antagonistic activity. AA1 also binds to both dimeric and monomeric receptors, which explains its broad‐spectrum of antagonistic activity

AA1 targeted all 13 of the Arabidopsis ABA receptors. AA1 can delay fruit ripening in tomato, delay senescence in rice, and inhibit chlorophyll breakdown in leaves. These responses, however, are not connected to the senescence‐delaying activity of cytokinin. Because it is able to act on all known PYLs/PP2C interactions, and because of its PAN‐antagonist nature, AA1 outperforms AS6 and tetralone structures in its antagonism. Because it can delay senescence, AA1 has great potential as an agrochemical,^[^ ^18^ ^]^ and was used in the first reported attempt to chemically manipulate plant senescence.^[^ ^19^ ^]^ Because its structure is simple, AA1 is inexpensive to synthesize. Accordingly, additional applications of AA1 as an ABA are likely to be developed.

### 6.3 SNF1‐Related Protein Kinases (SnRK2s)

Plant SnRK2s vary considerably in their stress response functions. The SnRK2 family of proteins can be divided into the following subsets SnRK1s, SnRK2s, and SnRK3s based on sequence similarity and domain structure.^[^ ^241^ ^]^ The SnRK2 subset in Arabidopsis consists of 10 protein kinases^[^ ^241^ ^]^ and appears to be unique to plants.^[^ ^242^ ^]^ The SnRK2 family can also be divided into subclasses I, II, and III based on phylogeny.^[^ ^243^ ^]^ In Arabidopsis, members of subclass I (SnRK2.1, SnRK2.4, SnRK2.5, and SnRK2.10) are not responsive to ABA. Members of subclass II, SnRK2.7 , and SnRK2.8 are poorly or not activated by ABA. Meanwhile, members of subclass III, SnRK2.2, SnRK2.3, and SnRK2.6, as positive central regulators, are strongly activated in the presence of ABA.^[^ ^243^, ^244^ ^] [^ ^69^ ^]^ As determined by the reverse genetics approach, SnRK2.2 and SnRK2.3 are the protein kinases that positively regulate ABA signaling in seed germination. SnRK2.2 and SnRK2.3 also have highly redundant functions in AB‐induced seed dormancy, ABA inhibition of seedling growth proline accumulation, etc.^[^ ^245^ ^]^ SnRK2.6 and ABI1, which directly interact through domain II (also known as the ABA box), control stomatal closure.^[^ ^246^ ^]^ SnRK2.6 interacts directly with the SLAC1 channel and enhances its activity by phosphorylation. Members of SnRK2 subclass III are vital for ABA‐mediated stress signaling and may be possible targets for chemical manipulation.

SnRK2s are protein kinases that phosphorylate downstream components of ABA signaling. The ABA signaling pathway is controlled by both SnRK2s and group‐A PP2Cs. After binding with ABA, the PYLs‐ABA complex inhibits the activity of PP2C. Thus, the kinase activity of SnRK2.6 is released to activate the downstream factors and to then affect downstream physiological activities. In the absence of ABA, in contrast, PP2C inhibits the activity of SnRK2.6. Therefore, SnRK2s are a critical component in the core ABA signaling.

**Figure 9:**
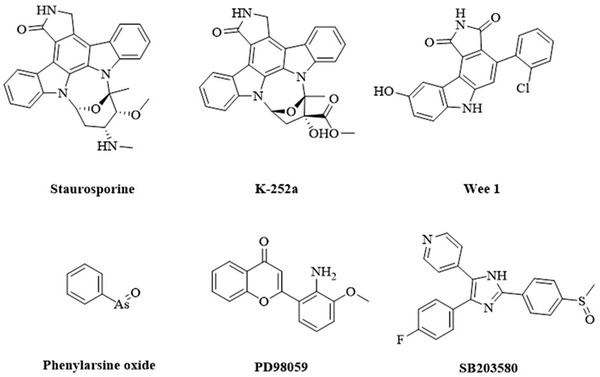
The compounds experimented in chemical manipulation on SnRK2 proteins.

There is little information regarding the chemical modulation of SnRK2 proteins in plants. In mammals, in contrast, SnRK proteins have been well‐studied. The compounds studied as inhibitors of plant SnRK2 proteins are summarized in **Figure** **9**. In addition, a range of small molecules that inhibit protein kinases in mammals are available. These were designed according to pharmacological requirements, and 28 have been FDA approved.^[^ ^247^ ^]^ Research is needed to determine whether small molecules that inhibit protein kinases in mammals also inhibit protein kinases in plants.^[^ ^247^ ^]^

#### 6.3.1 Indirect Chemical Manipulation of SnRK2

##### K‐252a

More than a decade before the ABA core signaling events were decoded, a chemical‐biological study conducted in 1997 revealed that the protein kinase inhibitor K‐252a inhibited ABA‐mediated actions in guard cells. K‐252a was also found to inhibit SnRK2.2, SnRK2.3, and SnRK2.6 in vitro. ABA‐impaired activation of slow anion channels in guard cells and ABA‐induced stomatal closure were partially rescued by K‐252a in Arabidopsis *abi1* but not in *abi2*. This effect was evident at high concentrations of ABA (as high as 50 × 10^−6^ and 100 × 10^−6^ m) and at inhibitor concentrations as low as 1 × 10^−6^ m. The activation of anion channels in mutant guard cells was inhibited by the phosphatase inhibitor okadaic acid. The use of inhibitors also increased the understanding of the relative positions and roles of ABA signaling components.^[^ ^248^ ^]^ K252a was found to rapidly activate HOSAK, a 40‐kD high osmotic stress‐activated kinase, in *Nicotiana tabacum* cells. HOSAK was regarded as a component of a Ca^2+^‐ and ABA‐independent pathway involved with plant adaptation to hyperosmotic stress.^[^ ^249^ ^]^ In an attempt to study protein–protein interactions in ABA signal transduction, researchers identified the novel SnRK2.6 inhibitors staurosporine and Wee1. The corresponding screen comprised ≈300 commercially available kinase inhibitors. The small molecules for kinase inhibitor screen were obtained from four kinase inhibitor libraries (Calbiochem I, II, and III, and the Tocriscreen inhibitor toolbox) and were used at 50 × 10^−6^ m concentrations. Three closely related SnRK2 proteins (SnRK2.2, SnRK2.3, and SnRK2.6) were used in an in vitro thermal stability shift assay (SnRK2.2 and SnRK2.3 regulate seed germination and root growth, and SnRK2.6 directly regulates stomatal response). In that assay, staurosporine, a staurosporine analog K252a, and Wee1 strongly inhibited SnRK2.2 and SnRK2.3 and inhibited SnRK2.6 to a lower but acceptable level. Another study focused on a 42 kD protein kinase, Nicotiana tabacum osmotic stress‐activated protein kinase (NtOSAK), which is rapidly activated under hyperosmotic stress. The protein was assigned to the SNF1‐related protein kinase type 2 (SnRK2) family and is a Ca^2+^‐independent Ser/Thr protein kinase. An array of protein kinase inhibitors were tested on NtOSAK, including heparin, 1‐(*β*‐d‐ribofuranosyl)‐5,6‐dichlorobenzimidazole, and tetrabromoimidazole (potent inhibitors of CK2); bisindolylmaleimide, Ro 318220, and W‐7 (inhibitors of Ca^2+^‐dependent protein kinases); SB 203580 (an inhibitor of mammalian p38 mitogen‐activated protein kinase [MAPK]); PD98059 and LY294002 (inhibitors of MAPK kinases); H‐89 (developed as a selective PKA inhibitor but is also an inhibitor of other kinases including AMPK); quercetin (an inhibitor of several protein kinases including CK2, S6K1, GSK3, and AMPK); and staurosporine (a nonspecific inhibitor of many Ser/Thr protein kinases). However, only the nonspecific inhibitor staurosporine inhibited NtOSAK activity with an IC_50_ value of 7 nm. ^[^ ^250^ ^]^

#### 6.3.2 Indirect Chemical Manipulation of SnRK2

##### ABA‐Dependent MAPK Signaling Inhibitors

MAPKs are signaling cascades that transmit environmental and developmental signals. A MAPK cascade consists of three types of protein kinases, which can be reversibly phosphorylated. The sequential phosphorylation events lead to further substrate phosphorylation that alters the activity of SnRK2s and brings about changes in gene expression. Thus, ABA‐dependent MAPK signaling events may be useful targets for indirect manipulation of SnRK2s.

MAPK substrates are associated with the regulation of plant development and stress responses. The substrates in MAPK signaling include transcription factors, phospholipases, cytoskeletal proteins, and microtubule‐associated proteins. In response to a variety of stimuli, MAPK‐based phosphorylation of these substrates occurs and results in the expression of specific sets of genes.^[^ ^251^ ^]^ Overall, MAPK cascades can relay and amplify signals as well as regulate processes. The processes under MAPK control include innate immune responses, plant development (the cell cycle and cell growth, differentiation, and death), stress responses (to drought, salinity, cold, wounding, ozone, and ROS), and hormonal responses.^[^ ^252^ ^]^

Inhibitors of MAPK. Pharmacological experiments using MAPK inhibitors have increased our understanding of the function of MAPK signaling in ABA‐related signaling cascades.^[^ ^251^ ^]^ In barley aleurone protoplasts, the tyrosine phosphatase inhibitor phenylarsine oxide completely blocked ABA‐induced MAP kinase activation and RAB16 gene expression.^[^ ^253^ ^]^ The epidermal peels isolated from *Pisum sativum* L. leaves, a 45 kDa protein was detected with the characteristics of MAPK, which may be activated by ABA. The compound PD98059 (an inhibitor of MAPK kinase) inhibited the kinase activity of the above 45 kDa protein. The ABA‐ induced stomata closing of *P. sativum* L. leaf epidermal peels could be correlated with the inhibitory action of PD98059.^[^ ^254^ ^]^In Vicia faba L. plants, application of SB203580A, which is an inhibitor of p38 MAP kinase and of pyridinyl imidazole, interrupted ABA‐induced stomatal closure modulated ABA‐induced H_2_O_2_ generation in guard cells, and inhibited K^+^ currents across the plasma membrane of guard cells.^[^ ^255^ ^]^

##### Indirect Chemical Manipulation of SnRK2 via Casein Kinase 2 (CK2)

The casein kinase 2 proteins (CK2s) are capable of phosphorylating SnRK2, an ABA‐signaling core component. The CK2s are evolutionary conserved and ubiquitous eukaryotic serine/threonine protein kinases. In plants, CK2s are related to plant growth, development, and abiotic stress responses.^[^ ^256^ ^]^ There are more reports concerning CK2s in animals than in plants, however, and the reports from animals include the ability of CK2s to phosphorylate more than 300 proteins.^[^ ^257^ ^]^ The substrates of plant CK2s include SnRK2 proteins, which represent a component of the core ABA signaling pathway. CK2s interact with SnRK2 subfamily III by phosphorylation. CK2 is a tetrameric holoenzyme with two catalytic alpha subunits and two regulatory beta subunits, but these two subunits have different functions. A third catalytic subunit has also been reported, although it is less‐well characterized than the other two subunits.^[^ ^258^ ^]^ The CK2s phosphorylate a cluster of conserved serine residues in the ABA box of group III SnRK2s (a recognizable motif at the C‐terminal of SnRK2 that is involved in ABA response and that is also known as domain II), thereby increasing its binding to PP2C. The above phenomenon also promotes protein degradation. This ABA box is the docking site for PP2Cs whose action prevents SnRK2 function in the absence of ABA. CK2s are therefore significant because of their direct interactions with group III SnRK2s. The involvement of CK2 in ABA signaling has been experimentally assessed. The maize homolog of CK2 phosphorylated the ABA box of ZmOST1 and increased its turnover and promotes PP2C binding. Arabidopsis contains the subunits CK2*α*1, CK2*α*2, CK2*α*3, CK2*α*4, CK2*β*1, CK2*β*2 CK2*β*3, and CK2*β*4. Among these, CK2*α*1 and CK2*α*2 positively regulated the expression of genes that depend on ABRE, as found in protoplast transient expression experiments using the RD29B‐GUS system.^[^ ^259^ ^]^ In contrast, the same research determined that the CK2*β* subunits negatively regulate the ABRE‐dependent gene expression mediated by the subclass III SnRK2–AREB/ABF pathway and by CK2*α*1/2. CK2*α*1/2 functioned as positive regulators both in the presence and absence of exogenous ABA. The negative regulation of ABRE‐dependent gene expression by CK2*β* was dependent on exogenous ABA. The over‐expression of AREB1 counteracted the ABRE‐dependent gene expression induced by CK2*α*1. This may be due to a transcription factor downstream of CK2*α*1.^[^ ^259^ ^]^ It follows that the chemical modulation of either the catalytic or regulatory subunits of CK2 may affect ABA signaling.

Inhibitors of CK2: The compounds 5,6‐dichloro‐1‐(b‐d‐ribofuranosyl) benzimidazole (DRB) and heparin act as CK2 inhibitors. Their CK2 inhibitory action was evaluated in relation to salicylic acid (SA), the action of which is often integrated with that of ABA. SA treatments increased nuclear CK2 activity. DRB, partially inhibited the CK2 kinase activity in leaf extracts treated with SA or water. Heparin strongly inhibited the kinase activity of CK2.^[^ ^260^ ^]^

CK2 inhibitors in mammalian systems: The mammalian homologs of CK2 have been considered attractive targets for cancer therapy. The available CK2 inhibitors affect mammalian cell proliferation, transformation, apoptosis, and senescence.^[^ ^261^ ^]^ By virtual screening of the coumarin compounds in MMS‐in‐house database (which specifically targeted the ATP binding site of CK2) researchers identified the following compounds as CK2 inhibitors: hematein (screened from the natural product library NPL 400 of Timtect Inc., Newark, DE),^[^ ^262^ ^]^ ellagic acid (a naturally occurring tannic acid derivative), tetrabromocinnamic acid (TBCA), 1,8‐dihydroxy‐4‐nitroxanthen‐9‐one (MNX), 8‐hydroxy4‐methyl‐9‐nitrobenzo[g]chromen‐2‐one (NBC), and 3,8dibromo‐7‐hydroxy‐4‐methylchromen‐2‐one(DBC).^[^ ^263^ ^]^ The coumarin moiety can be considered as an attractive CK2 inhibitor scaffold. Three virtual screening methods (Bayesian model, pharmacophore hypothesis, and molecular docking) identified five new CK2 inhibitors with novel scaffolds from two large chemical libraries, Specs (AE‐848/13422091, AP‐906/42087542, AG‐690/36808010) and Enamine (T5327348, T5765269).^[^ ^261^ ^]^ The above data may help guide the development of inhibitors of plant homologs of CK2.

## 7 ABA Catabolism

Plants regulate their endogenous ABA levels by both biosynthesis and catabolism. Following its rapid and specific bioactivity, ABA is rapidly catabolized into hydroxylated and conjugated forms. Catabolism reduces plant ABA levels and maintains a pool of ABA conjugates. It generates a set of catabolic intermediates/products that are also capable of activating a subset of ABA receptors, allowing further plant responses to long‐term stress conditions.^[^ ^264^ ^]^ ABA catabolism, therefore, not only maintains endogenous ABA levels but also provides a source of ABA agonists with subtle activity.

### 7.1 An Overview of ABA Catabolic Pathways

**Figure 10:**
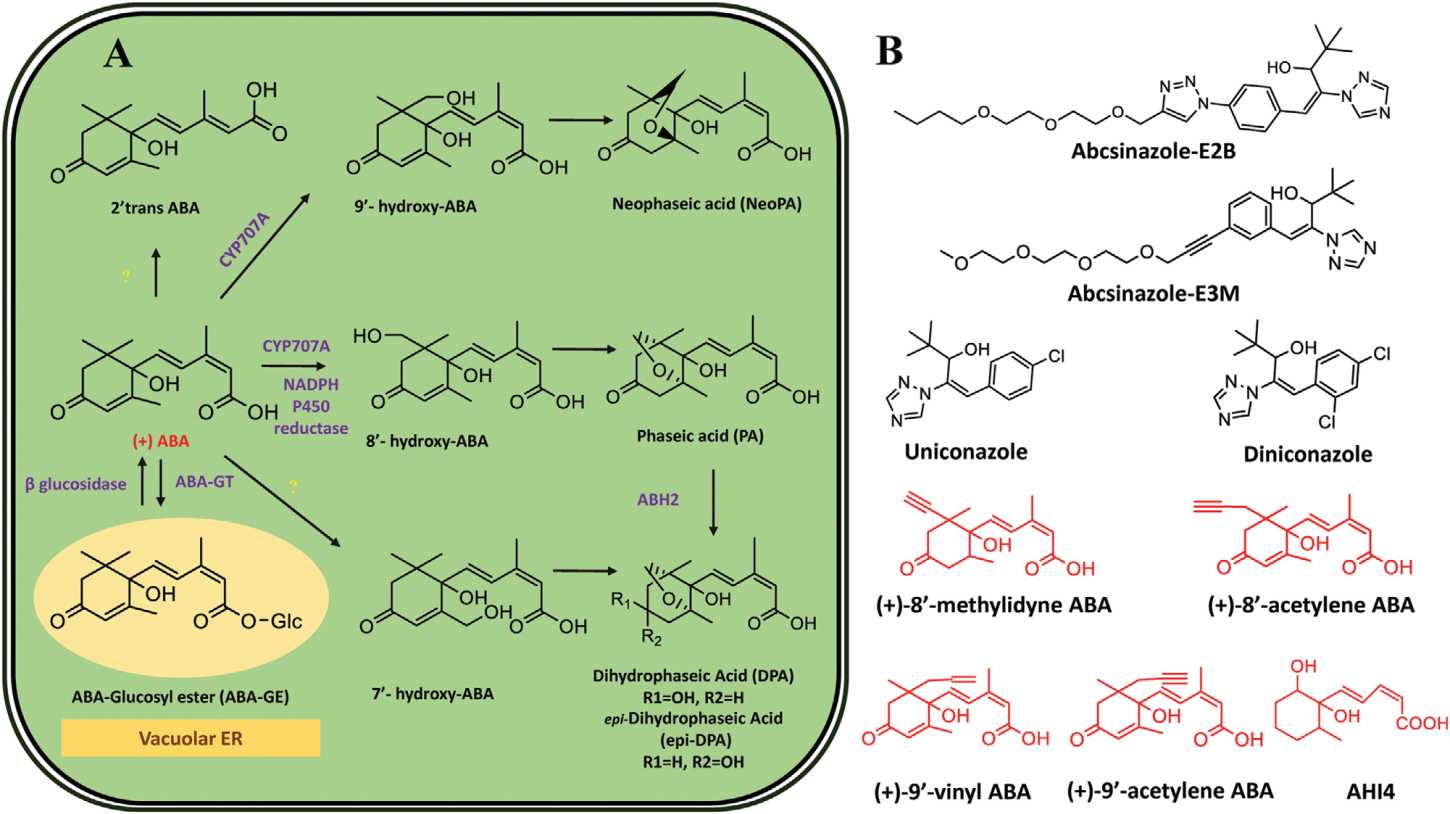
A) ABA catabolic pathways and B) chemical modulators. Azole‐type inhibitors are in black, ABA analogs are in red. Among the three hydroxylation pathways, ABA 8ʹ‐hydroxylation is thought to be the major ABA catabolic route. ABA is also inactivated to ABA glucosyl ester (ABA‐GE) by ABA‐glucosyltransferase (ABA‐GT), and ABA‐GE is converted to free ABA by *β*‐glucosidase. The enzyme CYP707A is a potential target for inhibition of the catabolic process. The compound dihydrophaseic acid (DPA) is the primary end product of ABA catabolism. ABA‐GE is stored in the endoplasmic reticulum (ER) and vacuoles.

ABA is subjected to catabolism by sequential hydroxylation and conjugation reactions (**Figure** **10**A). During hydroxylation, each of the methyl groups in the ABA ring structure (C‐7ʹ, C‐8ʹ, and C‐9ʹ) undergo oxidation by three ABA hydroxylation pathways, giving rise to three forms of hydroxylated ABA with substantial biological activities.^[^ ^265^ ^]^ However, these products are inactivated in subsequent steps. In addition to hydroxylation pathways, ABA and its hydroxylated catabolites (8’‐hydroxy ABA, Phaseic acid (PA), dihydrophaseic acid (DPA), and *epi*‐DPA) are conjugated with glucose.^[^ ^266^ ^]^ The inactive form, 2ʹ‐*trans*‐ABA, is also found at a low level.^[^ ^119^ ^]^

The ABA hydroxylation reactions are significant because they can progressively reduce the availability of ABA receptor‐binding molecules and their affinity to PYLs. ABA 8ʹ‐hydroxylation is a crucial step in ABA catabolism.^[^ ^119^ ^]^ This step, which is catalyzed by cytochrome P450‐type monooxygenases (CYP707A), requires both nicotinamide adenine dinucleotide phosphate (NADPH) and P450 reductase.^[^ ^267^ ^]^ The unstable 8ʹ‐hydroxy‐ABA undergoes spontaneous isomerization to generate PA, which has weak ABA‐like activity.^[^ ^239^, ^268^ ^]^ Rehydration, submergence, and developmental processes (e.g., germination) induce transcription of the CYP707A genes.^[^ ^267^ ^]^ The enzyme CYP707A3 ABA 8ʹ‐hydroxylase is primarily involved in dehydration and rehydration responses in Arabidopsis.^[^ ^269^ ^]^ The PA reductase (encoded by *ABA HYPERSENSITIVE 2 (ABH2)* ^[^ ^264^ ^]^) further metabolizes PA, yielding DPA or *epi*‐DPA. These products were found to have almost no detectable biological activity.^[^ ^268^ ^]^ The metabolites of the other two hydroxylation pathways have substantial biological activity.^[^ ^265^, ^270^ ^]^ Spontaneous isomerization of unstable 9’‐hydroxy‐ABA results in the formation of neophaseic acid (NeoPA)‐like PA.^[^ ^265^ ^]^ Although a minor catabolite, NeoPA has been found in a number of plant species.^[^ ^265^ ^]^ Substantial accumulation of NeoPA has been observed in developing seeds of Arabidopsis and *Brassica napus*.^[^ ^265^ ^]^ The CYP707A enzymes were less involved in in vitro 9ʹ‐hydroxylation of ABA as observed in Arabidopsis.^[^ ^271^ ^]^ The Arabidopsis *cyp707a* mutant had lower endogenous NeoPA and PA levels than the wild type.^[^ ^271^ ^]^ Hence, NeoPA is a side reaction product of CYP707A. The gene encoding 7’‐hydroxylase has not been identified. CYP707A acts as the key enzyme in hydroxylation reactions, and ABA 8‐hydroxylation is the key step.

The conjugation reactions act on hydroxylated catabolites of ABA to yield glucose conjugates.^[^ ^119^, ^272^ ^]^ The ABA glucosyl ester (ABA‐GE) is the most prevalent form of conjugated catabolites.^[^ ^273^ ^]^ The enzyme glucosyltransferase (ABA‐GT) catalyzes the glucosylation of the carboxyl group on ABA.^[^ ^274^ ^]^ Of the eight reported ABA‐GTs in Arabidopsis, only one (UGT71B6) selectively acts on (+)‐ABA rather than (−)‐R‐ABA^[^ ^275^ ^]^ Functional ABA is regenerated by *β*‐glucosidase hydrolyzation of ABA‐GE.^[^ ^178^, ^276^, ^277^ ^]^ In Arabidopsis, two homologous *β*‐glucosidases, AtBG1 and AtBG2, localize to the ER and vacuole, respectively.^[^ ^274^, ^276^, ^277^ ^]^ The enzymatic activity of AtBG1 is enhanced by dehydration‐induced polymerization.^[^ ^276^ ^]^ The AtBG2 protein is polymerized in high molecular weight complexes where it is protected from degradation during dehydration stress. The *atbg1* mutant had significantly reduced ABA levels in the extracellular space. Although this phenomenon was not observed in the *atbg2* mutant, the *atbg1 atbg2* double mutant had increased sensitivity to drought stress.^[^ ^277^ ^]^ Regulation of the endogenous ABA concentration via *β*‐glucosidase‐mediated ABA generation from ABA‐GE is significant during environmental stress.^[^ ^278^ ^]^ The conjugation reactions are essential for providing the required concentrations of ABA at target sites during stress and nonstress conditions.

#### 7.1.1 Important Hydroxylation Products Resulting from ABA Catabolism

##### 8ʹ‐Hydroxy‐ABA and Phaseic Acid

PA generated by 8ʹ‐hydroxy‐ABA is capable of PYL activation. A final ratio of 98:2 was observed for 8ʹ‐hydroxy‐ABA and PA when in equilibrium at 25 °C.^[^ ^279^ ^]^ PA is an important isomer that is specifically present in barley aleurone layers.^[^ ^232^, ^270^, ^278^, ^280^ ^]^ PA has ABA‐like effects, i.e., it causes stomatal closure in some species, such as *Amaranthus powelli*, *Hordeum vulgare*, and *Xanthium strumarium*,^[^ ^281^ ^]^ but its effects on stomatal closure are weaker in barley and maize.^[^ ^281^ ^]^ PA did not inhibit wheat seed germination even at a concentration 50‐fold higher than that of ABA.^[^ ^282^ ^]^ PA inhibition of the germination of barley immature embryos was only 10% of that of ABA.^[^ ^283^ ^]^ In Arabidopsis, the application of high PA concentrations impedes stomatal closure and reduces photosynthesis.^[^ ^232^ ^]^ Most of the PA‐responsive genes in Arabidopsis treated with 50 × 10^−3^ m PA overlapped with ABA‐responsive genes. The effect on differentially expressed genes, however, was smaller for the PA treatment than for the ABA treatment.^[^ ^264^ ^]^ Therefore, the effect of PA treatment on the plant transcriptome is much weaker than that of ABA treatment. A disruption in PA degradation may result in high PA levels and reduced ABA content. Plants with disrupted PA degradation were not slow growers as observed with certain ABA biosynthetic mutants, suggesting that PA compensates for the effect of low ABA. Furthermore, even though it reduced photosynthesis,^[^ ^281^ ^]^ PA did not affect the rates of CO_2_ assimilation. PA was simultaneously recognized as an effective ligand of the PYL family.^[^ ^264^ ^]^ ABI2 activity was regulated by PA, in the presence of PYL9, PYL8, and PYR1.^[^ ^239^ ^]^ ABA, however, was more effective than PA in inhibiting PP2C activity. Regarding receptor sensitivity, PYR1, PYL1, and PYL10 were 40‐fold less sensitive to PA than to ABA, and PYL2, PYL4, PYL5, and PYL6 were between 10‐and 20‐fold less sensitive to PA than to ABA. Among PYLs, PYL3 is especially responsive to PA, because its IC_50_ value was four‐fold higher for PA than for ABA.^[^ ^281^ ^]^

##### Dihydrophaseic Acid and *epi*‐Ihydrophaseic Acid

PA (which is capable of activating a subset of ABA receptors) is converted to DPA and *epi*‐DPA in plants. The native DPA content in mature beans was 100‐fold higher than that of ABA^[^ ^284^ ^]^ due to the catabolism of ABA. More than 50% of ABA was converted to DPA after 11 h, with only about 18% of the original ABA content remaining.^[^ ^284^ ^]^ The endogenous level of *epi*‐DPA is about 10% of the level of DPA in plants. These findings suggest that ABA is prone to degradation and that DPA is the main metabolite of ABA. Nevertheless, 10 × 10^−6^ m DPA did not cause stomatal closure in any species. Moreover, unlike ABA and PA, DPA did not affect rates of CO_2_ assimilation.^[^ ^281^ ^]^ Thus, DPA and *epi*‐DPA levels in plants may indicate the efficiency of ABA catabolic reactions as well as the level of stress.

##### ABA‐GE

Among ABA conjugates, ABA‐GE is the most abundant and serves as the ABA storage form. ABA‐GE was initially described as a physiologically inactive catabolic product of ABA.^[^ ^285^ ^]^ ABA‐GE has been detected in various organs of different plant species, such as immature fruit of yellow lupin,^[^ ^266^ ^]^ the pseudocarp of field rose,^[^ ^23^ ^]^ and the leaves of *Xanthium* and spinach.^[^ ^266^, ^286^ ^]^ ABA‐GE pools were observed in the vacuole and the ER.^[^ ^178^ ^] [^ ^287^, ^288^, ^289^ ^]^ The endogenous levels of ABA‐GE were at their highest before ABA levels were at their highest in *Cistus albidus* plants.^[^ ^290^ ^]^ The compartmentalization of ABA‐GE is necessary for ABA homeostasis because one‐step hydrolysis readily generates ABA from ABA‐GE. In addition to ABA‐GE, hydrolysis of ABA also generates conjugated glucosides. *Vice versa*, conjugation of ABA with glucose produces ABA‐GE.^[^ ^115^, ^285^ ^]^ Plant ABA levels are dynamically maintained by synthesis, transport, and catabolism.^[^ ^290^, ^291^ ^]^ During abiotic stress conditions, the *β*‐glucosidases in the ER and vacuoles act on ABA‐GE to rapidly generate free ABA. Elevated ABA‐GE levels were reported during dehydration as well as during seed development and germination.^[^ ^120^, ^178^, ^266^, ^292^ ^]^ The content of ABA‐GE in xylem sap content was increased in plants subjected to drought, salt, or osmotic stress.^[^ ^293^ ^]^ Researchers detected a correlation between ABA and ABA‐GE contents under water stress.^[^ ^294^ ^]^ In addition to its role in the long‐distance transport of ABA,^[^ ^273^, ^293^, ^295^ ^]^ ABA‐GE was reported to be an allelopathic compound in *Citrus junis*.^[^ ^296^ ^]^ Higher concentrations of ABA‐GE than ABA were reported in agricultural soils, suggesting that ABA‐GE may be taken up roots.^[^ ^119^, ^121^, ^293^ ^]^ Researchers have also found that ABA‐mediated stomatal closure during drought occurs independently of root ABA biosynthesis.^[^ ^178^ ^]^ Bioactive ABA is generated from inactive ABA‐GE through the catalysis of ER‐located *β*‐glucosidase 1. ABA levels in root tips increase slowly and are stimulate enzymatic activity for nitrate metabolism. A negative feedback loop involving the PP2C protein ABI2 and the AtNPF6.3 transporter reduces the nitrate influx. The increase in the level of root‐tip‐localized ABA also negatively regulates the expression of the *SCARECROW* transcription factor gene, thus providing a sensitive mechanism for modulating root growth in response to environmental changes.^[^ ^297^ ^]^ Together, these results indicate that ABA‐GE levels in plants and the ABA‐GE hydrolysis enzyme *β*‐glucosidase 1 have significant functions in ABA catabolism.

### 7.2 Inhibitors of ABA 8ʹ‐Hydroxylases (P450 CYP707A)

The ABA 8‐hydroxylase enzyme CYP707A selectively recognizes the naturally occurring (+)‐S‐ABA enantiomer rather than (−)‐R‐ form.^[^ ^267^, ^280^ ^]^ In Arabidopsis, multiple mutants of CYP707A contained large quantities of endogenous ABA, whereas CYP707A overexpressors contained reduced quantities of endogenous ABA.^[^ ^40^, ^269^, ^271^, ^298^, ^299^ ^]^ Given its role in regulating endogenous ABA levels, CYP707A is the target of nearly all inhibitors of ABA catabolism. There are two types of inhibitors of CYP707A: azole types and ABA analogs (Figure 10 B).

#### 7.2.1 Azole‐Type Inhibitors

Azole type inhibitors are a series of researcher‐developed compounds that are based on gibberellin biosynthesis inhibitors Uniconazole, which was the first developed azole‐type inhibitor,^[^ ^300^ ^]^ targets the conversion of ABA to PA. Although uniconazole was first used as an inhibitor of gibberellin biosynthesis,^[^ ^300^, ^301^ ^]^ it also conferred drought‐stress tolerance and induced endogenous ABA levels that were 2‐fold higher than in the control. These effects were not counteracted by the coapplication of uniconazole and gibberellin GA4, indicating that uniconazole is a potent competitive inhibitor (*K*_i_ = 8.0 × 10^−9^ m) of the CYP707A enzyme.^[^ ^300^ ^]^ The fungicide diniconazole, which is structurally similar to uniconazole, was similar to uniconazole in that it inhibited CYP707A and induced the expression of ABA‐responsive genes.^[^ ^302^ ^]^ However, the effects of azole inhibitors on cytochrome P450 monooxygenases were found to be nonspecific because other cytochrome P450 monooxygenases were also inhibited. This may explain why uniconazole and diniconazole reduce plant growth.^[^ ^303^ ^]^ More specific inhibitors of CYP707A were developed based on the uniconazole scaffold structure. Unlike uniconazole, the compounds abscinazole‐E2B and abscinazole‐E3M^[^ ^115^, ^287^ ^]^ selectively inhibited CYP707A rather than CYP701A, and did not alter gibberellin biosynthesis.^[^ ^21^, ^115^, ^304^ ^]^ Abscinazole‐E2B and abscinazole‐E3M target the P450 active site with the heme‐iron atom and interacting with the adjoining protein residues.^[^ ^305^ ^]^ The lone pair of electrons on the ligand nitrogen of the two inhibitors showed an intrinsic affinity for the heme iron, which led to heme coordination. The structural flexibility of the P450 active site may allow inhibitor–enzyme interactions, even though the geometry of the azole‐type of inhibitor does not entirely coincide with that of the active site. This feature favors the nitrogen interaction with the heme iron. In contrast, the steric requirement of the CYP707A active site for structural ABA analogs ligands is comparatively strict.^[^ ^306^, ^307^ ^]^

#### 7.2.2 ABA Analogs

ABA analogs have been found with specific ABA catabolic inhibitory activity. Two types of these analogs have been reported: suicide (mechanism‐based) inhibitors and competitive inhibitors.^[^ ^307^ ^]^ In designing suicide inhibitors, researchers modified the ABA structure at the 8ʹ‐ and 9ʹ‐positions, based on the observation that terminal acetylene and olefin may serve as mechanism‐based inhibitors of P450 enzymes.^[^ ^307^ ^]^ Examples of suicide inhibitors include (+)‐8ʹ‐ methylidyne‐ABA, (+)‐8ʹ‐acetylene‐ABA, (+)‐9ʹ‐vinyl‐ABA, and (+)‐9ʹ‐acetylene‐ABA; these compounds showed irreversible CYP707A inhibitory activity with *Ki* values of 19.0, 1.1, 5.5, and 0.27 × 10^−6^ m, respectively.^[^ ^272^ ^]^ Despite the preferred selectivity of CYP707A for 8ʹ‐methyl‐substituted ABA, (+)‐9ʹ‐acetylene‐ABA was more potent in inhibiting CYP707A. This observation suggested that CYP707A is capable of recognizing both C‐8ʹ and ‐9ʹ methyl groups of ABA. However, these compounds retained ABA agonist activity.^[^ ^184^ ^]^ To eliminate this activity, researchers investigated several competitive analogs that lacked structural features essential for ABA agonist activity.^[^ ^306^, ^308^ ^]^ Among them, ABA 8ʹ‐hydroxylase inhibitor 4 (AHI4) proved to be a strong inhibitor of CYP707A with no observed ABA activity (e.g., growth arrest or inhibition of seed germination).^[^ ^272^, ^308^ ^]^ Accordingly, AHI4 was able to modulate ABA levels without strong growth inhibition. It follows that in addition to being useful for studying ABA metabolic regulation, these ABA biosynthesis inhibitors may have agricultural applications.

## 8 Conclusion

The practical aim of chemical manipulation of ABA signaling has been the development of agrochemicals that protect plants from stress. The main focus has been on ABA biosynthesis, perception, and catabolism. To a lesser extent, ABA transport has also been studied using small molecule inhibitors or competitors.

The reported chemical modulators of ABA signaling include several small molecules. Among the chemical modulators of ABA biosynthesis, compounds that target the key ABA biosynthesis enzyme NCED may be considered as most successful. A reduction in ABA levels caused by NCED inhibitors may lead to the release of seed dormancy. The ABA transporter proteins have been chemically modulated by a collection of compounds that are relatively complex in structure and that have a broader substrate range. Most of these compounds reduced the uptake of ABA or ABA‐GE. In ABA perception, both agonists and antagonists have demonstrated promise as agrochemicals. The agonists that targeted subfamily III PYLs and that increased or otherwise improved ABA‐like activity (e.g., quinabactin, cyanabactin, AMF4, and opabactin) are promising protectants against moderate drought. By closing stomata and up‐regulating a collection of ABA‐responsive genes, they reduce transpiration. The reported antagonists block PYL‐PP2C interactions or stabilize nonproductive ligand‐PYL‐PP2C interactions, preventing further downstream signaling events. The halting of ABA‐mediated stress responses by antagonists may be useful for the chemical control of plant senescence, as reported for the antagonist AA1. ABA catabolism was chemically manipulated by abscinazole‐E2B and abscinazole‐E3M, which selectively inhibited the CYP707A enzyme. Among the available inhibitors of ABA catabolism, these selective inhibitors appear to function without affecting plant growth. Therefore, it is evident that chemical manipulation efforts have been successful in affecting the most critical areas of ABA signaling.

## 9 Prospects and Challenges

As noted earlier, the practical aim of chemical manipulation of ABA signaling has been the development of plant stress‐protectants, in the form of agrochemicals. The development of such agrochemicals is an alternative to plant breeding for resistance against biotic and abiotic stress‐factors. Such breeding is often time‐consuming and limited by the features of the plant genome. As agrochemicals, ABA signaling modulators are a much more straightforward solution than breeding to plant stress management. A chemical can be developed relatively quickly, and its use can be controlled. In the current report, we have reviewed the efforts to chemically manipulate several ABA signaling events in plants. We now consider the potentials and challenges in the development and application of chemicals that manipulate ABA signaling.

### 9.1 Targets for Chemical Manipulation

In general, the most attractive targets for the manipulation of ABA signaling are relatively conserved and substrate‐specific proteins. Because they are affected by many factors, downstream components of the signaling cascade are generally not attractive targets. The most attractive targets for chemical manipulation are PYLs. The subfamily III PYLs, in particular, have been important in the chemical manipulation of plant water use. Proper positioning of a small molecule in the PYL active site while satisfying steric requirements for the ligand–receptor interactions are essential features of effective ABA agonists. Mimicking and/or enhancing the critical interactions of natural ABA and native receptor proteins by an assembly of natural or unnatural structural groups has resulted in promising ABA agonists. Targeting PYLs subfamilies that are required for a particular physiological response may lead to the development of agrochemicals that are specific for different growth stages. Varying levels of antagonism may be achieved by changing the extent to which the ligand–receptor interaction is favored. Therefore, ABA receptors are promising targets of chemical modulation. It is therefore not surprising that research on ABA perception is currently concerned with the characterization of the interactions between ABA or an ABA analog and specific PYL subclasses and family members. The decoded structural, chemical–genetic, and biological information is much greater for PYLs than for the other ABA signaling core components.

Many of the PP2Cs, SnRK2s, and the related transcription factors in ABA signaling have yet to be characterized in detail. The detailed characterization may provide valuable clues for the design of chemical modulators. The recent identification of novel SnRK2s that is responsible for osmotic stress tolerance may suggest new targets for chemical manipulation. Accordingly, many prospects await for the use of target proteins for chemical manipulation. One challenge to the identification of new chemical manipulation targets is insufficient signaling information. In ABA biosynthesis and catabolism, more studies are needed on the catalytic enzymes responsible for specific steps. The plastid‐cytosol transport of ABA biosynthesis intermediates is an area requiring more research. Compared to PYLs, a dedicated family of ABA‐specific transporters has not yet been reported. Activators of ABA transporter proteins are yet to be studied, especially with respect to their substrate specificity. A second challenge is the interconnections of multiple biochemical pathways and their metabolic products. Targeting the early events of ABA biosynthesis such as the xanthophyll cycle enzymes via small molecules is problematic because the enzymes are required not only for the accumulation of ABA but also for the photoprotection of photosynthesis. In addition, the cellular availability of some ABA‐related metabolites (e.g., ABA‐GE) is often controlled by multiple transporter proteins with varying and nonspecific substrate specificities. Voltage gradients across the plasma membrane are important for the function of ABA transporter proteins, and this represents a challenge for the chemical–genetic approach, because disrupting a voltage gradient may affect several other critical cellular processes (e.g., transmembrane ion transport). A third challenge is off‐target binding, as it can limit the specificity of the small molecule. Therefore, locating the perfect target for chemical manipulation should always be supported by a thorough genetic understanding. Chemical manipulation of ABA signaling should be integrated with genetic approaches because genetic approaches can validate the effect of the chemical inhibitor or modulator on a particular event, and because genetic changes could sensitize the plant to the chemicals of interest.

Targeting a particular protein with a small molecule inhibitor or activator would be faster and simpler than studying the protein using genetic approaches, assuming that an acceptable small molecule is available. Off‐target binding, however, is a significant challenge because it limits specificity. Although nonspecific inhibitors have been effective in both animals and plants, the substantial expansion of protein families across organisms can lead to off‐target binding. Given the complexity of signaling events, screening for the ideal signaling inhibitor or modulator would be challenging. Therefore, the selection of the target for chemical manipulation requires substantial attention.

### 9.2 Novel Sources for Chemical Modulator Structure

Insights into the structure of candidates that can be developed for chemical manipulation can be obtained from many sources, i.e., from binding‐mode information of ABA‐PYLS‐PP2Cs, available ABA chemical modulators, ABA catabolic products, high throughput virtual screening platforms, and chemical modulators of homologous proteins in plants and animals. Insights from natural ABA signaling events are invaluable for understanding the function of a chemical modulator. The naturally occurring (+) ABA is the best example of why interactions with the receptor/coreceptor should be favored. Structural information on existing chemical modulators and their identified drawbacks are used for modifying the structures of small molecule so as to improve their effects with respect to agricultural production. Furthermore, understanding the factors that stabilize ligand–receptor interactions has contributed much to the design of ABA analogs. This has resulted in the development of small molecules that have superior ABA‐like effects while lacking the exact ABA structural components, as demonstrated by the ABA agonist cyanabactin. Results obtained with cyanabactin suggest that providing the minimum structural characteristics to activate a particular receptor is sufficient in designing functional analogs of ABA. Determinants of the agonist and antagonist nature of the small molecules are also important factors to be considered, as exemplified in the development of the AS(n) series of compounds. Catabolism of ABA under natural conditions generates yet another subset of molecules that are capable of activating PYLs. The rapid and reversible effects of ABA, its transportation form (ABA‐GE), and the long‐term effects of the ABA metabolic products help land plants deal with the adverse effects of water scarcity. Therefore, in designing ABA analogs to be used as plant drought protectants, a combination of molecules with quicker ABA‐like effects and with long‐term and more stable reversible effects would be useful. Large‐scale screening of small molecule libraries in search of structural moieties that best bind to the target is facilitated by virtual screening. This initial screening method has successfully identified several structural motifs that have been investigated and developed as chemical modulators of PYLs. Regarding ABA transport, the ABC superfamily of transporter proteins is widely distributed in both plants and animals. One of the clinical applications of human ABC transporter modulators is the development of anticancer drugs. The active efflux of anticancer drugs by ABC transporter proteins, however, leads to multidrug resistance (MDR), which is the leading cause of failure in cancer therapy. As a consequence, several inhibitors or modulators of the human ABC superfamily are being developed^[^ ^309^ ^]^ to prevent MDR. Such compounds may be used against plant homologs of their targets in order to study events related to ABA transport. Therefore, the strategically obtained information on natural and unnatural structures is clearly an advantage in chemical manipulation.

### 9.3 Wider Range of Application of Chemical Modulators

New applications could be developed for the use of ABA signaling modulators in agriculture. Moderating ABA accumulation in seeds by ABA biosynthesis inhibitors could be used to achieve uniform seed germination. The loss of flowers, buds, fruits, pods at an earlier stage due to an endogenous phytohormonal imbalance may be corrected by the application of ABA metabolic inhibitors. Such inhibitors could also be used to delay fruit ripening, achieve uniform ripening, and prevent early senescence. Small molecule antagonists of ABA receptors may prove useful in horticulture and postharvest industries, where the maintenance of fresh produce is important. ABA catabolic inhibitors may help to maintain higher endogenous ABA levels in plants under adverse conditions. They may also be useful in preventing early germination and in maintaining the dormancy of seeds and buds or corms. ABA catabolic inhibitors could be useful in the storage of propagules of staple crops. When a plant is being attacked by a pathogen that blocks host plant ABA biosynthesis, the application of an ABA biosynthesis activator or an ABA catabolic inhibitor could prevent or reduce the infection. These potential applications, however, will require a better understanding of the cross‐talk between hormones in plants.

Several challenges must be overcome in the development of agrochemicals that function as chemical modulations of ABA signaling. One challenge concerns a lack of specificity: although the targeting of a particular protein with a small molecule inhibitor or activator would be faster and simpler than genetic manipulation, off‐target binding could cause the small molecule to have limited specificity. Given the complexity of signaling events and the fact that most signaling components have multiple closely related family members, screening for the ideal signaling inhibitor/modulator can be challenging. Furthermore, depending on the developmental‐specific expression of the targeted signaling component, the optimal activity of a promising small molecule is often limited to a particular plant growth stage of plants, which would restrict the use of the small molecule. This has been evident in agonists developed for PYLs. Another concern is that the anatomies of leaves, seed pericarps, and testa in crop plants can be different from those in the model plants that were used for the development of the small molecules; the in vivo activity of a small molecule in crop plants will depend on how efficiently the chemical enters the plants, and this could differ greatly among plant species. The analog should act in concert with the xylem sap components (cytokinin, malate, ethylene precursors, etc.), pH, and other conditions. These may vary among species and are often unknown. For these reasons, the developed agonist or antagonist may not display the expected effects under natural conditions. On the other hand, some ABA agonists and antagonists including AM1, AMF4, cyanabactin, PANme, and AA1 have been found to be active not only in the model plant Arabidopsis but also in crops like rice, soybean, and tomato at different developmental stages. The chemical modulators must be tested in dose‐response studies under natural conditions to determine the toxicity levels imposed by the chemical on the plant or plant part; such testing is important because some specific structural moieties tend to cause oxidative stress at high concentrations. Movement of the modulator in the plant and its retention in the plant have yet to assess for those chemical modulators that have already been developed. Another challenge involves the targeting of the early events of ABA biosynthesis. For example, the targeting of xanthophyll cycle enzymes with small molecules is problematic, because the enzymes are required not only for the accumulation of ABA but also for photoprotection of photosynthesis. In addition, the cellular availability of some ABA‐related metabolites (e.g., ABA‐GE) is often controlled by multiple transporter proteins with varying and nonspecific substrate specificities. Voltage gradients across the plasma membrane are important for the function of ABA transporter proteins. This is challenging for the chemical–genetic approach, because disrupting a voltage gradient may affect other critical cellular processes (e.g., transmembrane ion transport). Chemical manipulation of ABA signaling can be more meaningful and powerful when it is integrated with a genetic approach, because a genetic approach can validate the effect of the chemical inhibitor or modulator on a particular event, and because genetic changes could sensitize the plant toward the chemicals of interest.

In conclusion, chemical manipulation of ABA signaling is a novel and promising approach for developing next‐generation agrochemicals to control abiotic and biotic stress in plants.

## Conflict of Interest

The authors declare no conflict of interest.

## References

[1] S. Savaldi‐Goldstein , T. J. Baiga , F. Pojer , T. Dabi , C. Butterfield , G. Parry , A. Santner , N. Dharmasiri , Y. Tao , M. Estelle , J. P. Noel , J. Chory , Proc. Natl. Acad. Sci. USA 2008, 105, 15190. 18818305 10.1073/pnas.0806324105 PMC2567513

[2] D. Huang , M. R. Jaradat , W. Wu , S. J. Ambrose , A. R. Ross , S. R. Abrams , A. J. Cutler , Plant J. 2007, 50, 414. 17376162 10.1111/j.1365-313X.2007.03056.x

[3] H. Kende , J. A. D. Zeevaart , Plant Cell 1997, 9, 1197. 12237383 10.1105/tpc.9.7.1197 PMC156991

[4] R. Finkelstein , The Arabidopsis Book 2013, 11, e0166. 24273463 10.1199/tab.0166 PMC3833200

[5] M. Cao , X. Liu , Y. Zhang , X. Xue , X. E. Zhou , K. Melcher , P. Gao , F. Wang , L. Zeng , Y. Zhao , Y. Zhao , P. Deng , D. Zhong , J.‐K. Zhu , H. E. Xu , Y. Xu , Cell Res. 2013, 23, 1043. 23835477 10.1038/cr.2013.95 PMC3731570

[6] R. R. Finkelstein , S. S. L. Gampala , C. D. Rock , Plant Cell 2002, 14, S15. 12045268 10.1105/tpc.010441 PMC151246

[7] A. S. Raghavendra , V. K. Gonugunta , A. Christmann , E. Grill , Trends Plant Sci. 2010, 15, 395. 20493758 10.1016/j.tplants.2010.04.006

[8] N. Kitahata , T. Asami , J. Plant Res. 2011, 124, 549. 21461661 10.1007/s10265-011-0415-0

[9] N. Rajagopalan , K. Nelson , A. Douglas , V. Jheengut , I. Alarcon , S. McKenna , M. Surpin , M. Loewen , S. Abrams , Biochemistry 2016, 55, 5155. 27523384 10.1021/acs.biochem.6b00605

[10] V. Chinnusamy , Z. Gong , J.‐K. Zhu , J. Integr. Plant Biol. 2008, 50, 1187. 19017106 10.1111/j.1744-7909.2008.00727.x PMC2862557

[11] F. Hauser , R. Waadt , J. I. Schroeder , Curr. Biol. 2011, 21, R346. 21549957 10.1016/j.cub.2011.03.015 PMC3119208

[12] J. D. M. Helander , A. S. Vaidya , S. R. Cutler , Bioorg. Med. Chem. 2016, 24, 493. 26612713 10.1016/j.bmc.2015.11.010

[13] Y. Ma , I. Szostkiewicz , A. Korte , D. Moes , Y. Yang , A. Christmann , E. Grill , Science 2009, 324, 1064. 19407143 10.1126/science.1172408

[14] S.‐Y. Park , P. Fung , N. Nishimura , D. R. Jensen , H. Fujii , Y. Zhao , S. Lumba , J. Santiago , A. Rodrigues , T.‐f. F. Chow , S. E. Alfred , D. Bonetta , R. Finkelstein , N. J. Provart , D. Desveaux , P. L. Rodriguez , P. McCourt , J.‐K. Zhu , J. I. Schroeder , B. F. Volkman , S. R. Cutler , Science 2009, 324, 1068. 19407142 10.1126/science.1173041 PMC2827199

[15] H. Fujii , V. Chinnusamy , A. Rodrigues , S. Rubio , R. Antoni , S.‐Y. Park , S. R. Cutler , J. Sheen , P. L. Rodriguez , J.‐K. Zhu , Nature 2009, 462, 660. 19924127 10.1038/nature08599 PMC2803041

[16] Y. Takahashi , Y. Ebisu , K.‐i. Shimazaki , Plant Physiol. 2017, 174, 815. 28438792 10.1104/pp.16.01825 PMC5462015

[17] N. Kim , S.‐J. Moon , M. K. Min , E.‐H. Choi , J.‐A. Kim , E. Y. Koh , I. Yoon , M.‐O. Byun , S.‐D. Yoo , B.‐G. Kim , Front. Plant. Sci. 2015, 6, 614. 26300907 10.3389/fpls.2015.00614 PMC4524894

[18] J. Leung , Plant Physiol. 2017, 173, 1939. 28356542 10.1104/pp.17.00299 PMC5373069

[19] Y. Ye , L. Zhou , X. Liu , H. Liu , D. Li , M. Cao , H. Chen , L. Xu , J.‐k. Zhu , Y. Zhao , Plant Physiol. 2017, 173, 2356. 28193765 10.1104/pp.16.01862 PMC5373061

[20] M. C. H. Cox , J. J. Benschop , R. A. M. Vreeburg , C. A. M. Wagemaker , T. Moritz , A. J. M. Peeters , L. A. C. J. Voesenek , Plant Physiol. 2004, 136, 2948. 15466223 10.1104/pp.104.049197 PMC523357

[21] W. Dejonghe , M. Okamoto , S. R. Cutler , Plant Cell Physiol. 2018, 59, 1490. 29986078 10.1093/pcp/pcy126

[22] S. Cutler , P. Rodriguez , R. Finkelstein , S. Abrams , Annu. Rev. Plant Biol. 2010, 61, 651. 20192755 10.1146/annurev-arplant-042809-112122

[23] B. V. Milborrow , J. Exp. Bot. 1970, 21, 17.

[24] B. V. Milborrow , J. Exp. Bot. 2001, 52, 1145. 11432933

[25] M. E. Saltveit , S.‐F. Yang , W. T. Kim , Discoveries in Plant Biology, World Scientific, Singapore 1998, p. 47.

[26] R. R. Finkelstein , Plant Cell 2006, 18, 786. 16595396 10.1105/tpc.106.041129 PMC1425860

[27] P. L. Rodriguez , J. Lozano‐Juste , A. Albert , in Advances in Botanica Research, Vol. 92 (Eds: Seo M., Marion‐Poll A.), Academic Press, San Diego, CA 2019, p. 51.

[28] J. Nyangulu , M. Galka , A. Jadhav , Y. Gai , C. Graham , K. Nelson , A. Cutler , D. Taylor , G. Banowetz , S. Abrams , J. Am. Chem. Soc. 2005, 127, 1662. 15701000 10.1021/ja0429059

[29] A. El‐Kereamy , S. Abrams , R. Hill , Nature 2006, 439, 290. 16421562 10.1038/nature04373

[30] K. Nakashima , Y. Fujita , N. Kanamori , T. Katagiri , T. Umezawa , S. Kidokoro , K. Maruyama , T. Yoshida , K. Ishiyama , M. Kobayashi , K. Shinozaki , K. Yamaguchi‐Shinozaki , Plant Cell Physiol. 2009, 50, 1345. 19541597 10.1093/pcp/pcp083

[31] N. Nishimura , A. Sarkeshik , K. Nito , S.‐Y. Park , A. Wang , P. C. Carvalho , S. Lee , D. F. Caddell , S. R. Cutler , J. Chory , J. R. Yates , J. I. Schroeder , Plant J. 2010, 61, 290. 19874541 10.1111/j.1365-313X.2009.04054.x PMC2807913

[32] M. del Carmen Rodríguez‐Gacio , M. A. Matilla‐Vázquez , A. J. Matilla , Plant Signal. Behav. 2009, 4, 1035. 19875942 10.4161/psb.4.11.9902 PMC2819511

[33] R. R. Finkelstein , C. D. Rock , in The Arabidopsis Book, Vol. 1 (Ed: Torii K.), American Society of Plant Biologists, Rockville, MD 2002, p. e0058.22303212

[34] P. A. Tuan , R. Kumar , P. K. Rehal , P. K. Toora , B. T. Ayele , Front. Plant Sci. 2018, 9. 10.3389/fpls.2018.00668 PMC5974119 29875780

[35] E. Nambara , M. Okamoto , K. Tatematsu , R. Yano , M. Seo , Y. Kamiya , Seed Sci. Res. 2010, 20, 55.

[36] K. P. Lee , U. Piskurewicz , V. Turecková , M. Strnad , L. Lopez‐Molina , Proc. Natl. Acad. Sci. USA 2010, 107, 19108. 20956298 10.1073/pnas.1012896107 PMC2973907

[37] W. Xi , H. Yu , Plant Signal. Behav.. 2010, 5, 1315. 20935478 10.4161/psb.5.10.13161 PMC3115377

[38] K. P. Lee , U. Piskurewicz , V. Turečková , M. Strnad , L. Lopez‐Molina , Proc. Natl. Acad. Sci. USA 2010, 107, 19108. 20956298 10.1073/pnas.1012896107 PMC2973907

[39] M. d. C. Rodríguez‐Gacio , M. A. Matilla‐Vázquez , A. J. Matilla , Plant Signal. Behav.. 2009, 4, 1035. 19875942 10.4161/psb.4.11.9902 PMC2819511

[40] M. Okamoto , A. Kuwahara , M. Seo , T. Kushiro , T. Asami , N. Hirai , Y. Kamiya , T. Koshiba , E. Nambara , Plant Physiol. 2006, 141, 97. 16543410 10.1104/pp.106.079475 PMC1459320

[41] W. Jiang , D. Yu , BMC Plant Biol. 2009, 9, 96. 19622176 10.1186/1471-2229-9-96 PMC2719644

[42] K. Shu , X.‐d. Liu , Q. Xie , Z.‐h. He , Mol. Plant 2016, 9, 34. 26343970 10.1016/j.molp.2015.08.010

[43] J. M. Harris , Plants 2015, 4, 548.27135341

[44] H. Takatsuka , M. Umeda , J. Exp. Bot. 2014, 65, 2633. 24474807 10.1093/jxb/ert485

[45] B. Belda‐Palazon , M.‐P. Gonzalez‐Garcia , J. Lozano‐Juste , A. Coego , R. Antoni , J. Julian , M. Peirats‐Llobet , L. Rodriguez , A. Berbel , D. Dietrich , M. A. Fernandez , F. Madueño , M. J. Bennett , P. L. Rodriguez , Proc. Natl. Acad. Sci. USA 2018, 115, E11857. 30482863 10.1073/pnas.1815410115 PMC6294950

[46] L. R. Sun , Y. B. Wang , S. B. He , F. S. Hao , Plant Signal. Behav. 2018, 13, e1500069. 30081737 10.1080/15592324.2018.1500069 PMC6204825

[47] T. Yoshida , A. Christmann , K. Yamaguchi‐Shinozaki , E. Grill , A. R. Fernie , Trends Plant Sci. 2019, 24, 625. 31153771 10.1016/j.tplants.2019.04.008

[48] T. Kuromori , M. Seo , K. Shinozaki , Trends Plant Sci. 2018, 23, 513. 29731225 10.1016/j.tplants.2018.04.001

[49] Y. Zhao , Z. Chan , J. Gao , L. Xing , M. Cao , C. Yu , Y. Hu , J. You , H. Shi , Y. Zhu , Y. Gong , Z. Mu , H. Wang , X. Deng , P. Wang , R. A. Bressan , J.‐K. Zhu , Proc. Natl. Acad. Sci. USA 2016, 113, 1949. 26831097 10.1073/pnas.1522840113 PMC4763734

[50] J. H. Kim , H. R. Woo , J. Kim , P. O. Lim , I. C. Lee , S. H. Choi , D. Hwang , H. G. Nam , Science 2009, 323, 1053. 19229035 10.1126/science.1166386

[51] S. Balazadeh , M. Kwasniewski , C. Caldana , M. Mehrnia , M. I. Zanor , G.‐P. Xue , B. Mueller‐Roeber , Mol. Plant 2011, 4, 346. 21303842 10.1093/mp/ssq080 PMC3063519

[52] Y. Guo , S. Gan , Plant J. 2006, 46, 601. 16640597 10.1111/j.1365-313X.2006.02723.x

[53] Y. Sakuraba , J. Jeong , M.‐Y. Kang , J. Kim , N.‐C. Paek , G. Choi , Nat. Commun. 2014, 5, 4636. 25119965 10.1038/ncomms5636

[54] S. Jan , N. Abbas , M. Ashraf , P. Ahmad , Protoplasma 2019, 256, 313. 30311054 10.1007/s00709-018-1310-5

[55] S. Gao , J. Gao , X. Zhu , Y. Song , Z. Li , G. Ren , X. Zhou , B. Kuai , Mol. Plant 2016, 9, 1272. 27373216 10.1016/j.molp.2016.06.006

[56] Z. Tabaeizadeh , in International Review of Cytology, Vol. 182 (Ed: Jeon K. W.), Academic Press, San Diego, CA 1998, p. 193. 9522461 10.1016/s0074-7696(08)62170-1

[57] W.‐H. Cheng , A. Endo , L. Zhou , J. Penney , H.‐C. Chen , A. Arroyo , P. Leon , E. Nambara , T. Asami , M. Seo , T. Koshiba , J. Sheen , Plant Cell 2002, 14, 2723. 12417697 10.1105/tpc.006494 PMC152723

[58] A. Endo , Y. Sawada , H. Takahashi , M. Okamoto , K. Ikegami , H. Koiwai , M. Seo , T. Toyomasu , W. Mitsuhashi , K. Shinozaki , M. Nakazono , Y. Kamiya , T. Koshiba , E. Nambara , Plant Physiol. 2008, 147, 1984. 18550687 10.1104/pp.108.116632 PMC2492653

[59] H. Bauer , P. Ache , S. Lautner , J. Fromm , W. Hartung , K. A. S. Al‐Rasheid , S. Sonnewald , U. Sonnewald , S. Kneitz , N. Lachmann , R. R. Mendel , F. Bittner , A. M. Hetherington , R. Hedrich , Curr. Biol. 2013, 23, 53. 23219726 10.1016/j.cub.2012.11.022

[60] D. P. Schachtman , J. Q. D. Goodger , Trends Plant Sci. 2008, 13, 281. 18467158 10.1016/j.tplants.2008.04.003

[61] A. Christmann , T. Hoffmann , I. Teplova , E. Grill , A. Müller , Plant Physiol. 2005, 137, 209. 15618419 10.1104/pp.104.053082 PMC548852

[62] A. Daszkowska‐Golec , I. Szarejko , Front. Plant Sci. 2013, 4, 138. 23717320 10.3389/fpls.2013.00138 PMC3652521

[63] B. Brandt , S. Munemasa , C. Wang , D. Nguyen , T. Yong , P. G. Yang , E. Poretsky , T. F. Belknap , R. Waadt , F. Alemán , J. I. Schroeder , eLife 2015, 4, e03599. 10.7554/eLife.03599 PMC4507714 26192964

[64] S. Merlot , N. Leonhardt , F. Fenzi , C. Valon , M. Costa , L. Piette , A. Vavasseur , B. Genty , K. Boivin , A. Müller , J. Giraudat , J. Leung , EMBO J. 2007, 26, 3216. 17557075 10.1038/sj.emboj.7601750 PMC1914098

[65] A. Grondin , O. Rodrigues , L. Verdoucq , S. Merlot , N. Leonhardt , C. Maurel , Plant Cell 2015, 27, 1945. 26163575 10.1105/tpc.15.00421 PMC4531361

[66] M. Jezek , M. R. Blatt , Plant Physiol. 2017, 174, 487. 28408539 10.1104/pp.16.01949 PMC5462021

[67] S. Munemasa , F. Hauser , J. Park , R. Waadt , B. Brandt , J. I. Schroeder , Curr. Opin. Plant Biol. 2015, 28, 154. 26599955 10.1016/j.pbi.2015.10.010 PMC4679528

[68] G. Zeller , S. R. Henz , C. K. Widmer , T. Sachsenberg , G. Rätsch , D. Weigel , S. Laubinger , Plant J. 2009, 58, 1068. 19222804 10.1111/j.1365-313X.2009.03835.x

[69] Y. Fujita , K. Nakashima , T. Yoshida , T. Katagiri , S. Kidokoro , N. Kanamori , T. Umezawa , M. Fujita , K. Maruyama , K. Ishiyama , M. Kobayashi , S. Nakasone , K. Yamada , T. Ito , K. Shinozaki , K. Yamaguchi‐Shinozaki , Plant Cell Physiol. 2009, 50, 2123. 19880399 10.1093/pcp/pcp147

[70] R. Munns , Plant Cell Environ. 2002, 25, 239. 11841667 10.1046/j.0016-8025.2001.00808.x

[71] D. L. Sparks , in Environmental Soil Chemistry, 2nd ed. (Ed: Sparks D. L.), Academic Press, Burlington 2003, p. 285.

[72] P. Parihar , S. Singh , R. Singh , V. P. Singh , S. M. Prasad , Environ. Sci. Pollut. Res. 2015, 22, 4056. 10.1007/s11356-014-3739-1 25398215

[73] S. V. Isayenkov , F. J. M. Maathuis , Front. Plant Sci. 2019, 10, 80. 30828339 10.3389/fpls.2019.00080 PMC6384275

[74] B. Gupta , B. Huang , Int. J. Genomics 2014, 2014, 1. 10.1155/2014/701596 PMC3996477 24804192

[75] R. Hedrich , S. Shabala , Curr. Opin. Plant Biol. 2018, 46, 87. 30138845 10.1016/j.pbi.2018.07.015

[76] A. Sharma , B. Shahzad , V. Kumar , S. K. Kohli , G. P. S. Sidhu , A. S. Bali , N. Handa , D. Kapoor , R. Bhardwaj , B. Zheng , Biomolecules 2019, 9, 285. 10.3390/biom9070285 PMC6680914 31319576

[77] W. Qiao , C. Li , L.‐M. Fan , Environ. Exp. Bot. 2014, 100, 84.

[78] M. Uemura , R. A. Joseph , P. L. Steponkus , Plant Physiol. 1995, 109, 15. 12228580 10.1104/pp.109.1.15 PMC157560

[79] T. Hirayama , K. Shinozaki , Plant J. 2010, 61, 1041. 20409277 10.1111/j.1365-313X.2010.04124.x

[80] Y. Shi , S. Yang , in Abscisic Acid: Metabolism, Transport and Signaling (Ed: Zhang D.‐P.), Springer, Netherlands, Dordrecht 2014, p. 337.

[81] T. Yamada , K. Kuroda , Y. Jitsuyama , D. Takezawa , K. Arakawa , S. Fujikawa , Planta 2002, 215, 770. 12244442 10.1007/s00425-002-0814-5

[82] L. Xiong , M. Ishitani , H. Lee , J. K. Zhu , Plant Cell 2001, 13, 2063. 11549764 10.1105/TPC.010101 PMC139452

[83] H. I. Choi , J. H. Hong , J. O. Ha , J.‐Y. Kang , S. Y. Kim , J. Biol. Chem. 2000, 275, 1723. 10636868 10.1074/jbc.275.3.1723

[84] P. J. Seo , F. Xiang , M. Qiao , J.‐Y. Park , Y. N. Lee , S.‐G. Kim , Y.‐H. Lee , W. J. Park , C.‐M. Park , Plant Physiol. 2009, 151, 275. 19625633 10.1104/pp.109.144220 PMC2735973

[85] X. Dai , Y. Xu , Q. Ma , W. Xu , T. Wang , Y. Xue , K. Chong , Plant Physiol. 2007, 143, 1739. 17293435 10.1104/pp.106.094532 PMC1851822

[86] K.‐N. Kim , Y. H. Cheong , J. J. Grant , G. K. Pandey , S. Luan , Plant Cell 2003, 15, 411. 12566581 10.1105/tpc.006858 PMC141210

[87] L. Xiong , K. S. Schumaker , J.‐K. Zhu , Plant Cell 2002, 14, S165. 12045276 10.1105/tpc.000596 PMC151254

[88] F. Y. Cao , K. Yoshioka , D. Desveaux , J. Plant Res. 2011, 124, 489. 21380629 10.1007/s10265-011-0409-y

[89] F. Y. Cao , K. Yoshioka , D. Desveaux , J. Plant Res. 2011, 124, 489. 21380629 10.1007/s10265-011-0409-y

[90] C. Lim , W. Baek , J. Jung , J.‐H. Kim , S. Lee , Int. J. Mol. Sci. 2015, 16, 15251. 26154766 10.3390/ijms160715251 PMC4519898

[91] P. Vidhyasekaran , in Plant Hormone Signaling Systems in Plant Innate Immunity, Springer, Netherlands, Dordrecht 2015, p. 245.

[92] R. L. Guimarães , H. U. Stotz , Plant Physiol. 2004, 136, 3703. 15502012 10.1104/pp.104.049650 PMC527168

[93] M. de Torres‐Zabala , W. Truman , M. H. Bennett , G. Lafforgue , J. W. Mansfield , P. Rodriguez Egea , L. Bögre , M. Grant , EMBO J. 2007, 26, 1434. 17304219 10.1038/sj.emboj.7601575 PMC1817624

[94] A. Sánchez‐Vallet , G. López , B. Ramos , M. Delgado‐Cerezo , M.‐P. Riviere , F. Llorente , P. V. Fernández , E. Miedes , J. M. Estevez , M. Grant , A. Molina , Plant Physiol. 2012, 160, 2109. 23037505 10.1104/pp.112.200154 PMC3510135

[95] R. Rucińska‐Sobkowiak , Acta Physiol. Plant. 2016, 38, 257.

[96] S. K. Yadav , S. Afr. J. Bot. 2010, 76, 167.

[97] W. Maksymiec , Acta Physiol. Plant. 2007, 29, 177.

[98] L. Bücker‐Neto , A. L. S. Paiva , R. D. Machado , R. A. Arenhart , M. Margis‐Pinheiro , Genet. Mol. Biol. 2017, 40, 373. 28399194 10.1590/1678-4685-GMB-2016-0087 PMC5452142

[99] B. G. Tamang , T. Fukao , Int. J. Mol. Sci. 2015, 16, 30164. 26694376 10.3390/ijms161226226 PMC4691168

[100] T. Fukao , J. Bailey‐Serres , Plant Sci. 2008, 175, 43. 10.1073/pnas.0807821105 PMC2575502 18936491

[101] M. B. Jackson , P. C. Ram , Ann. Bot. 2003, 91, 227. 12509343 10.1093/aob/mcf242 PMC4244997

[102] S.‐H. Yang , D. Choi , Biochem. Biophys. Res. Commun. 2006, 350, 685. 17022939 10.1016/j.bbrc.2006.09.098

[103] H. Saika , M. Okamoto , K. Miyoshi , T. Kushiro , S. Shinoda , Y. Jikumaru , M. Fujimoto , T. Arikawa , H. Takahashi , M. Ando , S.‐i. Arimura , A. Miyao , H. Hirochika , Y. Kamiya , N. Tsutsumi , E. Nambara , M. Nakazono , Plant Cell Physiol. 2007, 48, 287. 17205969 10.1093/pcp/pcm003

[104] T. Fukao , J. Bailey‐Serres , Proc. Natl. Acad. Sci. USA 2008, 105, 16814. 18936491 10.1073/pnas.0807821105 PMC2575502

[105] R. Müller‐Xing , Q. Xing , J. Goodrich , Front. Plant. Sci. 2014, 5, 474. 25278950 10.3389/fpls.2014.00474 PMC4165212

[106] S. A.‐H. Mackerness , Plant Growth Regul. 2000, 32, 27.

[107] Z. Xie , Y. Wang , Y. Liu , Y. Liu , Eur. J. Soil Biol. 2009, 45, 377.

[108] G. I. Jenkins , Annu. Rev. Plant Biol. 2009, 60, 407. 19400728 10.1146/annurev.arplant.59.032607.092953

[109] M. H. Sangtarash , M. M. Qaderi , C. C. Chinnappa , D. M. Reid , Environ. Exp. Bot. 2009, 66, 212.

[110] V. Tossi , R. Cassia , S. Bruzzone , E. Zocchi , L. Lamattina , Trends Plant Sci. 2012, 17, 510. 22698377 10.1016/j.tplants.2012.05.007

[111] V. Tossi , L. Lamattina , R. Cassia , New Phytol. 2009, 181, 871. 19140950 10.1111/j.1469-8137.2008.02722.x

[112] A. Kageyama , K. Ishizaki , T. Kohchi , H. Matsuura , K. Takahashi , Phytochemistry 2015, 117, 547. 26055979 10.1016/j.phytochem.2015.05.009

[113] E. Guajardo , J. A. Correa , L. Contreras‐Porcia , Planta 2016, 243, 767. 26687373 10.1007/s00425-015-2438-6

[114] L. Xiong , J.‐K. Zhu , Plant Physiol. 2003, 133, 29. 12970472 10.1104/pp.103.025395 PMC523868

[115] M. Okazaki , M. Kittikorn , K. Ueno , M. Mizutani , N. Hirai , S. Kondo , T. Ohnishi , Y. Todoroki , Bioorg. Med. Chem. 2012, 20, 3162. 22525496 10.1016/j.bmc.2012.03.068

[116] F. Bouvier , A. D'Harlingue , R. A. Backhaus , M. H. Kumagai , B. Camara , Eur. J. Biochem. 2000, 267, 6346. 11029576 10.1046/j.1432-1327.2000.01722.x

[117] M. Seo , T. Koshiba , Trends Plant Sci. 2002, 7, 41. 11804826 10.1016/s1360-1385(01)02187-2

[118] S. H. Schwartz , J. A. D. Zeevaart , in Plant Hormones: Biosynthesis, Signal Transduction, Action! (Ed: Davies P. J.), Springer, Netherlands, Dordrecht 2010, p. 137.

[119] E. Nambara , A. Marion‐Poll , Annu. Rev. Plant Biol. 2005, 56, 165. 15862093 10.1146/annurev.arplant.56.032604.144046

[120] C. D. Rock , J. A. Zeevaart , Proc. Natl. Acad. Sci. USA 1991, 88, 7496. 11607209 10.1073/pnas.88.17.7496 PMC52327

[121] A. M. Jones , New Phytol. 2016, 210, 38. 26201893 10.1111/nph.13552

[122] A. Endo , M. Okamoto , T. Koshiba , in Abscisic Acid: Metabolism, Transport and Signaling (Ed: Zhang D.‐P.), Springer, Dordrecht 2014.

[123] N. Nishimura , K. Hitomi , A. S. Arvai , R. P. Rambo , C. Hitomi , S. R. Cutler , J. I. Schroeder , E. D. Getzoff , Science 2009, 326, 1373. 19933100 10.1126/science.1181829 PMC2835493

[124] H. M. North , A. De Almeida , J. P. Boutin , A. Frey , A. To , L. Botran , B. Sotta , A. Marion‐Poll , Plant J. 2007, 50, 810. 17470058 10.1111/j.1365-313X.2007.03094.x

[125] S. H. Schwartz , B. C. Tan , D. A. Gage , J. A. Zeevaart , D. R. McCarty , Science 1997, 276, 1872. 9188535 10.1126/science.276.5320.1872

[126] S. Iuchi , M. Kobayashi , T. Taji , M. Naramoto , M. Seki , T. Kato , S. Tabata , Y. Kakubari , K. Yamaguchi‐Shinozaki , K. Shinozaki , Plant J. 2001, 27, 325. 11532178 10.1046/j.1365-313x.2001.01096.x

[127] S. H. Schwartz , X. Qin , J. A. Zeevaart , Plant Physiol. 2003, 131, 1591. 12692318 10.1104/pp.102.017921 PMC1540303

[128] F. R. Finkelstein , in The Arabidopsis Book, Vol. 11 (Ed: Torii K.), American Society of Plant Biologists, Rockville, MD 2013, p. e0166.24273463

[129] B. C. Tan , L. M. Joseph , W. T. Deng , L. Liu , Q. B. Li , K. Cline , D. R. McCarty , Plant J. 2003, 35, 44. 12834401 10.1046/j.1365-313x.2003.01786.x

[130] S. Al‐Babili , P. Hugueney , M. Schledz , R. Welsch , H. Frohnmeyer , O. Laule , P. Beyer , FEBS Lett. 2000, 485, 168. 11094161 10.1016/s0014-5793(00)02193-1

[131] G. Ronen , L. Carmel‐Goren , D. Zamir , J. Hirschberg , Proc. Natl. Acad. Sci. USA 2000, 97, 11102. 10995464 10.1073/pnas.190177497 PMC27155

[132] M. Seo , A. J. Peeters , H. Koiwai , T. Oritani , A. Marion‐Poll , J. A. Zeevaart , M. Koornneef , Y. Kamiya , T. Koshiba , Proc. Natl. Acad. Sci. USA 2000, 97, 12908. 11050171 10.1073/pnas.220426197 PMC18863

[133] S.‐Y. Han , N. Kitahata , K. Sekimata , T. Saito , M. Kobayashi , K. Nakashima , K. Yamaguchi‐Shinozaki , K. Shinozaki , S. Yoshida , T. Asami , Plant Physiol. 2004, 135, 1574. 15247398 10.1104/pp.104.039511 PMC519072

[134] H. E. Blackwell , Y. Zhao , Plant Physiol. 2003, 133, 448. 14555772 10.1104/pp.103.031138 PMC1540336

[135] T. Asami , T. Nakano , H. Nakashita , K. Sekimata , Y. Shimada , S. Yoshida , J. Plant Growth Regul. 2003, 22, 336. 14676973 10.1007/s00344-003-0065-0

[136] Y. Zhao , X. Dai , H. E. Blackwell , S. L. Schreiber , J. Chory , Science 2003, 301, 1107. 12893885 10.1126/science.1084161

[137] K. Gallardo , C. Job , S. P. Groot , M. Puype , H. Demol , J. Vandekerckhove , D. Job , Plant Physiol. 2002, 129, 823. 12068122 10.1104/pp.002816 PMC161704

[138] Z. Y. Wang , T. Nakano , J. Gendron , J. He , M. Chen , D. Vafeados , Y. Yang , S. Fujioka , S. Yoshida , T. Asami , J. Chory , Dev. Cell 2002, 2, 505. 11970900 10.1016/s1534-5807(02)00153-3

[139] N. Nishimura , T. Yoshida , M. Murayama , T. Asami , K. Shinozaki , T. Hirayama , Plant Cell Physiol. 2004, 45, 1485. 15564532 10.1093/pcp/pch171

[140] N. Nishimura , N. Kitahata , M. Seki , Y. Narusaka , M. Narusaka , T. Kuromori , T. Asami , K. Shinozaki , T. Hirayama , Plant J. 2005, 44, 972. 16359390 10.1111/j.1365-313X.2005.02589.x

[141] T. Yoshida , N. Nishimura , N. Kitahata , T. Kuromori , T. Ito , T. Asami , K. Shinozaki , T. Hirayama , Plant Physiol. 2006, 140, 115. 16339800 10.1104/pp.105.070128 PMC1326036

[142] N. Kitahata , S.‐Y. Han , N. Noji , T. Saito , M. Kobayashi , T. Nakano , K. Kuchitsu , K. Shinozaki , S. Yoshida , S. Matsumoto , M. Tsujimoto , T. Asami , Bioorg. Med. Chem. 2006, 14, 5555. 16682205 10.1016/j.bmc.2006.04.025

[143] J. Boyd , Y. Gai , K. M. Nelson , E. Lukiwski , J. Talbot , M. K. Loewen , S. Owen , L. Irina Zaharia , A. J. Cutler , S. R. Abrams , M. C. Loewen , Bioorg. Med. Chem. 2009, 17, 2902. 19269833 10.1016/j.bmc.2009.01.076

[144] D. Latowski , A. K. Banaś , K. Strzałka , H. Gabryś , J. Plant Physiol. 2007, 164, 231. 17074410 10.1016/j.jplph.2006.09.003

[145] D. Latowski , J. Kruk , K. Burda , M. Skrzynecka‐Jaskier , A. Kostecka‐Gugała , K. Strzałka , Eur. J. Biochem. 2002, 269, 4656. 12230579 10.1046/j.1432-1033.2002.03166.x

[146] H. Y. Yamamoto , L. Kamite , Biochim. Biophys. Acta (BBA) ‐ Bioenerg. 1972, 267, 538. 10.1016/0005-2728(72)90182-x 5047136

[147] P. Jahns , D. Latowski , K. Strzalka , Biochim. Biophys. Acta (BBA) ‐ Bioenerg. 2009, 1787, 3. 10.1016/j.bbabio.2008.09.013 18976630

[148] P. Müller‐Moulé , P. L. Conklin , K. K. Niyogi , Plant Physiol. 2002, 128, 970. 11891252 10.1104/pp.010924 PMC152209

[149] E. A. Havir , S. L. Tausta , R. B. Peterson , Plant Sci. 1997, 123, 57.

[150] S. M. Norman , R. D. Bennett , V. P. Maier , S. M. Poling , Plant Sci. Lett. 1983, 28, 255.

[151] S. M. Norman , R. D. Bennett , S. M. Poling , V. P. Maier , M. D. Nelson , Plant Physiol. 1986, 80, 122. 16664565 10.1104/pp.80.1.122 PMC1075067

[152] R. C. Coolbaugh , D. I. Swanson , C. A. West , Plant Physiol. 1982, 69, 707. 16662280 10.1104/pp.69.3.707 PMC426285

[153] P. E. Gamble , J. E. Mullet , Eur. J. Biochem. 1986, 160, 117. 2945718 10.1111/j.1432-1033.1986.tb09947.x

[154] S. J. Neill , R. Horgan , A. D. Parry , Planta 1986, 169, 87. 24232433 10.1007/BF01369779

[155] R. A. Creelman , E. Bell , J. E. Mullet , Plant Physiol. 1992, 99, 1258. 16668998 10.1104/pp.99.3.1258 PMC1080612

[156] P. Merigout , F. Kepes , A. M. Perret , B. Satiat‐Jeunemaitre , P. Moreau , FEBS Lett. 2002, 518, 88. 11997023 10.1016/s0014-5793(02)02651-0

[157] S. M. Norman , S. M. Poling , V. P. Maier , E. D. Orme , Plant Physiol. 1983, 71, 15. 16662775 10.1104/pp.71.1.15 PMC1065977

[158] Y. Kanno , A. Hanada , Y. Chiba , T. Ichikawa , M. Nakazawa , M. Matsui , T. Koshiba , Y. Kamiya , M. Seo , Proc. Natl. Acad. Sci. USA 2012, 109, 9653. 22645333 10.1073/pnas.1203567109 PMC3386071

[159] Y. Boursiac , S. Léran , C. Corratgé‐Faillie , A. Gojon , G. Krouk , B. Lacombe , Trends Plant Sci. 2013, 18, 325. 23453706 10.1016/j.tplants.2013.01.007

[160] Y. Chiba , T. Shimizu , S. Miyakawa , Y. Kanno , T. Koshiba , Y. Kamiya , M. Seo , J. Plant Res. 2015, 128, 679. 25801271 10.1007/s10265-015-0710-2

[161] S. Léran , K. Varala , J.‐C. Boyer , M. Chiurazzi , N. Crawford , F. Daniel‐Vedele , L. David , R. Dickstein , E. Fernandez , B. Forde , W. Gassmann , D. Geiger , A. Gojon , J.‐M. Gong , B. A. Halkier , J. M. Harris , R. Hedrich , A. M. Limami , D. Rentsch , M. Seo , Y.‐F. Tsay , M. Zhang , G. Coruzzi , B. Lacombe , Trends Plant Sci. 2014, 19, 5. 24055139 10.1016/j.tplants.2013.08.008

[162] C. Corratgé‐Faillie , B. Lacombe , J. Exp. Bot. 2017, 68, 3107. 28186545 10.1093/jxb/erw499

[163] X. Fan , M. Naz , X. Fan , W. Xuan , A. J. Miller , G. Xu , J. Exp. Bot. 2017, 68, 2463. 28158856 10.1093/jxb/erx011

[164] J. Kang , J.‐U. Hwang , M. Lee , Y.‐Y. Kim , S. M. Assmann , E. Martinoia , Y. Lee , Proc. Natl. Acad. Sci. USA 2010, 107, 2355. 20133880 10.1073/pnas.0909222107 PMC2836657

[165] G. Andolfo , M. Ruocco , A. Di Donato , L. Frusciante , M. Lorito , F. Scala , M. R. Ercolano , BMC Plant Biol. 2015, 15, 51. 25850033 10.1186/s12870-014-0323-2 PMC4358917

[166] J. Kang , S. Yim , H. Choi , A. Kim , K. P. Lee , L. Lopez‐Molina , E. Martinoia , Y. Lee , Nat. Commun. 2015, 6, 8113. 26334616 10.1038/ncomms9113 PMC4569717

[167] H. Zhang , H. Zhu , Y. Pan , Y. Yu , S. Luan , L. Li , Mol. Plant 2014, 7, 1522. 24851876 10.1093/mp/ssu063

[168] T. Kuromori , T. Miyaji , H. Yabuuchi , H. Shimizu , E. Sugimoto , A. Kamiya , Y. Moriyama , K. Shinozaki , Proc. Natl. Acad. Sci. USA 2010, 107, 2361. 20133881 10.1073/pnas.0912516107 PMC2836683

[169] T. Kuromori , K. Shinozaki , Plant Signal. Behav.. 2010, 5, 1124. 20935463 10.4161/psb.5.9.12566 PMC3115083

[170] T. Kuromori , E. Sugimoto , K. Shinozaki , Plant J. 2011, 67, 885. 21575091 10.1111/j.1365-313X.2011.04641.x

[171] M. Galbiati , L. Simoni , G. Pavesi , E. Cominelli , P. Francia , A. Vavasseur , T. Nelson , M. Bevan , C. Tonelli , Plant J. 2008, 53, 750. 18036199 10.1111/j.1365-313X.2007.03371.x

[172] H. Choi , K. Ohyama , Y.‐Y. Kim , J.‐Y. Jin , S. B. Lee , Y. Yamaoka , T. Muranaka , M. C. Suh , S. Fujioka , Y. Lee , Plant Cell 2014, 26, 310. 24474628 10.1105/tpc.113.118935 PMC3963578

[173] H. Ji , Y. Peng , N. Meckes , S. Allen , N. Stewart , M. B. Traw , Plant Physiol. 2014, 166, 879. 25146567 10.1104/pp.114.248153 PMC4213115

[174] I. Tal , Y. Zhang , M. E. Jørgensen , O. Pisanty , I. C. R. Barbosa , M. Zourelidou , T. Regnault , C. Crocoll , C. E. Olsen , R. Weinstain , C. Schwechheimer , B. A. Halkier , H. H. Nour‐Eldin , M. Estelle , E. Shani , Nat. Commun. 2016, 7, 11486. 27139299 10.1038/ncomms11486 PMC4857387

[175] N. Leonhardt , A. Vavasseur , C. Forestier , Plant Cell 1999, 11, 1141. 10368184 10.1105/tpc.11.6.1141 PMC144242

[176] N. Leonhardt , E. Marin , A. Vavasseur , C. Forestier , Proc. Natl. Acad. Sci. USA 1997, 94, 14156. 9391169 10.1073/pnas.94.25.14156 PMC28449

[177] P. Klíma , M. Laňková , E. Zažímalová , Protoplasma 2016, 253, 1391. 26494150 10.1007/s00709-015-0897-z

[178] B. Burla , S. Pfrunder , R. Nagy , R. M. Francisco , Y. Lee , E. Martinoia , Plant Physiol. 2013, 163, 1446. 24028845 10.1104/pp.113.222547 PMC3813663

[179] A. Schweighofer , H. Hirt , I. Meskiene , Trends Plant Sci. 2004, 9, 236. 15130549 10.1016/j.tplants.2004.03.007

[180] C. Máthé , T. Garda , C. Freytag , M. M‐Hamvas , Int. J. Mol. Sci. 2019, 20, 3028. 10.3390/ijms20123028 PMC6628354 31234298

[181] T. Umezawa , K. Nakashima , T. Miyakawa , T. Kuromori , M. Tanokura , K. Shinozaki , K. Yamaguchi‐Shinozaki , Plant Cell Physiol. 2010, 51, 1821. 20980270 10.1093/pcp/pcq156 PMC2978318

[182] A. Amagai , Y. Honda , S. Ishikawa , Y. Hara , M. Kuwamura , A. Shinozawa , N. Sugiyama , Y. Ishihama , D. Takezawa , Y. Sakata , K. Shinozaki , T. Umezawa , Plant J. 2018, 94, 699. 29575231 10.1111/tpj.13891

[183] J. Guo , X. Yang , D. J. Weston , J.‐G. Chen , J. Integr. Plant Biol. 2011, 53, 469. 21554537 10.1111/j.1744-7909.2011.01044.x

[184] M. Gonzalez‐Guzman , G. A. Pizzio , R. Antoni , F. Vera‐Sirera , E. Merilo , G. W. Bassel , M. A. Fernández , M. J. Holdsworth , M. A. Perez‐Amador , H. Kollist , P. L. Rodriguez , Plant Cell 2012, 24, 2483. 22739828 10.1105/tpc.112.098574 PMC3406898

[185] J. Santiago , F. Dupeux , K. Betz , R. Antoni , M. Gonzalez‐Guzman , L. Rodriguez , J. A. Márquez , P. L. Rodriguez , Plant Sci. 2012, 182, 3. 22118610 10.1016/j.plantsci.2010.11.014

[186] M. Okamoto , F. C. Peterson , A. Defries , S.‐Y. Park , A. Endo , E. Nambara , B. F. Volkman , S. R. Cutler , Proc. Natl. Acad. Sci. USA 2013, 110, 12132. 23818638 10.1073/pnas.1305919110 PMC3718107

[187] Q. Hao , P. Yin , W. Li , L. Wang , C. Yan , Z. Lin , J. Z. Wu , J. Wang , S. F. Yan , N. Yan , Mol. Cell 2011, 42, 662. 21658606 10.1016/j.molcel.2011.05.011

[188] J. Santiago , A. Rodrigues , A. Saez , S. Rubio , R. Antoni , F. Dupeux , S.‐Y. Park , J. A. Márquez , S. R. Cutler , P. L. Rodriguez , Plant J. 2009, 60, 575. 19624469 10.1111/j.1365-313X.2009.03981.x

[189] Y. Takahashi , J. Zhang , P.‐K. Hsu , P. H. O. Ceciliato , L. Zhang , G. Dubeaux , S. Munemasa , C. Ge , Y. Zhao , F. Hauser , J. I. Schroeder , Nat. Commun. 2020, 11, 12. 31896774 10.1038/s41467-019-13875-y PMC6940395

[190] M. Saruhashi , T. Kumar Ghosh , K. Arai , Y. Ishizaki , K. Hagiwara , K. Komatsu , Y. Shiwa , K. Izumikawa , H. Yoshikawa , T. Umezawa , Y. Sakata , D. Takezawa , Proc. Natl. Acad. Sci. USA 2015, 112, E6388. 26540727 10.1073/pnas.1511238112 PMC4655548

[191] S. Katsuta , G. Masuda , H. Bak , A. Shinozawa , Y. Kamiyama , T. Umezawa , D. Takezawa , I. Yotsui , T. Taji , Y. Sakata , Plant J. 2020, 10.1111/tpj.14756. PMC7497244 32239564

[192] K. Melcher , L.‐M. Ng , X. E. Zhou , F.‐F. Soon , Y. Xu , K. M. Suino‐Powell , S.‐Y. Park , J. J. Weiner , H. Fujii , V. Chinnusamy , A. Kovach , J. Li , Y. Wang , J. Li , F. C. Peterson , D. R. Jensen , E.‐L. Yong , B. F. Volkman , S. R. Cutler , J.‐K. Zhu , H. E. Xu , Nature 2009, 462, 602. 19898420 10.1038/nature08613 PMC2810868

[193] M. moreno alvero , C. Yunta , M. Gonzalez‐Guzman , J. Lozano‐Juste , J. Benavente , V. Arbona , M. Menendez , M. Martinez‐Ripoll , L. Infantes , A. Gómez‐Cadenas , P. Rodriguez , A. Albert , Mol. Plant 2017, 10, 1250. 28736053 10.1016/j.molp.2017.07.004

[194] Z. Ren , Z. Wang , X. E. Zhou , H. Shi , Y. Hong , M. Cao , Z. Chan , X. Liu , H. E. Xu , J.‐K. Zhu , Sci. Rep. 2017, 7, 14022. 29070857 10.1038/s41598-017-14101-9 PMC5656587

[195] F. Hauser , Z. Li , R. Waadt , J. I. Schroeder , Cell 2017, 171, 1708. 29245015 10.1016/j.cell.2017.11.045 PMC5895850

[196] J. P. Klingler , G. Batelli , J.‐K. Zhu , J. Exp. Bot. 2010, 61, 3199. 20522527 10.1093/jxb/erq151 PMC3107536

[197] B. Brandt , D. E. Brodsky , S. Xue , J. Negi , K. Iba , J. Kangasjärvi , M. Ghassemian , A. B. Stephan , H. Hu , J. I. Schroeder , Proc. Natl. Acad. Sci. USA 2012, 109, 10593. 22689970 10.1073/pnas.1116590109 PMC3387046

[198] T. Umezawa , N. Sugiyama , M. Mizoguchi , S. Hayashi , F. Myouga , K. Yamaguchi‐Shinozaki , Y. Ishihama , T. Hirayama , K. Shinozaki , Proc. Natl. Acad. Sci. USA 2009, 106, 17588. 19805022 10.1073/pnas.0907095106 PMC2754379

[199] Q. Wu , X. Zhang , M. Peirats‐Llobet , B. Belda‐Palazon , X. Wang , S. Cui , X. Yu , P. L. Rodriguez , C. An , Plant Cell 2016, 28, 2178. 27577789 10.1105/tpc.16.00364 PMC5059804

[200] S. Fuchs , S. V. Tischer , C. Wunschel , A. Christmann , E. Grill , Proc. Natl. Acad. Sci. USA 2014, 111, 5741. 24706923 10.1073/pnas.1322085111 PMC3992651

[201] Y. Sun , B. Harpazi , A. Wijerathna‐Yapa , E. Merilo , J. de Vries , D. Michaeli , M. Gal , A. C. Cuming , H. Kollist , A. Mosquna , Proc. Natl. Acad. Sci. USA 2019, 116, 24892. 31744875 10.1073/pnas.1914480116 PMC6900503

[202] J.‐F. Yang , M.‐X. Chen , J.‐H. Zhang , G.‐F. Hao , G.‐F. Yang , J. Exp. Bot. 2020, 71, 1322. 31740933 10.1093/jxb/erz511

[203] X. Tian , Z. Wang , X. Li , T. Lv , H. Liu , L. Wang , H. Niu , Q. Bu , Rice (New York, N.Y.) 2015, 8, 28. 10.1186/s12284-015-0061-6 PMC4567572 26362328

[204] S. Han , Y. Lee , E. J. Park , M. K. Min , Y. Lee , T.‐H. Kim , B.‐G. Kim , S. Lee , Plant Mol. Biol. 2019, 100, 319. 30941543 10.1007/s11103-019-00862-6

[205] M. González‐Guzmán , L. Rodríguez , L. Lorenzo‐Orts , C. Pons , A. Sarrión‐Perdigones , M. A. Fernández , M. Peirats‐Llobet , J. Forment , M. Moreno‐Alvero , S. R. Cutler , A. Albert , A. Granell , P. L. Rodríguez , J. Exp. Bot. 2014, 65, 4451. 24863435 10.1093/jxb/eru219 PMC4112642

[206] G. Bai , H. Xie , H. Yao , F. Li , X. Chen , Y. Zhang , B. Xiao , J. Yang , Y. Li , D.‐H. Yang , BMC Genomics 2019, 20, 575. 31296158 10.1186/s12864-019-5839-2 PMC6625023

[207] G. Bai , D.‐H. Yang , Y. Zhao , S. Ha , F. Yang , J. Ma , X.‐S. Gao , Z.‐M. Wang , J.‐K. Zhu , Plant Mol. Biol. 2013, 83, 651. 23934343 10.1007/s11103-013-0114-4 PMC3834219

[208] C. Li , H. Jia , Y. Chai , Y. Shen , Plant Signal. Behav.. 2011, 6, 1950. 22095148 10.4161/psb.6.12.18024 PMC3337185

[209] A. Mosquna , F. C. Peterson , S.‐Y. Park , J. Lozano‐Juste , B. F. Volkman , S. R. Cutler , Proc. Natl. Acad. Sci. USA 2011, 108, 20838. 22139369 10.1073/pnas.1112838108 PMC3251050

[210] K.‐i. Miyazono , T. Miyakawa , Y. Sawano , K. Kubota , H.‐J. Kang , A. Asano , Y. Miyauchi , M. Takahashi , Y. Zhi , Y. Fujita , T. Yoshida , K.‐S. Kodaira , K. Yamaguchi‐Shinozaki , M. Tanokura , Nature 2009, 462, 609. 19855379 10.1038/nature08583

[211] P. Yin , H. Fan , Q. Hao , X. Yuan , D. Wu , Y. Pang , C. Yan , W. Li , J. Wang , N. Yan , Nat. Struct. Mol. Biol. 2009, 16, 1230. 19893533 10.1038/nsmb.1730

[212] Z. Yang , J. Liu , S. V. Tischer , A. Christmann , W. Windisch , H. Schnyder , E. Grill , Proc. Natl. Acad. Sci. USA 2016, 113, 6791. 27247417 10.1073/pnas.1601954113 PMC4914173

[213] X. Li , G. Li , Y. Li , X. Kong , L. Zhang , J. Wang , X. Li , Y. Yang , Int. J. Mol. Sci. 2018, 19, 1938.

[214] X. Zhang , Q. Zhang , Q. Xin , L. Yu , Z. Wang , W. Wu , L. Jiang , G. Wang , W. Tian , Z. Deng , Y. Wang , Z. Liu , J. Long , Z. Gong , Z. Chen , Structure 2012, 20, 780. 22579247 10.1016/j.str.2012.02.019

[215] X. Zhang , L. Jiang , G. Wang , L. Yu , Q. Zhang , Q. Xin , W. Wu , Z. Gong , Z. Chen , PLoS One 2013, 8, e67477. 23844015 10.1371/journal.pone.0067477 PMC3699650

[216] W. Li , L. Wang , X. Sheng , C. Yan , R. Zhou , J. Hang , P. Yin , N. Yan , Cell Res. 2013, 23, 1369. 24165892 10.1038/cr.2013.143 PMC3847573

[217] Q. Hao , P. Yin , C. Yan , X. Yuan , W. Li , Z. Zhang , L. Liu , J. Wang , N. Yan , J. Biol. Chem. 2010, 285, 28946. 20554531 10.1074/jbc.M110.149005 PMC2937921

[218] C. L. Benson , M. Kepka , C. Wunschel , N. Rajagopalan , K. M. Nelson , A. Christmann , S. R. Abrams , E. Grill , M. C. Loewen , Phytochemistry 2015, 113, 96. 24726371 10.1016/j.phytochem.2014.03.017

[219] I. Szostkiewicz , K. Richter , M. Kepka , S. Demmel , Y. Ma , A. Korte , F. F. Assaad , A. Christmann , E. Grill , Plant J. 2010, 61, 25. 19769575 10.1111/j.1365-313X.2009.04025.x

[220] F. Dupeux , R. Antoni , K. Betz , J. Santiago , M. Gonzalez‐Guzman , L. Rodriguez , S. Rubio , S.‐Y. Park , S. R. Cutler , P. L. Rodriguez , J. A. Márquez , Plant Physiol. 2011, 156, 106. 21357183 10.1104/pp.110.170894 PMC3091035

[221] X. Yuan , P. Yin , Q. Hao , C. Yan , J. Wang , N. Yan , J. Biol. Chem. 2010, 285, 28953. 20630864 10.1074/jbc.M110.160192 PMC2937922

[222] J.‐F. Yang , C.‐Y. Yin , D. Wang , C.‐Y. Jia , G.‐F. Hao , G.‐F. Yang , Front. Chem. 2020, 8, 425. 32582630 10.3389/fchem.2020.00425 PMC7287503

[223] F. C. Peterson , E. S. Burgie , S.‐Y. Park , D. R. Jensen , J. J. Weiner , C. A. Bingman , C.‐E. A. Chang , S. R. Cutler , G. N. Phillips Jr. , B. F. Volkman , Nat. Struct. Mol. Biol. 2010, 17, 1109. 20729860 10.1038/nsmb.1898 PMC2933299

[224] M.‐J. Cao , Y.‐L. Zhang , X. Liu , H. Huang , X. E. Zhou , W.‐L. Wang , A. Zeng , C.‐Z. Zhao , T. Si , J. Du , W.‐W. Wu , F.‐X. Wang , H. E. Xu , J.‐K. Zhu , Nat. Commun. 2017, 8, 1183. 29084945 10.1038/s41467-017-01239-3 PMC5662759

[225] F. Dupeux , J. Santiago , K. Betz , J. Twycross , S.‐Y. Park , L. Rodriguez , M. Gonzalez‐Guzman , M. R. Jensen , N. Krasnogor , M. Blackledge , M. Holdsworth , S. R. Cutler , P. L. Rodriguez , J. A. Márquez , EMBO J. 2011, 30, 4171. 21847091 10.1038/emboj.2011.294 PMC3199383

[226] M. S. Naeem , L. Dai , F. Ahmad , A. Ahmad , J. Li , C. Zhang , Acta Physiol. Plant. 2016, 38, 183.

[227] Z. Cheng , R. Jin , M. Cao , X. Liu , Z. Chan , Plant Cell Tiss. Org. Cult. (PCTOC). 2016, 125, 231.

[228] A. S. Vaidya , F. C. Peterson , D. Yarmolinsky , E. Merilo , I. Verstraeten , S.‐Y. Park , D. Elzinga , A. Kaundal , J. Helander , J. Lozano‐Juste , M. Otani , K. Wu , D. R. Jensen , H. Kollist , B. F. Volkman , S. R. Cutler , ACS Chem. Biol. 2017, 12, 2842. 28949512 10.1021/acschembio.7b00650

[229] A. S. Vaidya , J. D. M. Helander , F. C. Peterson , D. Elzinga , W. Dejonghe , A. Kaundal , S.‐Y. Park , Z. Xing , R. Mega , J. Takeuchi , B. Khanderahoo , S. Bishay , B. F. Volkman , Y. Todoroki , M. Okamoto , S. R. Cutler , Science 2019, 366, eaaw8848. 31649167 10.1126/science.aaw8848

[230] S.‐Y. Park , F. C. Peterson , A. Mosquna , J. Yao , B. F. Volkman , S. R. Cutler , Nature 2015, 520, 545. 25652827 10.1038/nature14123

[231] P. L. Rodriguez , J. Lozano‐Juste , Trends Plant Sci. 2015, 20, 330. 25891067 10.1016/j.tplants.2015.04.001

[232] Y. Todoroki , N. Hirai , K. Koshimizu , Phytochemistry 1995, 38, 561.

[233] M. L. Windsor , J. A. D. Zeevaart , Phytochemistry 1997, 45, 931. 9214776 10.1016/s0031-9422(97)00022-8

[234] J. Takeuchi , T. Ohnishi , M. Okamoto , Y. Todoroki , Bioorg. Med. Chem. Lett. 2015, 25, 3507. 26174552 10.1016/j.bmcl.2015.06.088

[235] J. Takeuchi , M. Okamoto , T. Akiyama , T. Muto , S. Yajima , M. Sue , M. Seo , Y. Kanno , T. Kamo , A. Endo , E. Nambara , N. Hirai , T. Ohnishi , S. R. Cutler , Y. Todoroki , Nat. Chem. Biol. 2014, 10, 477. 24792952 10.1038/nchembio.1524

[236] J. Takeuchi , T. Ohnishi , M. Okamoto , Y. Todoroki , Org. Biomol. Chem. 4278, 2015, 13. 25758810 10.1039/c4ob02662d

[237] J. Takeuchi , N. Mimura , M. Okamoto , S. Yajima , M. Sue , T. Akiyama , K. Monda , K. Iba , T. Ohnishi , Y. Todoroki , ACS Chem. Biol. 2018, 13, 1313. 29620349 10.1021/acschembio.8b00105

[238] J. Nyangulu , K. Nelson , P. Rose , Y. Gai , M. Loewen , B. Lougheed , J. Quail , A. Cutler , S. Abrams , Org. Biomol. Chem. 2006, 4, 1400. 16557330 10.1039/b509193d

[239] M. Kepka , C. L. Benson , V. K. Gonugunta , K. M. Nelson , A. Christmann , E. Grill , S. R. Abrams , Plant Physiol. 2011, 157, 2108. 21976481 10.1104/pp.111.182584 PMC3327214

[240] N. Rajagopalan , K. M. Nelson , A. F. Douglas , V. Jheengut , I. Q. Alarcon , S. A. McKenna , M. Surpin , M. C. Loewen , S. R. Abrams , Biochemistry 2016, 55, pp. 5155. 27523384 10.1021/acs.biochem.6b00605

[241] E. M. Hrabak , C. W. M. Chan , M. Gribskov , J. F. Harper , J. H. Choi , N. Halford , J. Kudla , S. Luan , H. G. Nimmo , M. R. Sussman , M. Thomas , K. Walker‐Simmons , J.‐K. Zhu , A. C. Harmon , Plant Physiol. 2003, 132, 666. 12805596 10.1104/pp.102.011999 PMC167006

[242] N. G. Halford , J. P. Boulyz , M. Thomas , in Advances in Botanical Research, Vol. 32, Academic Press, San Diego, CA 2000, p. 405.

[243] Y. Kobayashi , S. Yamamoto , H. Minami , Y. Kagaya , T. Hattori , Plant Cell 2004, 16, 1163. 15084714 10.1105/tpc.019943 PMC423207

[244] M. Boudsocq , C. Laurière , Plant Physiol. 2005, 138, 1185. 16009994 10.1104/pp.105.061275 PMC1176393

[245] H. Fujii , P. E. Verslues , J.‐K. Zhu , Plant Cell 2007, 19, 485. 17307925 10.1105/tpc.106.048538 PMC1867333

[246] R. Yoshida , T. Umezawa , T. Mizoguchi , S. Takahashi , F. Takahashi , K. Shinozaki , J. Biol. Chem. 2006, 281, 5310. 16365038 10.1074/jbc.M509820200

[247] P. Wu , T. E. Nielsen , M. H. Clausen , Trends Pharmacol. Sci. 2015, 36, 422. 25975227 10.1016/j.tips.2015.04.005

[248] Z. M. Pei , K. Kuchitsu , J. M. Ward , M. Schwarz , J. I. Schroeder , Plant Cell 1997, 9, 409. 9090884 10.1105/tpc.9.3.409 PMC156927

[249] M. E. Hoyos , S. Zhang , Plant Physiol. 2000, 122, 1355. 10759532 10.1104/pp.122.4.1355 PMC58971

[250] A. Kelner , I. Pękala , S. Kaczanowski , G. Muszyńska , D. G. Hardie , G. Dobrowolska , Plant Physiol. 2004, 136, 3255. 15466234 10.1104/pp.104.046151 PMC523384

[251] A. de Zelicourt , J. Colcombet , H. Hirt , Trends Plant Sci. 2016, 21, 677. 27143288 10.1016/j.tplants.2016.04.004

[252] M. Rasmussen , M. Roux , M. Petersen , J. Mundy , Front. Plant Sci. 2012, 3, 169. 22837762 10.3389/fpls.2012.00169 PMC3402898

[253] M. L. W. Knetsch , M. Wang , B. E. Snaar‐Jagalska , S. Heimovaara‐Dijkstra , Plant Cell 1996, 8, 1061. 12239411 10.1105/tpc.8.6.1061 PMC161161

[254] E. C. Burnett , R. Desikan , R. C. Moser , S. J. Neill , J. Exp. Bot. 2000, 51, 197. 10938826 10.1093/jexbot/51.343.197

[255] J. Jiang , P. Wang , G. An , P. Wang , C.‐P. Song , Plant Cell Rep. 2008, 27, 377. 17924117 10.1007/s00299-007-0449-x

[256] Y. Lee , A. M. Lloyd , S. J. Roux , Plant Physiol. 1999, 119, 989. 10069836 10.1104/pp.119.3.989 PMC32112

[257] F. Meggio , L. A. Pinna , FASEB J. 2003, 17, 349. 12631575 10.1096/fj.02-0473rev

[258] D. W. Litchfield , Biochem. J. 2003, 369, 1.12396231

[259] Y. Nagatoshi , M. Fujita , Y. Fujita , Planta 2018, 248, 571. 29799081 10.1007/s00425-018-2919-5 PMC6244648

[260] P. Hidalgo , V. Garretón , C. G. Berríos , H. Ojeda , X. Jordana , L. Holuigue , Plant Physiol. 2001, 125, 396. 11154347 10.1104/pp.125.1.396 PMC61020

[261] L. Di‐wu , L.‐L. Li , W.‐J. Wang , H.‐Z. Xie , J. Yang , C.‐H. Zhang , Q. Huang , L. Zhong , S. Feng , S.‐Y. Yang , J. Mol. Graphics Model. 2012, 36, 42. 10.1016/j.jmgm.2012.03.004 22516037

[262] M.‐S. Hung , Z. Xu , Y.‐C. Lin , J.‐H. Mao , C.‐T. Yang , P.‐J. Chang , D. M. Jablons , L. You , BMC Cancer 2009, 9, 135. 19419583 10.1186/1471-2407-9-135 PMC2696466

[263] A. Chilin , R. Battistutta , A. Bortolato , G. Cozza , S. Zanatta , G. Poletto , M. Mazzorana , G. Zagotto , E. Uriarte , A. Guiotto , L. A. Pinna , F. Meggio , S. Moro , J. Med. Chem. 2008, 51, 752. 18251491 10.1021/jm070909t

[264] J.‐K. Weng , M. Ye , B. Li , J. P. Noel , Cell 2016, 166, 881. 27518563 10.1016/j.cell.2016.06.027

[265] R. Zhou , A. J. Cutler , S. J. Ambrose , M. M. Galka , K. M. Nelson , T. M. Squires , M. K. Loewen , A. S. Jadhav , A. R. S. Ross , D. C. Taylor , S. R. Abrams , Plant Physiol. 2004, 134, 361. 14671016 10.1104/pp.103.030734 PMC316315

[266] G. L. Boyer , J. A. Zeevaart , Plant Physiol. 1982, 70, 227. 16662451 10.1104/pp.70.1.227 PMC1067117

[267] T. Kushiro , M. Okamoto , K. Nakabayashi , K. Yamagishi , S. Kitamura , T. Asami , N. Hirai , T. Koshiba , Y. Kamiya , E. Nambara , EMBO J. 2004, 23, 1647. 15044947 10.1038/sj.emboj.7600121 PMC391058

[268] G. C. Churchill , B. Ewan , M. J. Reaney , S. R. Abrams , L. V. Gusta , Plant Physiol. 1992, 100, 2024. 16653234 10.1104/pp.100.4.2024 PMC1075901

[269] T. Umezawa , M. Okamoto , T. Kushiro , E. Nambara , Y. Oono , M. Seki , M. Kobayashi , T. Koshiba , Y. Kamiya , K. Shinozaki , Plant J. 2006, 46, 171. 16623881 10.1111/j.1365-313X.2006.02683.x

[270] R. D. Hill , J. H. Liu , D. Durnin , N. Lamb , A. Shaw , S. R. Abrams , Plant Physiol. 1995, 108, 573. 12228494 10.1104/pp.108.2.573 PMC157376

[271] M. Okamoto , T. Kushiro , Y. Jikumaru , S. R. Abrams , Y. Kamiya , M. Seki , E. Nambara , Phytochemistry 2011, 72, 717. 21414645 10.1016/j.phytochem.2011.02.004

[272] A. J. Cutler , P. A. Rose , T. M. Squires , M. K. Loewen , A. C. Shaw , J. W. Quail , J. E. Krochko , S. R. Abrams , Biochemistry 2000, 39, 13614. 11063599 10.1021/bi0014453

[273] W. Hartung , A. Sauter , E. Hose , J. Exp. Bot. 2002, 53, 27. 11741037

[274] Z.‐J. Xu , M. Nakajima , Y. Suzuki , I. Yamaguchi , Plant Physiol. 2002, 129, 1285. 12114582 10.1104/pp.001784 PMC166522

[275] E.‐K. Lim , C. J. Doucet , B. Hou , R. G. Jackson , S. R. Abrams , D. J. Bowles , Tetrahedron: Asymmetry 2005, 16, 143.

[276] K. H. Lee , H. L. Piao , H.‐Y. Kim , S. M. Choi , F. Jiang , W. Hartung , I. Hwang , J. M. Kwak , I.‐J. Lee , I. Hwang , Cell 2006, 126, 1109. 16990135 10.1016/j.cell.2006.07.034

[277] Z.‐Y. Xu , K. H. Lee , T. Dong , J. C. Jeong , J. B. Jin , Y. Kanno , D. H. Kim , S. Y. Kim , M. Seo , R. A. Bressan , D.‐J. Yun , I. Hwang , Plant Cell 2012, 24, 2184. 22582100 10.1105/tpc.112.095935 PMC3442595

[278] A. Endo , M. Okamoto , T. Koshiba , in Abscisic Acid: Metabolism, Transport and Signaling (Ed: Zhang D.‐P.), Springer Netherlands, Dordrecht 2014, p. 21.

[279] Y. Todoroki , N. Hirai , H. Ohigashi , Tetrahedron 2000, 56, 1649.

[280] S. Saito , N. Hirai , C. Matsumoto , H. Ohigashi , D. Ohta , K. Sakata , M. Mizutani , Plant Physiol. 2004, 134, 1439. 15064374 10.1104/pp.103.037614 PMC419820

[281] T. D. Sharkey , K. Raschke , Plant Physiol. 1980, 65, 291. 16661176 10.1104/pp.65.2.291 PMC440313

[282] M. K. Walker‐Simmons , L. D. Holappa , G. D. Abrams , S. R. Abrams , Physiol. Plant. 1997, 100, 474.

[283] R. D. Hill , D. Durnin , L. A. K. Nelson , G. D. Abrams , L. V. Gusta , S. R. Abrams , Seed Sci. Res. 1992, 2, 207.

[284] D. C. Walton , B. Dorn , J. Fey , Planta 1973, 112, 87. 24469787 10.1007/BF00386035

[285] D.‐M. Xiong , Z. Liu , H. Chen , J.‐T. Xue , Y. Yang , C. Chen , L.‐M. Ye , J. Pharm. Anal. 2014, 4, 190. 29403882 10.1016/j.jpha.2014.01.004 PMC5761124

[286] S. M. Southwick , A. Chung , T. L. Davenport , J. W. Ryan , Plant Physiol. 1986, 81, 323. 16664801 10.1104/pp.81.1.323 PMC1075330

[287] A. Piotrowska , A. Bajguz , Phytochemistry 2011, 72, 2097. 21880337 10.1016/j.phytochem.2011.08.012

[288] E. A. Bray , J. A. Zeevaart , Plant Physiol. 1985, 79, 719. 16664479 10.1104/pp.79.3.719 PMC1074958

[289] H. Lehmann , K. Glund , Planta 1986, 168, 559. 24232333 10.1007/BF00392276

[290] M. López‐Carbonell , M. Gabasa , O. Jáuregui , Plant Physiol. Biochem. 2009, 47, 256. 19167901 10.1016/j.plaphy.2008.12.016

[291] R. Zhou , T. M. Squires , S. J. Ambrose , S. R. Abrams , A. R. S. Ross , A. J. Cutler , J. Chromatogr. A 2003, 1010, 75. 14503817 10.1016/s0021-9673(03)01029-x

[292] S. D. S. Chiwocha , S. R. Abrams , S. J. Ambrose , A. J. Cutler , M. Loewen , A. R. S. Ross , A. R. Kermode , Plant J. 2003, 35, 405. 12887591 10.1046/j.1365-313x.2003.01800.x

[293] A. Sauter , K.‐J. Dietz , W. Hartung , Plant Cell Environ. 2002, 25, 223. 11841665 10.1046/j.1365-3040.2002.00747.x

[294] A. Thameur , A. Ferchichi , M. López‐Carbonell , S. Afr. J. Bot. 2011, 77, 222.

[295] S. Wilkinson , W. J. Davies , Plant Cell Environ. 2002, 25, 195. 11841663 10.1046/j.0016-8025.2001.00824.x

[296] H. Kato‐Noguchi , Y. Tanaka , Plant Growth Regul. 2003, 40, 117.

[297] J. M. Harris , C. A. Ondzighi‐Assoume , Plant Signal. Behav. 2017, 12, e1273303. 28067583 10.1080/15592324.2016.1273303 PMC5289525

[298] A. A. Millar , J. V. Jacobsen , J. J. Ross , C. A. Helliwell , A. T. Poole , G. Scofield , J. B. Reid , F. Gubler , Plant J. 2006, 45, 942. 16507085 10.1111/j.1365-313X.2006.02659.x

[299] M. Okamoto , K. Tatematsu , A. Matsui , T. Morosawa , J. Ishida , M. Tanaka , T. A. Endo , Y. Mochizuki , T. Toyoda , Y. Kamiya , K. Shinozaki , E. Nambara , M. Seki , Plant J. 2010, 62, 39. 20088898 10.1111/j.1365-313X.2010.04135.x

[300] S. Saito , M. Okamoto , S. Shinoda , T. Kushiro , T. Koshiba , Y. Kamiya , N. Hirai , Y. Todoroki , K. Sakata , E. Nambara , M. Mizutani , Biosci., Biotechnol., Biochem. 2006, 70, 1731. 16819156 10.1271/bbb.60077

[301] Y. Todoroki , K. Kobayashi , H. Yoneyama , S. Hiramatsu , M.‐H. Jin , B. Watanabe , M. Mizutani , N. Hirai , Bioorg. Med. Chem. 2008, 16, 3141. 18164621 10.1016/j.bmc.2007.12.019

[302] N. Kitahata , S. Saito , Y. Miyazawa , T. Umezawa , Y. Shimada , Y. K. Min , M. Mizutani , N. Hirai , K. Shinozaki , S. Yoshida , T. Asami , Bioorg. Med. Chem. 2005, 13, 4491. 15882944 10.1016/j.bmc.2005.04.036

[303] W. Rademacher , Annu. Rev. Plant Physiol. Plant Mol. Biol. 2000, 51, 501. 15012200 10.1146/annurev.arplant.51.1.501

[304] J. Takeuchi , M. Okamoto , R. Mega , Y. Kanno , T. Ohnishi , M. Seo , Y. Todoroki , Sci. Rep. 2016, 6, 37060. 27841331 10.1038/srep37060 PMC5107945

[305] M. A. Correia , P. R. Ortiz de Montellano , in Cytochrome P450: Structure, Mechanism, and Biochemistry (Ed: Ortiz de Montellano P. R.), Springer US, Boston, MA 2005, p. 247.

[306] K. Ueno , Y. Araki , N. Hirai , S. Saito , M. Mizutani , K. Sakata , Y. Todoroki , Bioorg. Med. Chem. 2005, 13, 3359. 15848748 10.1016/j.bmc.2005.03.015

[307] M. Mizutani , Y. Todoroki , Phytochem. Rev. 2006, 5, 385.

[308] Y. Araki , A. Miyawaki , T. Miyashita , M. Mizutani , N. Hirai , Y. Todoroki , Bioorg. Med. Chem. Lett. 2006, 16, 3302. 16563759 10.1016/j.bmcl.2006.03.024

[309] S. Shukla , C.‐P. Wu , S. V. Ambudkar , Expert Opin. Drug Metab. Toxicol. 2008, 4, 205. 18248313 10.1517/17425255.4.2.205

[310] J. Santiago , F. Dupeux , A. Round , R. Antoni , S. Y. Park , M. Jamin , S. R. Cutler , P. L. Rodriguez , J. A. Marquez , Nature 2009, 462, 665. 19898494 10.1038/nature08591

[311] F. C. Peterson , E. S. Burgie , S. Y. Park , D. R. Jensen , J. J. Weiner , C. A. Bingman , C. E. Chang , S. R. Cutler , G. N. Phillips Jr. , B. F. Volkman , Nat. Struct. Mol. Biol. 2010, 17, 1109. 20729860 10.1038/nsmb.1898 PMC2933299

[312] A. S. Vaidya , F. C. Peterson , D. Yarmolinsky , E. Merilo , I. Verstraeten , S. Y. Park , D. Elzinga , A. Kaundal , J. Helander , J. Lozano‐Juste , ACS Chem. Biol. 2017, 12, 2842. 28949512 10.1021/acschembio.7b00650

[313] Z. Ren , Z. Wang , X. Zhou , H. Shi , Y. Hong , M. Cao , Z. Chan , X. Liu , E. Xu , J.‐K. Zhu , Sci. Rep. 2017, 7, 14022. 29070857 10.1038/s41598-017-14101-9 PMC5656587

[314] J. Frackenpohl , E. Grill , G. Bojack , R. Baltz , M. Busch , J. Dittgen , J. Franke , J. Freigang , S. Gonzalez , I. Heinemann , H. Helmke , M. Hills , S. Hohmann , P. von Koskull‐Döring , J. Kleemann , G. Lange , S. Lehr , T. Müller , E. Peschel , F. Poree , D. Schmutzler , A. Schulz , L. Willms , C. Wunschel , Eur. J. Org. Chem. 2018, 2018, 1403.

[315] F. Dupeux , J. Santiago , K. Betz , J. Twycross , S. Y. Park , L. Rodriguez , M. Gonzalez‐Guzman , M. R. Jensen , N. Krasnogor , M. Blackledge , M. Holdsworth , S. R. Cutler , P. L. Rodriguez , J. A. Marquez , EMBO J. 2011, 30, 4171. 21847091 10.1038/emboj.2011.294 PMC3199383

[316] M. Moreno‐Alvero , C. Yunta , M. Gonzalez‐Guzman , J. Lozano‐Juste , J. L. Benavente , V. Arbona , M. Menendez , M. Martinez‐Ripoll , L. Infantes , A. Gomez‐Cadenas , P. L. Rodriguez , A. Albert , Mol. Plant 2017, 10, 1250. 28736053 10.1016/j.molp.2017.07.004

[317] K. Miyazono , T. Miyakawa , Y. Sawano , K. Kubota , H. J. Kang , A. Asano , Y. Miyauchi , M. Takahashi , Y. Zhi , Y. Fujita , T. Yoshida , K. S. Kodaira , K. Yamaguchi‐Shinozaki , M. Tanokura , Nature 2009, 462, 609. 19855379 10.1038/nature08583

[318] K. Melcher , Y. Xu , L. M. Ng , X. E. Zhou , F. F. Soon , V. Chinnusamy , K. M. Suino‐Powell , A. Kovach , F. S. Tham , S. R. Cutler , J. Li , E. L. Yong , J. K. Zhu , H. E. Xu , Nat. Struct. Mol. Biol. 2010, 17, 1102. 20729862 10.1038/nsmb.1887 PMC2933329

[319] S. Koussevitzky , A. Nott , T. C. Mockler , F. Hong , G. Sachetto‐Martins , M. Surpin , J. Lim , R. Mittler , J. Chory , Science 2007, 316, 715. 17395793

[320] M. J. Cao , Y. L. Zhang , X. Liu , H. Huang , X. E. Zhou , W. L. Wang , A. Zeng , C. Z. Zhao , T. Si , J. Du , Nat. Commun. 2017, 8, 1183. 29084945 10.1038/s41467-017-01239-3 PMC5662759

[321] F. F. Soon , L. M. Ng , X. E. Zhou , G. M. West , A. Kovach , M. H. Tan , K. M. Suino‐Powell , Y. He , Y. Xu , M. J. Chalmers , J. S. Brunzelle , H. Zhang , H. Yang , H. Jiang , J. Li , E. L. Yong , S. Cutler , J. K. Zhu , P. R. Griffin , K. Melcher , H. E. Xu , Science 2012, 335, 85. 22116026 10.1126/science.1215106 PMC3584687

[322] Y. He , Q. Hao , W. Li , C. Yan , N. Yan , P. Yin , PLoS One 2014, 9, e95246. 24743650 10.1371/journal.pone.0095246 PMC3990689

[323] M. Nakagawa , M. Kagiyama , N. Shibata , Y. Hirano , T. Hakoshima , Genes Cells 2014, 19, 386. 24645846 10.1111/gtc.12140

[324] S. Han , M. K. Min , S. Y. Lee , C. W. Lim , N. Bhatnagar , Y. Lee , D. Shin , K. Y. Chung , S. C. Lee , B. G. Kim , S. Lee , Mol. Plant 2017, 10, 1190. 28827170 10.1016/j.molp.2017.08.003

[325] A. Vaidya , J. Helander , F. Peterson , D. Elzinga , W. Dejonghe , A. Kaundal , S.‐Y. Park , Z. Xing , R. Mega , J. Takeuchi , B. Khanderahoo , S. Bishay , B. Volkman , Y. Todoroki , M. Okamoto , S. Cutler , Science 2019, 366, eaaw8848. 31649167 10.1126/science.aaw8848

[326] S.‐Y. Park , F. Peterson , A. Mosquna , J. Yao , B. Volkman , S. Cutler , Nature 545, 2015, 520. 25652827 10.1038/nature14123

[327] J. Takeuchi , M. Okamoto , T. Akiyama , T. Muto , S. Yajima , M. Sue , M. Seo , Y. Kanno , T. Kamo , A. Endo , E. Nambara , N. Hirai , T. Ohnishi , S. Cutler , Y. Todoroki , Nat. Chem. Biol. 477, 2014, 10. 24792952 10.1038/nchembio.1524

[328] N. Rajagopalan , Biochemistry 2016, 55, 5155. 27523384 10.1021/acs.biochem.6b00605

